# A Novel Strategy for Selection and Validation of Reference Genes in Dynamic Multidimensional Experimental Design in Yeast

**DOI:** 10.1371/journal.pone.0038351

**Published:** 2012-06-04

**Authors:** Ayca Cankorur-Cetinkaya, Elif Dereli, Serpil Eraslan, Erkan Karabekmez, Duygu Dikicioglu, Betul Kirdar

**Affiliations:** Department of Chemical Engineering, Bogazici University, Bebek, Istanbul, Turkey; University of Jaén, Spain

## Abstract

**Background:**

Understanding the dynamic mechanism behind the transcriptional organization of genes in response to varying environmental conditions requires time-dependent data. The dynamic transcriptional response obtained by real-time RT-qPCR experiments could only be correctly interpreted if suitable reference genes are used in the analysis. The lack of available studies on the identification of candidate reference genes in dynamic gene expression studies necessitates the identification and the verification of a suitable gene set for the analysis of transient gene expression response.

**Principal Findings:**

In this study, a candidate reference gene set for RT-qPCR analysis of dynamic transcriptional changes in *Saccharomyces cerevisiae* was determined using 31 different publicly available time series transcriptome datasets. Ten of the twelve candidates (*TPI1*, *FBA1*, *CCW12*, *CDC19*, *ADH1*, *PGK1*, *GCN4*, *PDC1*, *RPS26A* and *ARF1*) we identified were not previously reported as potential reference genes. Our method also identified the commonly used reference genes *ACT1* and *TDH3*. The most stable reference genes from this pool were determined as *TPI1*, *FBA1*, *CDC19* and *ACT1* in response to a perturbation in the amount of available glucose and as *FBA1*, *TDH3*, *CCW12* and *ACT1* in response to a perturbation in the amount of available ammonium. The use of these newly proposed gene sets outperformed the use of common reference genes in the determination of dynamic transcriptional response of the target genes, *HAP4* and *MEP2*, in response to relaxation from glucose and ammonium limitations, respectively.

**Conclusions:**

A candidate reference gene set to be used in dynamic real-time RT-qPCR expression profiling in yeast was proposed for the first time in the present study. Suitable pools of stable reference genes to be used under different experimental conditions could be selected from this candidate set in order to successfully determine the expression profiles for the genes of interest.

## Introduction

Single time point experiments provide a static measurement of gene expression although transcription is a temporal process. Time series gene expression experiments are useful in elucidating the dynamic mechanism behind a biological process. The high-throughput data obtained from microarray technology enables the identification of differentially expressed genes. Real-time RT-qPCR is the favoured method for conducting a detailed study on a gene set of interest, which is determined from high-throughput studies. It is a widely preferred method for quantitative gene expression analysis due to its high sensitivity, specificity and wider dynamic range. However, data normalization for the elimination of sample-to sample differences is the main obstacle of this method even in non-transient studies [1], [2], [3], [4]. The multidimensional and temporal nature of time series data renders its analysis more complicated thus drawing attention to the importance of normalization.

Normalization with internal control genes, generally referred to as the housekeeping or reference genes, is a commonly used method among various normalization techniques for the determination of gene expression using real-time RT-qPCR. A suitable reference gene should be constitutively expressed in the tissues or cells under investigation regardless of the experimental perturbation. A single reference gene was frequently used in many studies in order to normalize mRNA fraction without any validation of stability. However growing evidence indicates the absence of a single universal reference gene, which may be independent of all kind of experimental conditions. Since the normalization of real- time RT-qPCR data using a non-validated single reference gene may engender misleading conclusions, in recent years, the calculation of a normalization factor based on the geometric average of validated multiple reference genes was suggested to discard possible outliers and differences in the abundance of different genes [5].

The general approach in normalization using multiple genes is the selection of reference genes among candidate genes, which have been commonly used for normalization. However a group of genes selected among the commonly used reference genes may not be a suitable reference gene set for each experimental condition. *ACT1*, which is a commonly used reference gene for *Saccharomyces cerevisiae*, was validated to be a suitable reference gene to normalize the expression of genes involved in central carbon metabolism in response to a short-term glucose pulse [6]. On the other hand, the use of *ACT1* and other commonly used reference genes *(PDA1, TDH3, RDN18)* was invalidated as internal control for quantitative analysis of gene expression in the yeast *S. cerevisiae* during long-term growth on glucose [4]. This fact clearly indicates the necessity of the validation of the suitability of reference genes under specific experimental conditions and steers us towards the determination of a candidate reference gene set among novel genes instead of commonly used ones, which is suitable for use under the conditions of interest.

A reasonably successful approach in the compilation of a candidate reference gene set is to select genes from a genome-wide background and to use a large number of microarray datasets for a specific condition and for specific tissues/organisms [1], [2], [7]. Since the determination of a suitable candidate gene set creates a big concern in dynamic expression profiling studies due to the multidimensional and dynamic nature of the experiments, microarray datasets could be used to identify the transcripts, which display stable expression with respect to time as well as to different genetic and environmental conditions. However, a vigilant human-mediated research is required for the selection of the microarray datasets, which will be used for the identification of the candidate reference gene set.

Following the compilation of the candidate reference gene set, the second key point is the determination of the most stable genes among the candidates under selected conditions. Several software tools were developed for the verification of the suitability of the candidate reference genes such as geNorm [5], NormFinder [8] and Bestkeeper [9]. As designated by the geNorm algorithm, the expression ratio of two suitable reference genes should be constant across all samples. Therefore the inclusion of two correlated genes in the list of candidates may lead to false positive results due to the similarity in their expression profiles. On the other hand, the NormFinder algorithm depends on statistical linear mixed-effects modelling, which accounts for the overall variation in the expression of the candidate reference genes and also for the variation between the sample groups. NormFinder was found to be less sensitive in the case of correlated gene sets. Although the definition of “stability” differs somehow among these algorithms, there are many studies reporting similar results in terms of stability when either one of these software were used [3], [10], [11].

The aim of the present study was to determine a set of candidate reference genes to analyze the dynamic transcriptional response of selected genes to changing environmental conditions by real-time RT-qPCR in *S. cerevisiae*. For this purpose, a set of candidate reference genes, which can be used for the dynamic expression profiling studies in *S. cerevisiae*, was determined for the first time using 31 publicly available and independently generated time-dependent transcriptome data.

This candidate reference gene set was then verified using two separate datasets to identify the most stable reference genes under these two selected environmental conditions and the stability of the reference sets were confirmed by expression analysis of the specific perturbation responsive genes. The stable reference gene sets comprised of both previously reported reference genes and genes that were identified as reference for the first time in this study. Moreover these newly identified genes were determined to be more stable than the common reference candidates, used either individually or as a multiple gene set, presenting an improvement in the determination of gene expression profiles.

This study revealed that the selection of reference genes among the candidate reference gene set, which was identified in this present study, outperformed the use of reference genes that were frequently reported in the literature. This novel approach allows the reduction of multiple parameters that would be frequently encountered in dynamic gene expression studies down to only one parameter, time, for the determination of a suitable set of reference genes. The high flexibility of this method enables the use of this candidate reference gene set in the determination of reference genes under any experimental condition as long as the supplemented data is of dynamic nature.

## Results

### Identification of a candidate reference gene set

31 different time series datasets from experiments conducted using *S. cerevisiae* strains were collected from the publicly available database of functional genomics experiments; ArrayExpress [12] (Table S1). The coefficient of variation (CV), which is the ratio of standard deviation to mean, was used to compare the extent of variation among the expression levels of genes and therefore to determine the transcripts, which displayed a stable expression profile throughout different time points. The ranking of the CV values was defined as the stability profile in this study. Two different approaches were used to determine the candidate reference gene set based on stability profiles.

In the first approach, each of the 31 datasets was analyzed separately in order to obtain the individual stability profiles. The top 100 stable genes were identified for each dataset. 4156 transcripts did never rank within the top 100 genes in any of these datasets and interestingly there was no common unique transcript, which appeared among the top 100 stable genes in all cases. *TPI1* and *ACT1* were among the 100 most stable transcripts in 81% of the cases (Figure S1). The 10 transcripts, *TPI1*, *ACT1*, *TDH3*, *FBA1*, *CCW12*, *CDC19*, *ADH1*, *PGK1*, *GCN4* and *PDC1*, which ranked within the top 100 in more than 55% of the datasets, were selected as candidate reference genes. Among these genes *ACT1*, which encodes the single essential gene for actin, and *TDH3*, which encodes glyceraldehyde-3-phosphate dehydrogenase (GAPDH), were reported to be commonly used reference genes in yeast [13]. *CCW12*, the cell wall mannoprotein with a role in the maintenance of newly synthesized areas of cell wall; *GCN4*, the transcriptional activator of amino acid biosynthetic genes in response to amino acid starvation, and other genes functioning in the super pathway of glucose fermentation were identified in this candidate reference gene set [14].

As a second approach, the stability profiles of the genes were investigated by combining different datasets in order to identify the genes, which display less fluctuating expression profiles across different time points and under different experimental conditions. The expression values of the genes that are common in all datasets were used to construct the combined dataset. At this point a question of whether there were a prerequisite number of experiments to be included into this analysis arose. In order to observe the effect of the number of datasets on stability profiles of the genes, resampling from the data was carried out via bootstrapping. Random combinations of 31 datasets were generated. The stability profiles for each of these combinations were calculated and the similarities between these stability profiles and the overall stability profile, which was obtained by combining all 31 datasets, were assessed using the Pearson correlation coefficient (PCC). Average of the PCC values were calculated for the combinations having the same number of datasets. The average and the minimum of the PCC values of the combinations that have the same number of datasets were examined as a function of the number of datasets (Figure S2). The analysis of these combinations showed that both the average and the minimum PCC increased with the inclusion of increasing numbers of datasets. This observation indicated that, each dataset included in the analysis has a contribution to the stability profile. Therefore the combination of all available data sets was used in the second approach.

The stability profile of this combined dataset, which consists of the expression values of 5423 transcripts across 888 different time points, revealed the genes displaying fewer fluctuations in their expression profiles across all these time points. The top 100 genes displaying the lowest CV value were investigated for the identification of candidate reference genes. The CV values of these genes varied between 0.05 and 0.11. A binned frequency histogram of the available data indicated that a gene pool of 6 genes with the lowest CV values (corresponding to 1% of the total population) would be a suitable choice of candidate pool (Figure S3). Bringing the CV threshold above 0.07 resulted in an observable number of additional genes thus populating the candidate pool with genes having less stable expression profiles, which are likely to be discarded in a stability analysis and rendering the experiments costly. These genes were *TDH3*, *RPS26A*, *TPI1*, *CDC19*, *ARF1* and *ENO1*. Due to the high homology between *ENO1* and *ENO2* (blastn e-value = 4.7 e-264), concerns regarding a possible unspecificity in the PCR product led to the elimination of this gene from the candidate set. Therefore the top five genes, *TDH3*, *RPS26A*, *TPI1*, *CDC19* and *ARF1* with the smallest coefficient of variation values were selected as the candidate reference genes. Among these genes, *TDH3*, *CDC19* and *TPI1* were also identified as candidate genes using the first approach. *RPS26A* encoding a protein component of the small ribosomal subunit (40S) has similarity to rat S26 ribosomal protein, which is commonly used as a reference gene in rat tissues [15]. The wheat ortholog of *ARF1*, which encodes an ADP-ribosylation factor, was shown to be one of the most stable reference genes in that particular plant [3].

The candidate reference genes, which were selected using two different approaches, were combined and a total of 12 transcripts were determined as the candidate reference gene set for normalization of dynamic real-time RT-qPCR data.

### Identification and verification of exclusive reference gene subsets under specific experimental conditions

In order to test the applicability of this candidate reference gene set on different experimental conditions, this pool of genes were used to identify the most stable condition specific reference genes. For this purpose, perturbations involving nutrient availability in yeast were investigated. Yeast cells grown in glucose-limited (Case study I) or ammonium-limited (Case study II) continuous cultures at steady state were supplemented with the respective limiting nutrient in an impulse-like manner. Dynamic gene expression profiles of the candidate reference genes were investigated in response to recovery from limiting conditions. The expression levels of *HAP4* in Case study I and *MEP2* in Case study II were investigated using a reference gene set, which was determined from the available candidate genes. *HAP4* and *MEP2* were selected such that their expression profiles were responsive to variations in the amount of available glucose and ammonium, respectively. Primers of 12 candidates were designed and their amplification efficiencies were determined. All candidates except for *PGK1*, whose amplification efficiency could not be improved to be higher than 85%, were procured for further analysis (Table S2). Regions from the 11 candidate reference genes as well as the query genes were PCR amplified and the raw Cq values were transformed into relative quantities using the delta-Ct method [3]. All analyses in these case studies were conducted in compliance with MIQE guidelines for qPCR (Table S3) [16]. The expression stability of these candidates throughout these time series experiments was tested using two different statistical software programs; geNorm and NormFinder.

### Case Study I: Selection and validation of the most stable reference genes during glucose perturbation

The presence of any correlated gene pairs among the candidates was previously reported to engender misleading predictions regarding the stability order of the candidate reference genes [5]. In order to identify correlated gene pairs from the candidate list, Pearson correlation coefficients between expression values of each pair of the candidates were calculated (Table S4). The threshold for the correlation among gene pairs was decided as ±0.85. A total of three correlated gene pairs were identified. *ARF1* was correlated with two other genes; *TPI1* and *CDC19*. The third correlated gene pair was *FBA1* and *CCW12*. Five different pools of candidate genes were created in order to investigate the effect of the inclusion of correlated gene pairs in the reference gene set. P-0 was the pool including all candidate genes. P-1 and P-3 were created by excluding *ARF1* from the candidate list together with either *CCW12* or *FBA1*, respectively. As an alternative, *ARF1* was kept in the pool and the other two transcripts (*TPI1* and *CDC19*), which showed high correlation with *ARF1*, were excluded from the candidate list in addition to either *CCW12* or *FBA1* (P-2 and P-4). One other alternative pool was constructed by excluding all of these correlated genes from the candidate list (P-5).

In order to identify a suitable set of reference genes, two algorithms; NormFinder and geNorm were used. Both algorithms were used to rank the eleven candidates based on the stability of their expression profiles. Additionally, geNorm algorithm was also used to determine the stability ranking of genes in the pools, in which only the genes with a correlation of <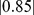 among their expression profiles were left (P1–P5). The genes were scored using the average value of the stability rankings obtained by NormFinder and geNorm for each pool separately. The total score for each candidate gene was calculated by summing up its individual scores for each case; P-0 to P-5. The genes were then ranked based on their total scores from the minimum score to the maximum score indicating an order from the most stable to the least stable gene. The minimum number of reference genes to be used was selected as 4 in order to make sure that at least one stable gene could be selected even after the removal of correlated gene pairs in P-4 and P-5 (Figure S4). This ranking indicated that the lowest total scores were obtained for *TPI1*, *FBA1*, *CDC19* and *ACT1*.

**Figure 1:**
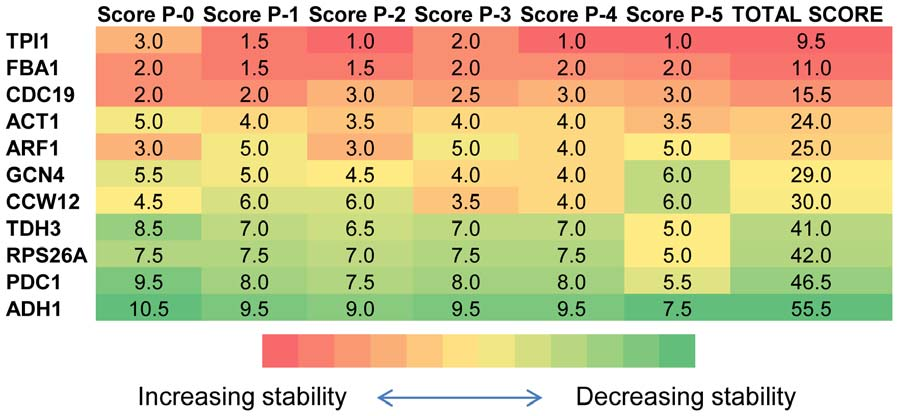
Scoring of the candidate genes in Case Study I. The cells representing the scores of the genes in different pools were colour coded. A coding scale from red to green indicated decreasing stability. The score of the genes in each pool was indicated by the number assigned to that cell in a quantitative manner; the smallest score indicating the most stable gene and the largest score indicating the least stable one. The stability of the gene associated with that score was also indicated by the colour assigned to that cell in a qualitative manner. The scoring of the six different pools of candidate genes was determined as the average ranking for each gene as indicated by the NormFinder and the geNorm analyses and the results were provided in the first six columns. The analysis was carried out using all genes (P-0), and using the 5 pools, each excluding a different set of correlated genes (P-1 to P-5). The total score for each gene was provided in the last column by summing up all of its scores through P-0 to P-5.

The scores for P-1 and P-2 were the lowest for these four reference genes indicating that these pools provided the optimal configuration. Since two of the most stable genes; *TPI1* and *CDC19*, were removed in P-2 in order to discard the correlations among gene pairs, the scoring for these genes was based solely on the stability ranking obtained from NormFinder. The reference gene selection was carried out based on the stability scoring that was provided for P-1 since both geNorm and NormFinder stability rankings were available for all of these four genes. (Figure 1, Figure S4, Figure S5). *TPI1*, *FBA1*, *CDC19* and *ACT1* were also determined as the four most stable genes among all candidates when the NormFinder algorithm was used to perform the stability analysis.

Exclusion of the correlated gene pairs resulted in differences in the stability orders of the candidate genes when geNorm was used. The comparison of the stability rankings of P-0 and P-1 by geNorm with the rankings obtained by NormFinder indicated that elimination of correlations by the removal of *ARF1* and *CCW12* resulted in obtaining similar results using both algorithms. *TPI1* and *ACT1* could not be identified as stable unless correlations that were present among the expression profiles of the genes in the pool were removed. These results indicated that, correlated gene pairs should be excluded from the candidate list when using geNorm in order to avoid misleading results during the determination of the stable reference genes. Exclusion of the correlated gene pairs facilitates the attainment of comparable results by both algorithms. These two algorithms thus could be used to cross-check conjoint results while selecting the most suitable reference genes.

geNorm suggested using a varying number of reference genes between 3 and 6 for different pools (with a pairwise variation threshold of 0.15 as suggested by the software developers) for determining the expression profile of *HAP4* (Figure S6). A set of at most six reference genes; *TPI1*, *FBA1*, *CDC19*, *ACT1*, *ARF1* and *GCN4* were determined to constitute a suitable pool for the analysis of gene expression in response to an impulse-like glucose perturbation. The change in the expression level of *HAP4* with a known transcriptional response to the amount of glucose in the medium was investigated in order to test the performance of this reference gene pool. *HAP4*, whose gene product is a transcriptional activator and global regulator of respiratory gene expression, was known to be repressed by glucose in order to prevent the activation of respiration [17]. Therefore it could be expected that *HAP4* would show a decreasing expression profile in response to a sudden increase in the amount of available glucose in the medium.

**Figure 2:**
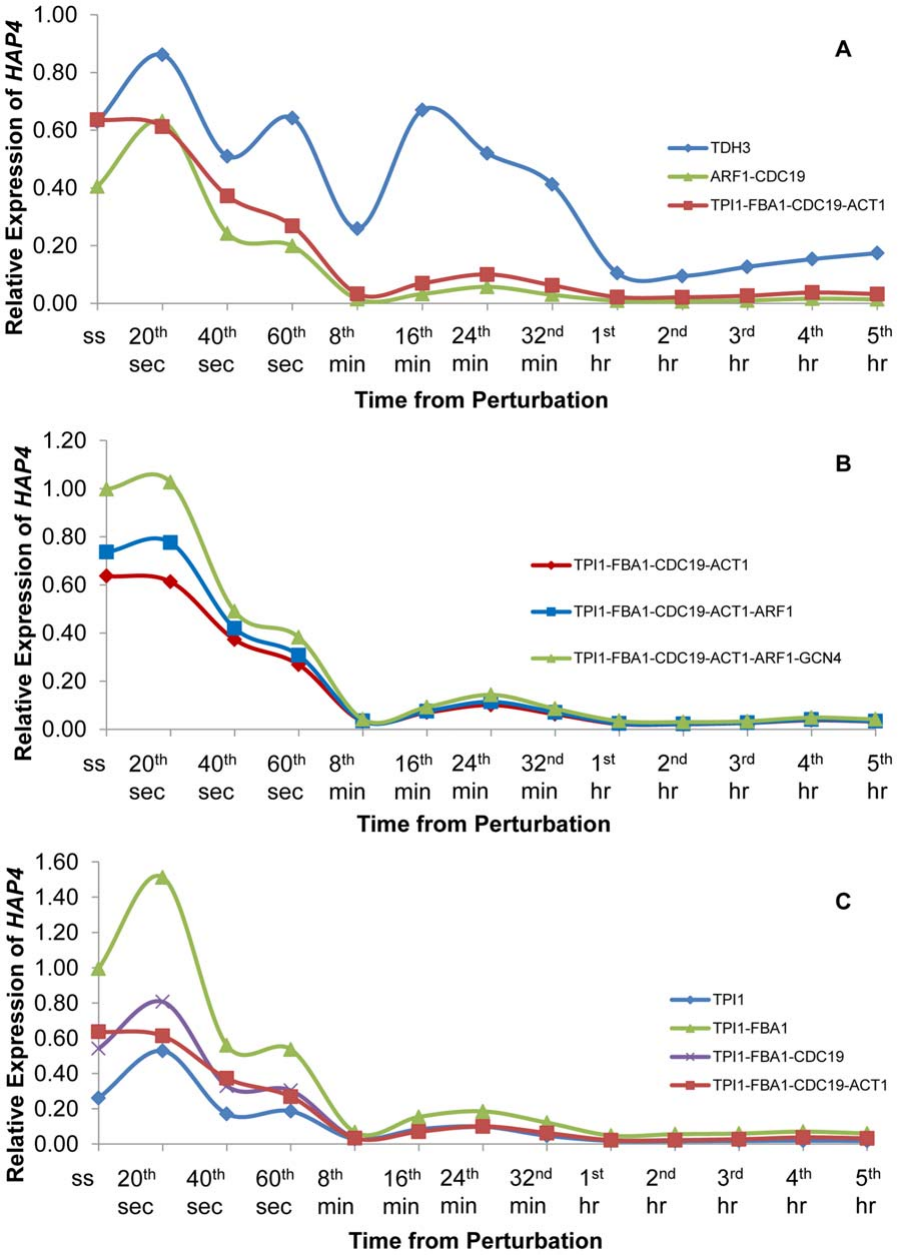
Expression profile of *HAP4* in response to the relaxation from glucose limitation. This figure represents the expression profile for *HAP4* using different combinations of reference genes, which were selected from the candidate pool. The expression profiles were calculated using *TDH3*; a commonly selected reference gene, using *ARF1* and *CDC19*; the two most stable genes identified from P-0 by geNorm, and using *TPI1*, *FBA1*, *CDC19* and *ACT1*; the final set of most stable genes (A). The contribution of increasing the number of genes in the final reference set to the expression profile of *HAP4* was investigated by adding *ARF1* and *GCN4* to the reference gene set in the given order (B). The effect of reducing number of genes in the final reference gene set on the expression profile of *HAP4* was investigated. The number of reference genes included in the set was reduced by one each time starting from the least stable gene in the set (C). For each individual profile, the reference genes, which were used in the determination of *HAP4* expression, were indicated by different colours in the legend.

In order to evaluate the importance of determining a suitable reference gene set, which would be used in the normalization of the expression profile of the gene of interest, three different sets of reference genes were used to calculate the relative expression profile of *HAP4*. Initially *TDH3*; one of the commonly used reference genes was used as the sole gene for normalization. Then, the geometric average of the Ct values for *ARF1*-*CDC19*; the gene pair, which wasidentified as the most stable pair in the analysis conducted by geNorm using P-0 was used as the reference gene set for the normalization of *HAP4* expression. In the third case, *TPI1*, *FBA1*, *CDC19* and *ACT1*; the reference genes identified as the most stable genes both by NormFinder and by geNorm using P-1 were employed in the analysis. Similarly the geometric average of the Ct values of these four genes was used for determining the expression profile of *HAP4*. *HAP4* displayed a fluctuating expression profile in response to glucose perturbation when the data was normalized using *TDH3*; a commonly used reference gene. Although normalization using *ARF1* and *CDC19* captured the general trend for *HAP4* expression, the sudden increase within the first minute upon addition of glucose was an unacceptable outcome of this selection of reference genes. In contrast, the relative expression of *HAP4* decreased immediately after the impulse and remained repressed during the time course of the experiment as expected when the four most stable reference genes (*TPI1*, *FBA1*, *CDC19*and *ACT1*) were used for normalization (Figure 2-a). These results clearly demonstrated that using a commonly preferred reference gene without verification would yield to misleading expression profiles. Moreover the selection of a correlated gene pair would not improve the outcome even though they were identified to be stable by commonly used reference selection software.

The *TPI1*-*FBA1*-*CDC19*-*ACT1* reference gene set was further analyzed in order to investigate the effect of having additional genes in the set or the effect of excluding one or more of the members of the set. Since the pairwise variations among the candidate reference genes that were obtained from the geNorm algorithm indicated that a set of six reference genes would be suitable, the contribution of the next fifth and the sixth most stable reference genes in determining the expression profile of *HAP4* were investigated. The results indicated that the inclusion of *ARF1* or *ARF1* and *GCN4* in the reference gene set did not yield any improvements in the determination of the expression profile of *HAP4*. Moreover, the expression trend of *HAP4* was observed to change slightly unfavourably upon addition of *ARF1* in the reference gene set. Further inclusion of *GCN4* in the reference set yielded a similar undesirable outcome (Figure 2-b). Therefore a pool consisting of at most four reference genes was determined to be sufficient.

The change in the gene expression profile within the first minute upon inclusion of ARF1 in the reference gene set was similar to the response that was observed when the *ARF1*-*CDC19* gene pair was used as reference. The common denominator in the *ARF1*-*CDC19* reference gene set and the *TPI1*-*FBA1*-*CDC19*-*ACT1* reference gene set was *CDC19*, which might indicate that the general trend in *HAP4* expression profile was observed owing to the presence of this gene. The unexpected increase in the gene expression at the 20^th^ second post impulse was observed due to the inclusion of *ARF1* in the reference gene set (Figure 2-a, 2-b).

The observation that the inclusion of the more genes in the reference set did not provide any improvement led to the thinking that the reference gene set would be further reduced in size. For this purpose, the number of reference genes included in the set was reduced by one each time starting from the least stable gene in the set. The exclusion of *ACT1* from the reference gene set resulted in an unexpected increase at the 20^th^ second post impulse. A further exclusion of *CDC19* from the set caused an even steeper increase in *HAP4* expression at the 20^th^ second post impulse in addition to unexpected increases in gene expression in the 60^th^ second and another slight increase at the 24^th^ minute. Utilization of *TPI1* as the sole reference gene for the normalization of *HAP4* expression yielded a similar expression profile for this gene to that of using the geometric average of the Ct values for *TPI1*, *FBA1* and *CDC19* (Figure 2-c). The increase in the expression of *HAP4* at the 20^th^ second could only be prevented via the inclusion of *ACT1* in the reference gene set raised a question of whether this gene could be used as a single reference gene in determining the expression profiles under the stated conditions. Moreover, *ACT1* was frequently reported as a reference gene in yeast gene expression studies via real-time RT-qPCR [6]. However, the use of *ACT1* as the sole reference gene did not yield a decreasing expression profile for *HAP4*. The similarity between the expression profiles when *TPI1* was used alone or in conjunction with *FBA1* and *CDC19* aroused curiosity regarding whether using the geometric average of the Ct values for *FBA1* and *CDC19* alone would result in an adequate expression profile for *HAP4*. However this reference gene pair caused a steep increase in *HAP4* expression at the 20^th^ second post impulse (Figure S7). These results indicated that the reference gene set comprised of *TPI1*, *FBA1*, *CDC19*, *ACT1* was suitable for determining gene expression in response to an impulse-like perturbation in glucose levels.

### Case Study II: Selection and validation of the most stable reference genes during ammonium perturbation

In order to identify correlated gene pairs from the candidate list, Pearson correlation coefficients between expression values of each pair of the candidates were calculated. Analysis of the PCCs between the gene pairs among the candidate gene set revealed the presence of one gene pair with a PCC greater than 0.85 in response to relaxation of ammonium limitation (Table S5). *TDH3* was correlated with *PDC1*. Three different pools of candidate genes were formed in order to investigate the effect of the inclusion of correlated gene pairs in the reference gene set. P-0 was the pool including all candidate genes. P-1 and P-2 were created by excluding *TDH3* and *PDC1* from the candidate list, respectively. One other alternative pool was constructed by excluding both of these correlated genes from the candidate list (P-3).

The eleven candidates were ranked based on the stability of their expression profiles using NormFinder and geNorm as it was the case for the glucose perturbation. Additionally, geNorm algorithm was also used to determine the stability ranking of genes in the pools, in which only the genes with a correlation of <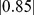 among their expression profiles, were left (P-1 to P-3). The genes were scored such that the average stability rankings were obtained using NormFinder and geNorm for each pool separately. The total score for each candidate gene was calculated by summing up its individual scores for each case; P-0 to P-3. The genes were then ranked based on their total scores from the minimum score to the maximum score indicating an order from the most stable to the least stable gene. Although the minimum number of reference genes to be used could be selected as 3 in order to make sure that at least one stable gene remained even after the removal of the correlated gene pair in P-3, the number of reference genes was selected as 4 for the sake of consistency with Case Study I (Figure S8). Indeed the analysis conducted by geNorm also demonstrated that 4 genes should be used as reference under the stated conditions (Figure S9). This ranking indicated that the lowest total scores were obtained for *FBA1*, *TDH3*, *ACT1* and *CCW12*.

**Figure 3:**
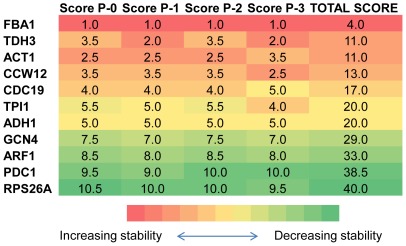
Scoring of the candidate genes in Case Study II. The cells representing the scores of the genes in different pools were colour coded. A coding scale from red to green indicated decreasing stability. The score of the genes in each pool was indicated by the number assigned to that cell in a quantitative manner; the smallest score indicating the most stable gene and the largest score indicating the least stable one. The stability of the gene associated with that score was also indicated by the colour assigned to that cell in a qualitative manner. The scoring of the four different pools of candidate genes was determined as the average ranking for each gene as indicated by the NormFinder and the geNorm analyses and the results were provided in the first six columns. The analysis was carried out using all genes (P-0), and using the 3 pools, each excluding a different set of correlated genes (P-1 to P-3). The total score for each gene was provided in the last column by summing up all of its scores through P-0 to P-3.

In this case, geNorm did not capture the gene pair with the highest correlation as the most stable pair. However, although the most stable gene was identified as *FBA1* and the 4 least stable genes were identified as *GCN4*, *ARF1*, *PDC1* and *RPS26A* by both geNorm and NormFinder, the stability orders for the remaining of the set were considerably different by either one of the algorithms. Removal of the correlation among the candidate genes did not change the stability rankings determined by geNorm such that both algorithms would yield similar stability profiles. If the three candidate pools (P-1 to P-3) were analyzed, *FBA1* and *ACT1* would fall into the stable gene set as it was the case that was determined by NormFinder (Figure 3, Figure S8, Figure S10). *FBA1*, *TDH3*, *CCW12* and *ACT1* were determined as the four most stable genes among all candidates as indicated by their scores (Figure 3).

A set of four reference genes; *FBA1*, *TDH3*, *CCW12* and *ACT1* were determined to constitute a suitable pool for the analysis of gene expression in response to an impulse-like ammonium perturbation. The change in the expression level of *MEP2*, an ammonium transporter, was investigated in order to test the performance of this reference gene pool. *MEP2*, whose mRNA accumulation was more abundant in cells grown in limiting concentrations of ammonia than in high concentrations [18] was monitored in response to an impulse-like addition of ammonium into its limited culture. Therefore it could be expected that *MEP2* would display a decreasing expression profile in response to a sudden increase in the amount of available ammonium in the medium.

**Figure 4:**
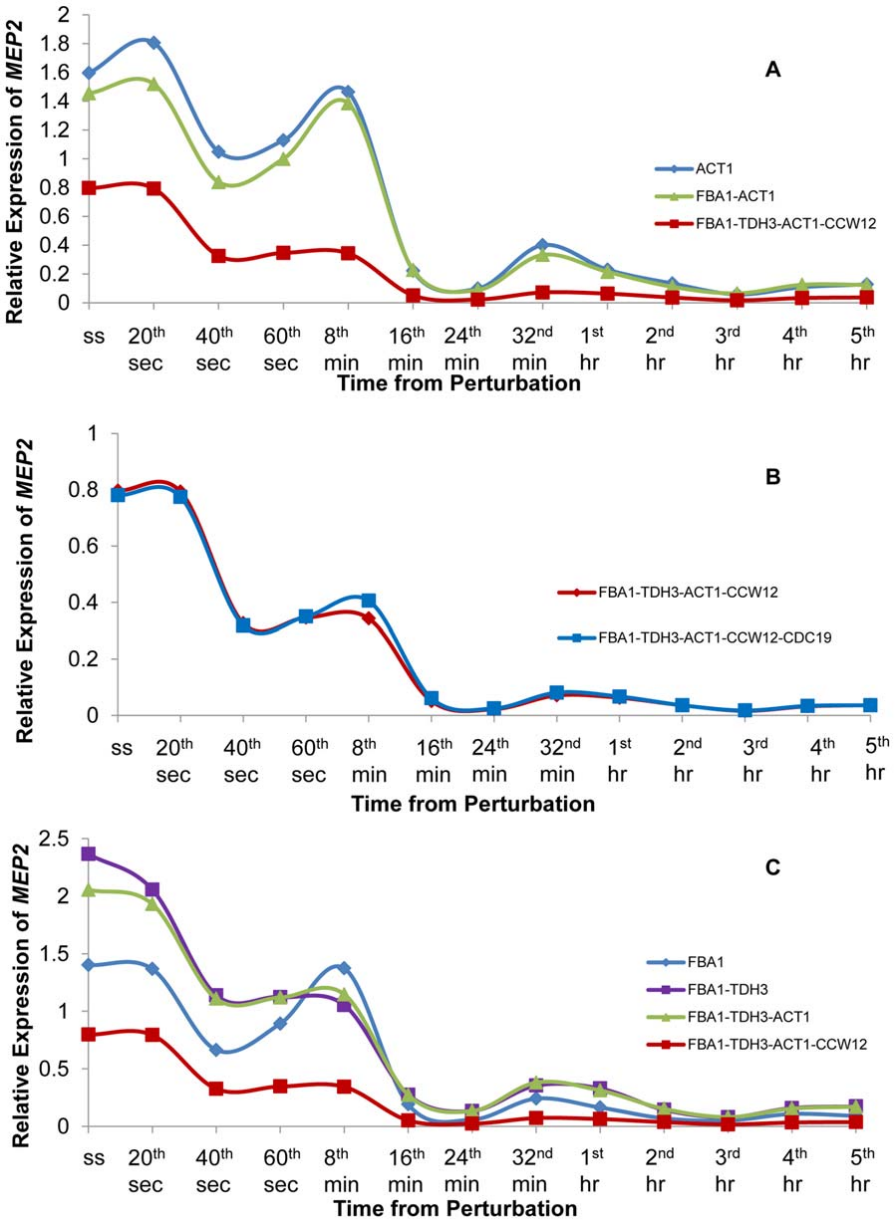
Expression profile of *MEP2* as a response to the relaxation from ammonium limitation. This figure represents the expression profile for *MEP2* using different combinations of reference genes, which were selected from the candidate pool. The expression profiles were calculated using *ACT1*; a commonly selected reference gene, using *FBA1* and *ACT1*; the two most stable genes identified from P-0 by geNorm, and using *FBA1*, *TDH3*, *ACT1* and *CCW12*; the final set of most stable genes (A). The contribution of increasing the number of genes in the final reference set to the expression profile of *MEP2* was investigated by adding *CDC19* to the reference gene set (B). The effect of reducing number of genes in the final reference gene set on the expression profile of *MEP2* was investigated. The number of reference genes included in the set was reduced by one each time starting from the least stable gene in the set (C). For each individual profile, the reference genes, which were used in the determination of *MEP2* expression, were indicated by different colours in the legend.

In order to evaluate the importance of determining a suitable reference gene set, which would be used in the normalization of the expression profile of the gene of interest, three different sets of reference genes were used to calculate the relative expression profile of *MEP2*. Initially *ACT1*; one of the commonly used reference genes was used as the sole gene for normalization. Then, the geometric average of the Ct values for *FBA1*-*ACT1*; the gene pair, which were identified as the most stable pair in the analysis conducted by geNorm using P-0 was used as the reference gene set for the normalization of *MEP2* expression. In the third case, the geometric average of the Ct values of *FBA1*, *TDH3*, *CCW12* and *ACT1*; the reference genes identified as the most stable genes based on the scores, which were calculated using both NormFinder and geNorm results, was used for determining the expression profile of *MEP2*. *MEP2* displayed a fluctuating expression profile in response to ammonium perturbation when the data was normalized using *ACT1*; a commonly used reference gene or using the most stable gene *FBA1* along with *ACT1*. In contrast, the relative expression of *MEP2* decreased gradually following the impulse and remained repressed during the time course of the experiment as expected when the four most stable reference genes (*FBA1*, *TDH3*, *CCW12* and *ACT1*) were used for normalization (Figure 4-a). These results clearly demonstrated that using a commonly preferred reference gene without verification would yield to misleading expression profiles.

The *FBA1*-*TDH3*-*CCW12*-*ACT1* reference gene set was further analyzed in order to investigate the effect of having additional genes in the set or the effect of excluding one or more of the members of the set. The contribution of the fifth most stable reference genes in determining the expression profile of *MEP2* was investigated. The results indicated that the inclusion of *CDC19* in the reference gene set did not yield any improvements in the determination of the expression profile of *MEP2* displaying an unexpected increase in gene expression at the 8^th^ minute (Figure 4-b). Therefore a pool consisting of at most four reference genes, as also suggested by geNorm, was determined to be sufficient.

The observation that the inclusion of the more genes in the reference set did not provide any improvement led to the thinking that the reference gene set would be further reduced in size as it was in Case Study I. For this purpose, the number of reference genes included in the set was reduced by one each time starting from the least stable gene in the set. The exclusion of *CCW12* or *CCW12* and *ACT1* from the reference gene set resulted in an unexpected increase in gene expression during a period from approximately the 32^nd^ minute until the 2^nd^ hour post impulse. A further exclusion of *TDH3* from the set, utilizing *FBA1* as the sole reference gene, caused a fluctuating expression profile for *MEP2* (Figure 4-c). Two of the most commonly used housekeeping genes *ACT1* and *TDH3* were included in the reference gene set. A further analysis was conducted to investigate whether using the geometric average of the Ct values for these two genes would yield a non-fluctuating and decreasing expression profile for *MEP2*. However, it was observed that an increase in *MEP2* expression during a period from approximately the 32^nd^ minute until the 2^nd^ hour post impulse was present indicating that *FBA1* and *CCW12* were both essential in acquiring a smooth expression profile (Figure S11).

### Determination of the optimum number of reference genes for normalization

The geNorm algorithm provides the contribution of each additional stable gene into the reference gene set in terms of the pairwise variation between the two sequential normalization factors [5]. The developers suggest using a threshold of 0.15 in the determination of the optimal number of reference genes required for normalization. geNorm suggested using a different number of reference genes varying between 3 and 6 for each pool in the case of glucose perturbation (Table S6 and Figure S6). This result necessitated a human-mediated evaluation of the expression profiles of the gene of interest obtained by different sets of reference genes. The results presented in this study indicated that the utilization of a set comprised of four reference genes with stable and uncorrelated expression profiles would be sufficient for this case. On the other hand, in the case of ammonium perturbation, the analysis using geNorm revealed that the optimal number of reference genes was 4 for each pool (Table S6 and Figure S9). Further analysis also demonstrated that neither increasing nor decreasing the number of reference genes improved the expression profile obtained for *MEP2*.

Therefore, the number of reference genes suitable for real-time RT-qPCR data normalization would also vary on a case specific manner and a human-mediated evaluation of the obtained results to design real-time RT-qPCR experiments may provide reductions in the number of experiments, thus reducing the unnecessary costs.

### Stability of the expression profile of the reference gene sets along a dynamic experimental condition

In order to demonstrate the stability of the reference genes that were identified in each case study, an external control gene would be required in the analysis such that the expression of these reference genes were shown to be constant along a dynamic experimental condition. 18S rRNA was believed to be a suitable choice of selection as reference. 18S rRNA was frequently reported among the candidate reference genes that were used in real-time RT-qPCR analysis [10], [11], [19], [20], [21], [22], [23], [24]. With its low turnover rate and large abundance, 18S rRNA was reported as an attractive choice as a reference gene [19].

However, the results of real-time RT-qPCR analysis indicated that 18S rRNA was the least stable candidate based on the rankings identified by both NormFinder and geNorm for both cases investigated in the present study (Figure S12). This outcome led to the investigation of the raw Ct values as a measure of stability as reported previously [25]. The Ct values were analysed for 18S rRNA along with the Ct values for two stable genes; *TPI1* and *CDC19* for Case Study I and *FBA1* and *TDH3* for Case Study II, the least stable gene for each case; *ADH1* for Case Study I and *RPS26A* for Case Study II, as well as the geometric average of the Ct values for the reference gene set that was determined to be suitable for each case that was presented above. The results indicated that the response by 18S rRNA fluctuated the most together with the genes identified as the least stable (Figure S13). Indeed other studies also reported that 18S rRNA was not a preferable choice as a reference gene due to several factors including its high abundance introducing human errors during dilution [19] and its regulation by RNA polymerase I rather than RNA polymerase II, which regulated the expression of mRNA in the cell [26]. Because of these concerns the use of 18S rRNA as reference was not preferred in several studies [20], [22], [27], [28], [29]. In fact this issue of the evaluation of the expression stability of a candidate was addressed as a circular problem in several studies [8], [30].

In order to overcome this problem the data was normalized across average of the Ct values of all genes at each time point. Although this might introduce a bias as a result of the overall trend captured in the data, it should be noted that at times when the commonly used reference genes fail to display a stable expression under various experimental conditions, this strategy would be undertaken for data specific validation of reference genes [31], [32]. The variation in the expression profile of the reference gene sets was the lowest in each case study in comparison to the variations in the expression profiles of the genes whose Ct value profiles were investigated in Figure S13. The expression profile of the gene set was more stable across time than the expression profiles of its individual members and the least stable profiles were observed for the gene that was identified as the least stable in each case as well as for 18S rRNA [Figure S14].

## Discussion

Understanding the dynamic mechanism behind a biological process and the identification of the statistically meaningful changes in the expression levels of selected genes involved in the various processes require quantification and comparison of dynamic data. Real-time RT-qPCR is widely used to investigate the relative expression levels of target genes in detail. However, the selection of suitable reference genes imposes problems in the analysis of both transient and non-transient expression profiling studies. The quantification of the dynamic response by real-time RT-qPCR requires the identification of the reference genes, which display constant expression across all time points regardless of the different genetic or environmental perturbations. Although the commonly used reference genes previously reported in literature [4], [5], [6] constitute a suitable pool for the analysis of non-transient data, these genes were observed to fall short in fulfilling their roles as reference genes in the analysis of dynamic gene expression (data not shown). Therefore, a pool of candidate reference genes needs to be identified for dynamic studies. The most suitable set of reference genes would then be determined among the proposed candidates under the studied experimental conditions.

In this study, high-throughput transient gene expression data were used to identify a set of candidate reference genes for real-time RT-qPCR studies. The reference genes were selected from a large pool of dynamic microarray data sets such that they displayed stable expression profiles across time regardless of the type of experimental condition.

The initial stage of this study required the collection of publicly available time series microarray datasets. It was observed that the use of key words such as, “times series” or “time course” was insufficient to extract all the necessary information from the database. This fact signified the importance of human intervention in acquiring information from electronic sources. The content of the datasets needed to be carefully investigated by the researcher to evaluate whether the set was coherent with the context of the study or not. Only then a comprehensive set could be attained.

Two different approaches were used in the identification of a set of candidate reference genes. One approach utilized individual experimental data sets for the determination of stability rankings for each set. The frequency of occurrence of each gene among the most stable 100 genes was determined as a measure of selection criterion. The most frequently encountered genes across all experiments were identified as candidates. In the other approach, however, all available data were merged into a single complete dataset and the overall stability profile based on CV values was used as the second selection criterion. Three genes were identified by both approaches; *TDH3*, *TPI1* and *CDC19*. All three genes were determined as reference in at least either one of the two case studies.

The advantage of this present strategy for the identification of a pool of candidate reference gene sets is the flexibility of the method. It enables the implementation of other approaches in a modular manner. By this means, the candidate gene pool may be extended to meet other specific needs that might be required.

The approach, in which a combination of datasets were used to identify reference gene candidates through the calculation of CV values, showed that the number of the datasets used for the identification of stable genes affected the stability order of the genes. This result indicated the necessity to include as many datasets as possible in the analysis to obtain more reliable results. Thus as many time course experiments conducted using *S. cerevisiae* as possible were tried to be included in the present study. The strategy is not limited to the currently available datasets but allows the inclusion of additional data sets and this is advantageous in terms of improving the results obtained from this approach.

The stability analysis of the collected time series datasets revealed that the transcripts that take place in the super pathway of glucose fermentation (*FBA1*, *TPI1*, *PGK1*, *CDC19* and *PDC1*) tended to display stable expression profiles in time course studies. Furthermore, these transcripts were verified to display stable expression profiles experimentally thus presenting a good alternative as reference genes in the normalization of real-time RT-qPCR data. The dominance of fermentation–related genes as stable reference candidates in time course studies appears to be an interesting result, which requires further investigation.

**Figure 5:**
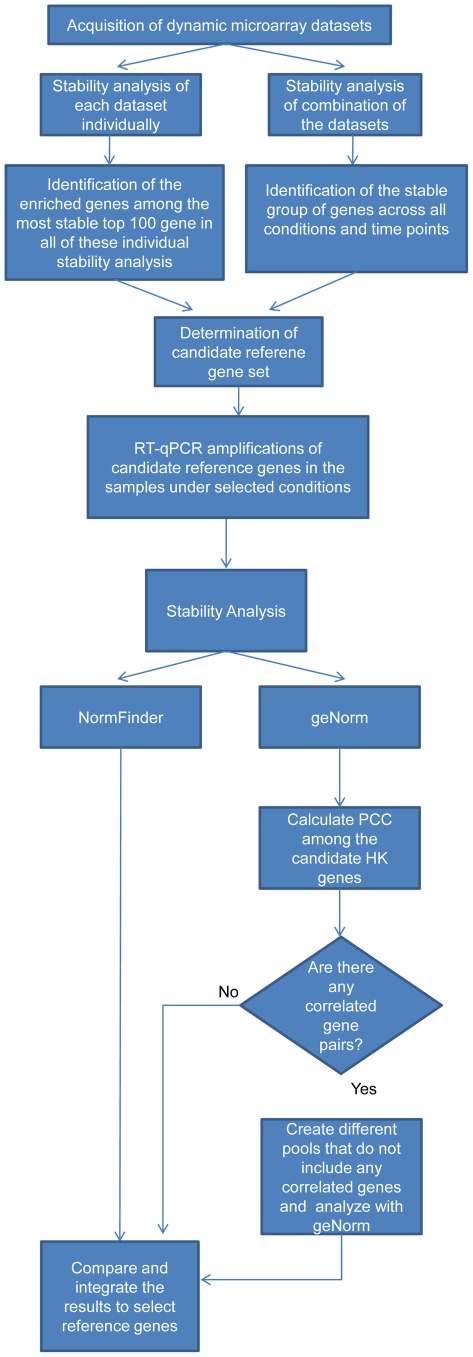
Flowchart representing the approach. This figure represents the general strategy followed for the identification of a pool of reference gene candidates and the determination of a reference gene set to be used for a specific condition in real-time RT-qPCR experiments.

In this study, geNorm and NormFinder algorithms were used for the identification of the most stable genes among the candidate list. For this purpose the results obtained from these two software programs were compared and the transcripts, which displayed the most stable expression profiles in both applications, were identified. The stability of the genes was evaluated based on a scoring system that allowed to display their average stability rankings. However, obtaining similar results using both software programs highly depended on the gene set to be analyzed since geNorm algorithm might be very sensitive to the existence of any correlated gene pairs. The results clearly showed that the exclusion of one of the correlated genes altered the stability order and the stability scores of the transcripts would be low only if the correlated genes were excluded from the candidate list. Our approach (Figure 5) is based on the elimination of the correlated gene pairs according to the results of the real-time RT-qPCR experiments under the selected conditions rather than the *a priori* elimination of genes that were reported to be correlated in the literature. This approach enabled the observation of any possible correlations among genes investigated in the samples of the current case studies and avoided the unnecessary exclusion of the candidates, which could possibly be among the most stable genes.

This study provided additional evidence that, there are no universal reference genes, which could suitably be used under different experimental conditions. Here we propose a candidate gene set, which can be used for the normalization of dynamic expression profiles of the target genes in *S. cerevisiae*. In order to confirm the suitability of this candidate gene set, we investigated the transcriptional profiles of *HAP4* and *MEP2* genes under two different experimental conditions using both the newly identified reference genes and the commonly used ones. These analyses clearly demonstrated that the newly identified reference genes outperformed the conventional candidates in dynamic expression profiling analysis.

The responses of *HAP4* and *MEP2* to an impulse-like addition of glucose and ammonium, respectively, were reported in several studies in the literature [17], [18]. Investigation of the expression profiles of genes with well-documented responses in an experimental condition was shown to aid the evaluation of the optimum number of reference genes selected from the candidate reference gene set. Therefore it would be suggested to conduct evaluations regarding the candidate reference gene set using such control genes with known expression profiles prior to conducting the analyses on the genes of interest.

The reference gene sets were shown to display stable expression profiles across time in both of the cases that were studied. Moreover these gene sets outperformed their individual members in terms of stability in time. The genes that were identified as the least stable both by NormFinder and geNorm were also experimentally shown to display variations in the expression profiles as well as 18S rRNA, which was also determined to be unsuitable as a reference gene under the stated conditions.

It should be noted that for each specific experimental condition that would be investigated, a different set of reference genes would selected from the candidate gene pool. The nature of the perturbation or the experimental setup would result in the identification of different reference genes for each specific experimental condition throughout the dynamic range of the experiment. In fact in Case Study I, in which the amount of glucose used as the sole carbon source was varied, *ACT1* was identified among the most stable reference genes although it alone was not sufficient for normalization. On the other hand, *ACT1* expression was shown to be unstable in another experimental setup for investigating diauxic shift, in which the cells starving for glucose switched to utilizing other carbon sources such as ethanol [4]. Yet another study utilized *ACT1* as reference for monitoring the expression levels of glycolytic genes in response to a switch from growing on ethanol to growing on glucose [6].

Several methodologies were used previously in the selection of RT-qPCR reference genes. One of the most commonly utilized strategies is the selection of one or more of the reference genes that are frequently cited in the literature [3], [5], [10], [20], [21], [33], [34], [35]. In rare instances, several different approaches were also utilized. In a study for validating reference genes for quantitative expression analysis by real-time RT-qPCR in *S. cerevisiae*, Teste *et al.* selected suitable microarray datasets, in which the culture conditions reported for these datasets were closest to their experimental setup. The potential reference genes were selected among the transcripts displaying stable expression profiles in these microarray datasets. Additionally traditionally used reference genes were also included in the study [4]. Another study focused on systematically collecting microarray data for selecting reference genes under a specific set of conditions [1]. In yet another study, the candidate reference genes were determined via stability of their expression levels across different tissues using their EST profiles [3]. Existence of a pool of commonly used reference genes has proven very useful in time invariant RT-qPCR analysis. However, these reference genes were often found to display far from stable expression profiles in dynamic studies mandating researchers to seek alternative ways to identify novel reference gene candidates [4]. Unfortunately currently utilized approaches usually fail in the identification of a universal set of candidate reference genes to be used in dynamic studies regardless of experimental conditions. Our approach may help serve this purpose by gathering dynamic microarray datasets having as diverse experimental conditions as possible for the identification of a universal pool of candidate reference genes, from which users may select subsets that would be suitable for their particular needs.

The present approach allows its users a vast space to manoeuvre using the proposed candidate reference genes. Although the specific cases presented in this study focused on transient changes in the amount of available glucose or ammonium in the fermentation medium in yeast, suitable reference genes would be selected among the candidate pool for completely different experimental conditions regardless of whether such an experimental setup was previously analyzed or not. The candidate pool itself was created from a collection of dynamic datasets with a diverse set of experimental conditions. Among these, environmental variations including osmotic stress, heat shock, cell cycle, DNA damage, nutrient availability, chemical treatments, desiccation stress, nitrosative stress and genetic mutations including deletion and overexpression of genes were included (Table S1). The genes in the candidate pool were identified such that regardless of the diversity of the experimental conditions under which the microarray data were generated, the expression profile of each candidate was stable. This strength of the method of selection increases the possibility of identifying reference gene among the pool of candidates, which would have stable expression profiles across time in a specific experimental condition that was not previously analyzed.

It can be concluded that the pool of candidate reference genes determined in this study; *TPI1*, *ACT1*, *TDH3*, *FBA1*, *CCW12*, *CDC19*, *ADH1*, *PGK1*, *GCN4*, *PDC1*, *RPS26A* and *ARF1*, may be used to identify the set of suitable reference genes in the analysis of dynamic transcriptional data by real-time RT-qPCR in *S. cerevisiae* under different experimental conditions. However, it is also undeniable that, as the number of the publicly available time course microarray datasets increases, this candidate gene set may be improved. The flexibility of the methods used in this approach enable the inclusion of additional datasets thus constant update of the candidate pool. Additional methods could also be implemented in a modular manner to enhance the results obtained from this study. This study showed the significance of researchers' intervention at various stages of reference gene selection both during the inclusion of new dynamic datasets to be used for the determination of the candidate set and during the exclusion of the correlated gene pairs from the candidate pool while identifying the most stable genes.

## Materials and Methods

### Determination of the reference gene candidates

In order to determine the reference candidates displaying stable expression throughout time course experiments, transcriptome data sets were used. Publicly available time series microarray data sets were downloaded from the Array Express database [12]. 31 different time-dependent experiments conducted with *S. cerevisiae* using Affymetrix Yeast Genome S98 and Yeast2 arrays (14 and 17 experiments, respectively) (Table S1) were first pre-processed with RMAExpress software applying quantile normalization [36]. For the multiple probe sets targeting the same gene, the average expression value would be used if there were no significant difference between the expression levels of the multiple probes and the larger value would be selected if there were a significant difference. The expression levels of the genes that were detected in both platforms were combined and a matrix of dimensions 5424×888 was obtained for the 5424 identified transcripts measured at 888 time points in 31 different experiments.

### Assessment of expression stability by coefficient of variation approach

The candidate reference genes with stable gene expression across time were determined by the coefficient of variation approach [2]. For each gene, the coefficient of variation (CV) of the expression value, which is defined as the ratio of the standard deviation to the mean was calculated and the CV values of the genes were then ranked.
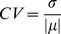
CV values were determined for the combined data set of 31 different experiments and also individually for each experiment. The CV values were used in the assessment of stable expression profiles and the candidate gene list was determined.

### Composition of the combined datasets

In order to determine the effect of inclusion of an additional dataset in the final cluster of experiments on the stability profile of the genes, combinations of 31 datasets were created. There were 465 possible combinations that contain 2 or 29 datasets and 31 possible combinations that contain 30 datasets. Stability profiles for each of these 496 combinations were identified. However, due to the excessive number of the possible combinations for the subsets containing 3 to 28 datasets, 1000 random combinations were generated for those subsets and the stability analysis was conducted for those random combinations.
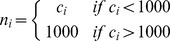



*n_i_*: number of generated subsets containing *i* datasets

*c_i_*: number of all possible subsets containing *i* datasets

where 1<*i*<31

### Confirmation of the stability of reference genes and their validation

#### Strain and media

Homozygous *ho*Δ*/ho*Δ strain of *S. cerevisiae* from a genetic background of BY4743 (*MAT*a*/MAT*Δ *his3*Δ*1*/*his3*Δ*1 leu2*Δ*0*/l*eu2*Δ*0 lys2*Δ*0*/+ *met15*Δ*0*/+ *ura3*Δ*0*/*ura3*Δ*0*), purchased from the European *Saccharomyces cerevisiae* Archive for Functional Analysis [37], was kindly provided by Prof. Stephen G. Oliver. The absence of the deleted gene was verified using PCR-based methods [14].

The preculture was incubated in YPD medium (2% [wt/vol] D-glucose, 2% [wt/vol] peptone, 1% [wt/vol] yeast extract) at 30°C and 180 rpm in an orbital shaker. Synthetic defined medium was used in the fermentations [38].

#### Chemostat culture, experimental design and sample description

Cells were cultivated in B-Braun Biostat B plus fermenters with 1.5 L working volume of ammonium or glucose-limited synthetic defined medium. Ammonium or glucose was injected in an impulse-like manner into their respective limited cultures at steady state. The temperature was controlled at 30°C and the agitation was set to 800 rpm. The pH was controlled at 5.5 with 1 M NaOH and HCl. The dilution rate was set to 0.1 hr^−1^. The air flow into the fermenters was adjusted at 1.5 L/min to keep dissolve oxygen saturation above 80% throughout the experiment. A total of 14 time-dependent RNA samples were collected at steady state, in the first minute with 20 second intervals, with 8 minute intervals for 32 minutes and then hourly for 5 hours following the impulse. The sample volume was 5 ml of culture at an OD range of 1.2–1.4. The samples were immediately frozen in liquid nitrogen and stored at −80°C until further processing.

#### Nucleic acid extraction

RNA was isolated from samples with the Qiagen RNeasy mini kit (Cat no: 74106) using the “RNeasy protocol for extracting yeast via enzymatic lysis” using robotic workstation, QIAcube (Qiagen, USA), modified by the manufacturer for yeast applications. β-mercaptoethanol (Cat no: 444203) was purchased from Merck. The nucleic acid concentrations were determined and the purity of the RNA (A_260_/A_280_) was confirmed using NanoDrop (ND-1000, Thermo Scientific) (Table S7). The observed yield was ensured to be at least 90% of the expected yield. RNA integrity was determined using Bioanalyzer 2100 (Agilent Technologies, USA) using RNA6000 Nanokit (Agilent Technologies, USA). RIN numbers and the electrophoresis traces were provided in Text S1. The absence of any DNA contamination was confirmed using RNase treated samples as negative control.

#### Reverse transcription

In order to unify the initial concentration of RNA in the analyses, all samples were diluted to the same concentration prior to the real-time RT-qPCR application. Reverse transcription was carried out at 50°C for 30 minutes. The reaction was allowed to proceed with 80 ng/µl in a reaction volume of 12.5 µl. QuantiTect RT mix (Qiagen, USA, Cat no: 204245) was used at a ratio of 0.01 total reaction volume. Assays were conducted in triplicates. The synthesized cDNA template was immediately allowed to proceed with the polymerase chain reaction.

#### qPCR target information

The PCR product (amplicon) was determined to be either 100–150 base pairs long or 200–250 base pairs long. The gene symbols, sequence accession numbers, location of the amplicon, amplicon lengths, *in silico* PCR results for specificity is provided in Text S2.

#### qPCR oligonucleotides

The primer length was selected to be in the range of 18–24 nucleotides. The GC content of the primers ranged between 50 to 60 per cent. The melting temperatures of the primers were in the range of 55–58°C. Primer3 software was used in the design of the primers except for that for 18S rRNA, which was designed in this study [39]. The complete set of primer pair sequences and RTPrimerDB identification numbers are listed in Table S2. The designed primers were manufactured by Alpha DNA (Montreal, Quebec) and desalted. The performance of all primers was experimentally confirmed by conventional PCR to ensure the amplification of a single region with the correct amplicon length.

#### qPCR protocol and qPCR validation

Qiagen QuantiTect® SYBR® Green one step RT-PCR kit was used for real-time RT-qPCR applications as described by the manufacturer (Qiagen, USA, Cat no: 204245). All kit contents are optimized and validated by the manufacturer. The PCR reactions were performed in a final reaction volume of 12.5 µl containing the final concentration of 2.5 mM of MgCl_2_ and 0.5 mM of forward and reverse primers. Plates and film sealers were manufactured from Bio-Rad Laboratories (Cat no: MSB1001, MLP9601). The reaction mixtures were prepared manually and the reactions were allowed to proceed in iCycler 5 instrument (Bio-Rad Laboratories). Assays were conducted in triplicates. The annealing temperature optimization and thermocycling parameters are provided in Table S8. RT-PCR sensitivity and reproducibility assays were conducted as described by the manufacturer (Qiagen, USA, Cat no: 204245). The melt curves indicating the specificity of the reaction were provided in Text S3. The range of the Cq values was determined as 10.9–31.70 for case study I and as 12.00–28.30 for case study II. The presence of no-template control wells were ensured in all reaction plates. Serial dilutions of RNA (5-point 4-fold dilution series, starting with 100 ng/reaction) were used for the measurement of the overall assay efficiency for one step RT-PCR. The log template amount was plotted against the corresponding Cq value and the slope (S) was determined. The efficiency (E) was calculated according to the following formula:
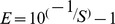
The efficiency values, standard curves and R^2^ of standard curves for each of the candidate reference genes and the target genes were provided in Table S2. The linear dynamic range of the Cq values was between 14.7 and 35.7. The 95% confidence interval at the lowest limit was determined to be less than 1 for the Cq values that were determined using either forward/reverse primer pair except for *ACT1* and *PDC1* (1.5 and 1.8, respectively). The average of the 95% confidence intervals of the Cq values throughout the range was 0.6.

#### Data analysis

iCycler™ iQ Optical System Software version 3.0a (Bio-Rad Laboratories) was used with PCR base line subtracted curve fit method for the measurement of quantification cycle (Cq). The geometric average of the Cq values with a standard deviation of less than 0.5 cycles was used in further calculations. Raw Cq values were used to determine the relative gene expression values (Q) using delta-Ct method [40]. Cq values for no-template control wells were excluded from further analysis since the values were greater than 35 or not detected. The raw Cq values and intra assay variations are provided as Table S9. The data analysis was carried out using Microsoft Office 2007 Excel implemented with the statistical analysis tool.

#### Assessment of expression stability by geNorm and NormFinder

The expression stability of candidate reference genes was evaluated using geNorm [5] and NormFinder [7] software programs. The expression values were provided as input for both algorithms. Delta-Ct method was used for the calculation of expression values with the gene having the lowest Cq value being used as the reference gene [5].

## Supporting Information

Figure S1 **Frequency distribution of transcripts in terms of their occurrence among top 100 most stable genes.** The stability ranking of all transcripts was determined in terms of CV values for each independent set of experiments. For every transcript, the number of datasets, in which that transcript occurred among the top 100 most stable genes, was determined. These numbers were then represented as a percentage of the total number of independent data sets. The percentage of data sets, in which a number of transcripts were represented, was plotted against that number of transcripts as a frequency distribution.
(TIF)

Figure S2 **Minimum and average PCC values as a function of the number of experiments.** PCC values were calculated in order to determine the correlation between the overall stability profile for 31 datasets and each stability profile, which was calculated from the combination of the changing number of datasets ranging between 1 and 30. For the case, in which a specific number of datasets were used, both the average PCC of the available dataset combinations and the minimum PCC were determined. The figure represents the variation in the average and the minimum PCC values as a function the number of the experiments, which were used in the calculation of the individual stability profiles that would be compared with the overall stability profile.
(TIF)

Figure S3 **A non-cumulative histogram plot of the number of genes assigned to a specific CV value.** The CV value of each transcript, which was calculated from the combination of all datasets, was used to obtain the overall stability profile. The CV values of the most stable 100 genes, which were determined based on this stability profile, were binned in a range such that the maximal and the minimal CV values were included. A non-cumulative histogram plot of this frequency distribution was presented in the figure.
(TIF)

Figure S4 **Stability analysis of the candidate genes in Case Study I.** The cells representing the stability ranking of the genes in different pools were indicated in the corresponding areas in a quantitative manner; 1 indicating the most stable gene and 11 indicating the least stable one. A dash was used to indicate the genes that were omitted from analysis due to having a correlated expression profile with another gene in the candidate set. geNorm was used for the stability analysis of the six different pools of candidate genes and the results were provided in the last six columns. The analysis was carried out using all genes (P-0), and using the 5 pools, each excluding a different set of correlated genes (P-1 to P-5). The stability analysis conducted using NormFinder with all candidate genes was provided in the first column.
(TIF)

Figure S5 **geNorm stability output charts for Case Study I.** The stability analysis based on the expression levels of the candidate genes obtained in Case Study I was conducted for each pool; P0 to P5 using geNorm software. The M values indicating the average expression stability for the candidate genes were provided in the output format that the software provided. The plots for P0 to P5 were represented in the figure as designated by the letters A to F, respectively.
(TIF)

Figure S6 **geNorm pairwise variation output charts for Case Study I.** The stability analysis based on the expression levels of the candidate genes obtained in Case Study I was conducted for each pool; P0 to P5 using geNorm software. The V values indicating the pairwise variation among consecutive candidate gene pairs were provided in the output format that the software provided. A cut-off threshold of 0.15 was used to determine the number of reference genes that was considered to be suitable by the geNorm algorithm. The plots for P0 to P5 were represented in the figure as designated by the letters A to F, respectively.
(TIF)

Figure S7 **Expression profile of** ***HAP4*** **using** ***ACT1*** **or** ***FBA1*** **-** ***CDC19*** **in response to the glucose impulse.** This figure represents the expression profile for *HAP4* using *ACT1* alone or *FBA1*- *CDC19* pair. The relative expression of *HAP4* was plotted against the time from the perturbation of the amount of glucose available in the medium. The relative expression of *HAP4* was determined using *ACT1* as the sole reference gene or the geometric average of the Ct values for *FBA1* and *CDC19*.
(TIF)

Figure S8 **Stability analysis of the candidate genes in Case Study II.** The cells representing the stability ranking of the genes in different pools were indicated in the corresponding areas in a quantitative manner; 1 indicating the most stable gene and 11 indicating the least stable one. A dash was used to indicate the genes that were omitted from analysis due to having a correlated expression profile with another gene in the candidate set. geNorm was used for the stability analysis of the four different pools of candidate genes and the results were provided in the last four columns. The analysis was carried out using all genes (P-0), and using the 3 pools, each excluding a different set of correlated genes (P-1 to P-3). The stability analysis conducted using NormFinder with all candidate genes was provided in the first column.
(TIF)

Figure S9 **geNorm pairwise variation output charts for Case Study II.** The stability analysis based on the expression levels of the candidate genes obtained in Case Study II was conducted for each pool; P0 to P5 using geNorm software. The V values indicating the pairwise variation among consecutive candidate gene pairs were provided in the output format that the software provided. A cut-off threshold of 0.15 was used to determine the number of reference genes that was considered to be suitable by the geNorm algorithm. The plots for P0 to P5 were represented in the figure as designated by the letters A to F, respectively.
(TIF)

Figure S10 **geNorm stability output charts for Case Study II.** The stability analysis based on the expression levels of the candidate genes obtained in Case Study II was conducted for each pool; P0 to P5 using geNorm software. The M values indicating the average expression stability for the candidate genes were provided in the output format that the software provided. The plots for P0 to P5 were represented in the figure as designated by the letters A to F, respectively.
(TIF)

Figure S11 **Expression profile of** ***MEP2*** **using** ***ACT1*** **-** ***TDH3*** **in response to the ammonium impulse.** This figure represents the expression profile for *MEP2* using *ACT1*-*TDH3* pair. The relative expression of *MEP2* was plotted against the time from the perturbation of the amount of ammonium available in the medium. The relative expression of *MEP2* was determined using the geometric average of the Ct values for *ACT1* and *TDH3*.
(TIF)

Figure S12 **Stability analysis by geNorm and NormFinder including 18S rRNA.** This figure represents the stability order of the candidate reference genes together with 18S rRNA using geNorm and NormFinder for Case Study I (A) and for Case Study II (B). For the geNorm analysis the M values indicating the average expression stability for the candidate genes were provided in the output format that the software provided. For the NormFinder analysis the stability values were provided in the tables listed from the most stable gene to the least stable gene.
(TIF)

Figure S13 **Raw Ct value profiles for the candidate reference genes.** This figure represents the raw Ct value profiles for the most and the least stable genes, for the reference gene set and for 18S rRNA for Case Study I (A) and for Case Study II (B). The Ct values were plotted against the sample numbers representing the sampling times (A). The most stable genes in Case Study I were *TPI1* and *CDC19* and the least stable gene was *ADH1*. The reference gene set was comprised of *TPI1*, *FBA1*, *CDC19* and *ACT1* (B). The most stable genes in Case Study II were *FBA1* and *TDH3* and the least stable gene was *RPS26A*. The reference gene set was comprised of *FBA1*, *TDH3*, *ACT1* and *CCW12*.
(TIF)

Figure S14 **Log. expression profiles for the candidate reference genes.** This figure represents the expression profiles, in which the expression values were converted into logarithmic scale for the ease of visualization, for the most and the least stable genes, for the reference gene set and for 18S rRNA for Case Study I (A) and for Case Study II (B). The log. converted expression profiles were plotted against the sample numbers representing the sampling times (A). The most stable genes in Case Study I were *TPI1* and *CDC19* and the least stable gene was *ADH1*. The reference gene set was comprised of *TPI1*, *FBA1*, *CDC19* and *ACT1* (B). The most stable genes in Case Study II were *FBA1* and *TDH3* and the least stable gene was *RPS26A*. The reference gene set was comprised of *FBA1*, *TDH3*, *ACT1* and *CCW12*.
(TIF)

Table S1 Dynamic microarray datasets used in this study.
(XLS)

Table S2 Gene primer sequences and efficiencies used in this study.
(XLS)

Table S3 MIQE checklist.
(XLS)

Table S4 PCCs between the expressions of candidate genes pairs in response to glucose pulse.
(XLS)

Table S5 PCCs between the expressions of candidate gene pairs in response to ammonium pulse.
(XLS)

Table S6 Pairwise variations between the (n+1)^th^ and the n^th^ genes for different pools of candidate sets.
(XLS)

Table S7 Nucleic acid quantification of the samples.
(XLS)

Table S8 Complete thermocycling and annealing temperature optimization parameters.
(XLS)

Table S9 Raw Cq values and intra-assay variations.
(XLS)

Text S1 Bioanalyzer reports of samples used in this study.
(DOC)

Text S2 Gene symbols, sequence accession numbers, amplicon location and lengths, *in silico* PCR results.
(DOC)

Text S3 Results of the melt curve analysis.
(DOC)
