# Supplementary figures and images for: A Novel Strategy for Selection and Validation of Reference Genes in Dynamic Multidimensional Experimental Design in Yeast
Source: PLoS One. 2012 Jun 4;7(6):e38351. doi: 10.1371/journal.pone.0038351 (PMC3366934; doi:10.1371/journal.pone.0038351)

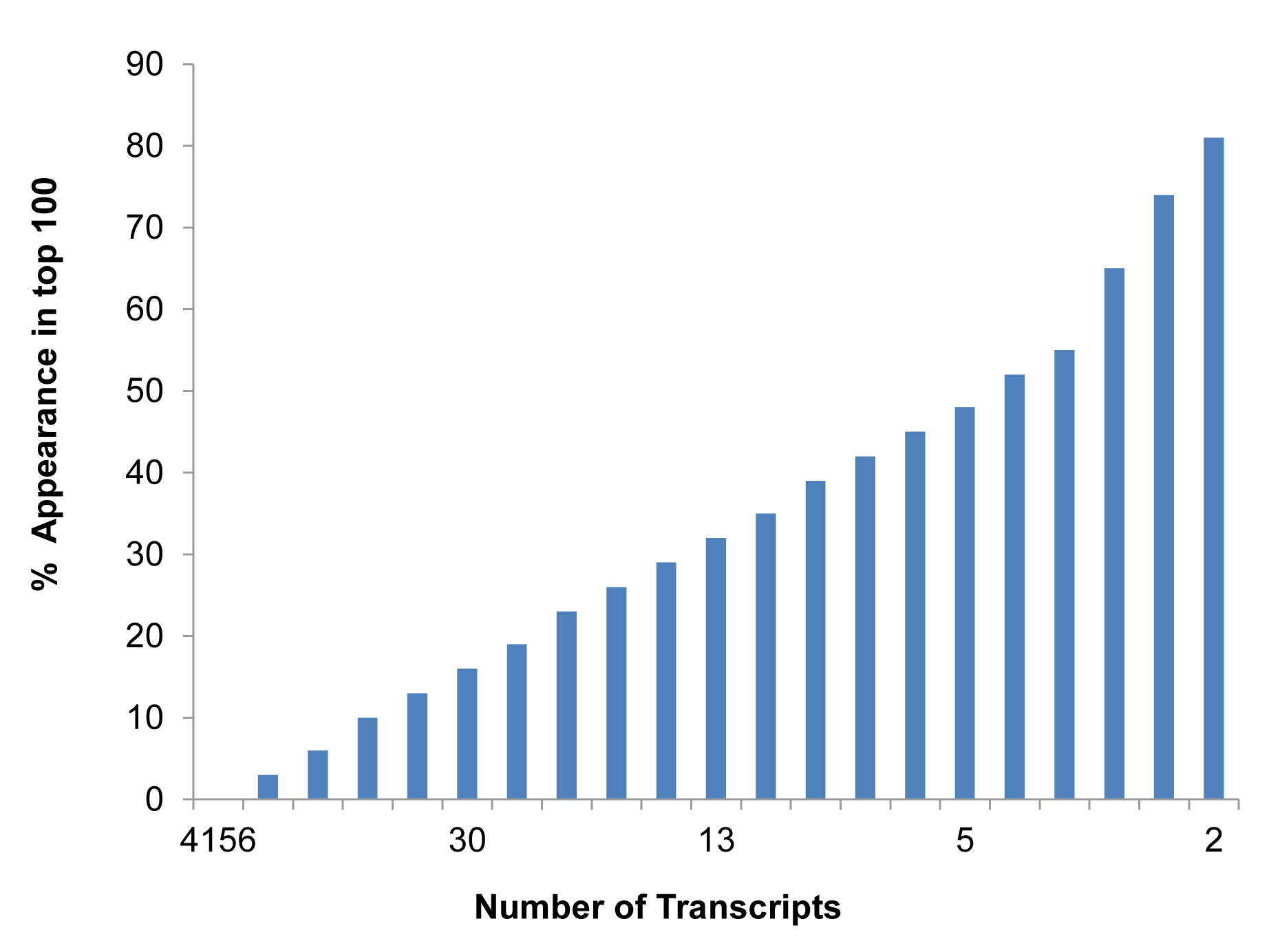

Supplement: Figure S1 — Frequency distribution of transcripts in terms of their occurrence among top 100 most stable genes. The stability ranking of all transcripts was determined in terms of CV values for each independent set of experiments. For every transcript, the number of datasets, in which that transcript occurred among the top 100 most stable genes, was determined. These numbers were then represented as a percentage of the total number of independent data sets. The percentage of data sets, in which a number of transcripts were represented, was plotted against that number of transcripts as a frequency distribution. (TIF) [file pone.0038351.s001.tif]

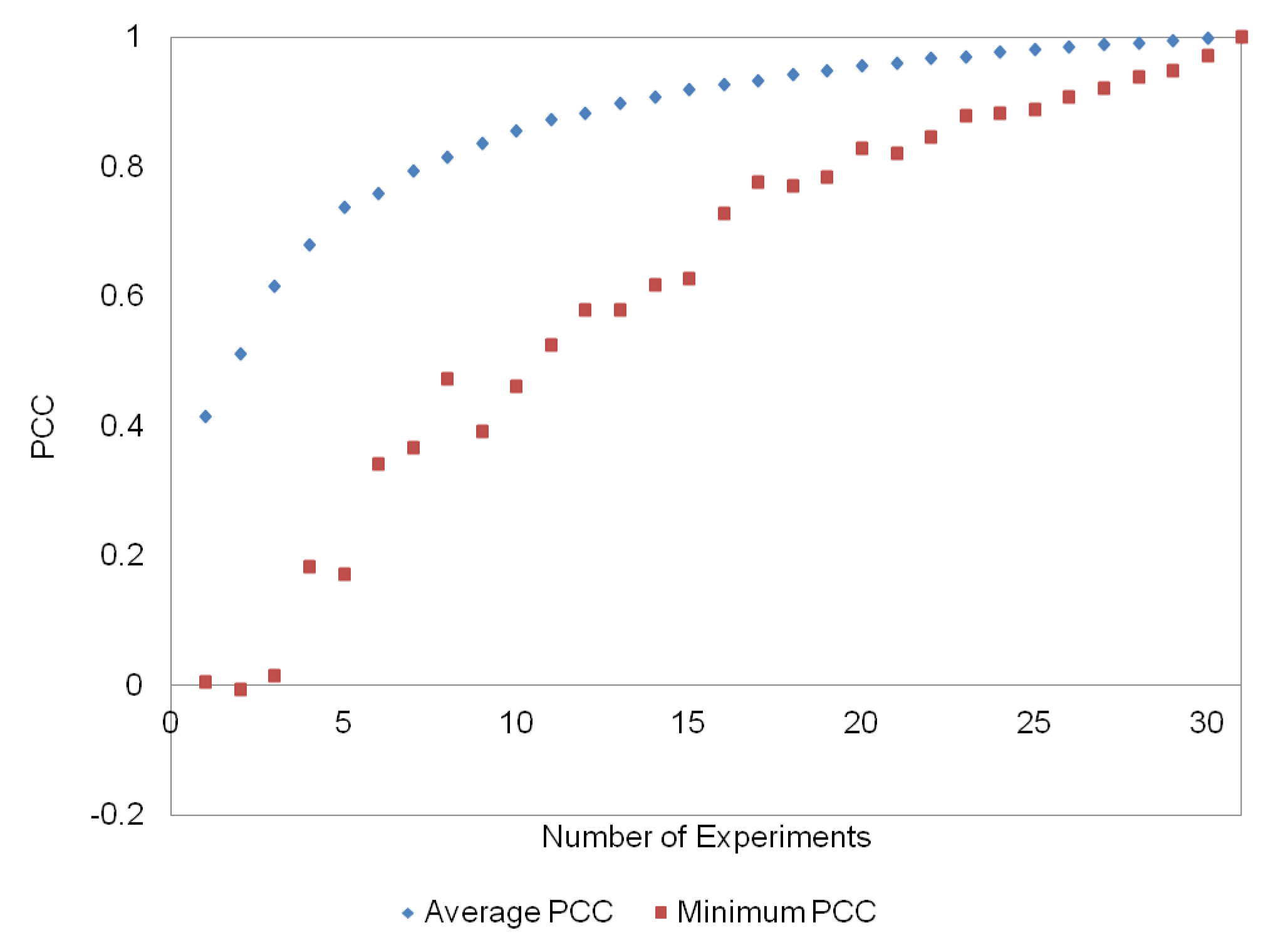

Supplement: Figure S2 — Minimum and average PCC values as a function of the number of experiments. PCC values were calculated in order to determine the correlation between the overall stability profile for 31 datasets and each stability profile, which was calculated from the combination of the changing number of datasets ranging between 1 and 30. For the case, in which a specific number of datasets were used, both the average PCC of the available dataset combinations and the minimum PCC were determined. The figure represents the variation in the average and the minimum PCC values as a function the number of the experiments, which were used in the calculation of the individual stability profiles that would be compared with the overall stability profile. (TIF) [file pone.0038351.s002.tif]

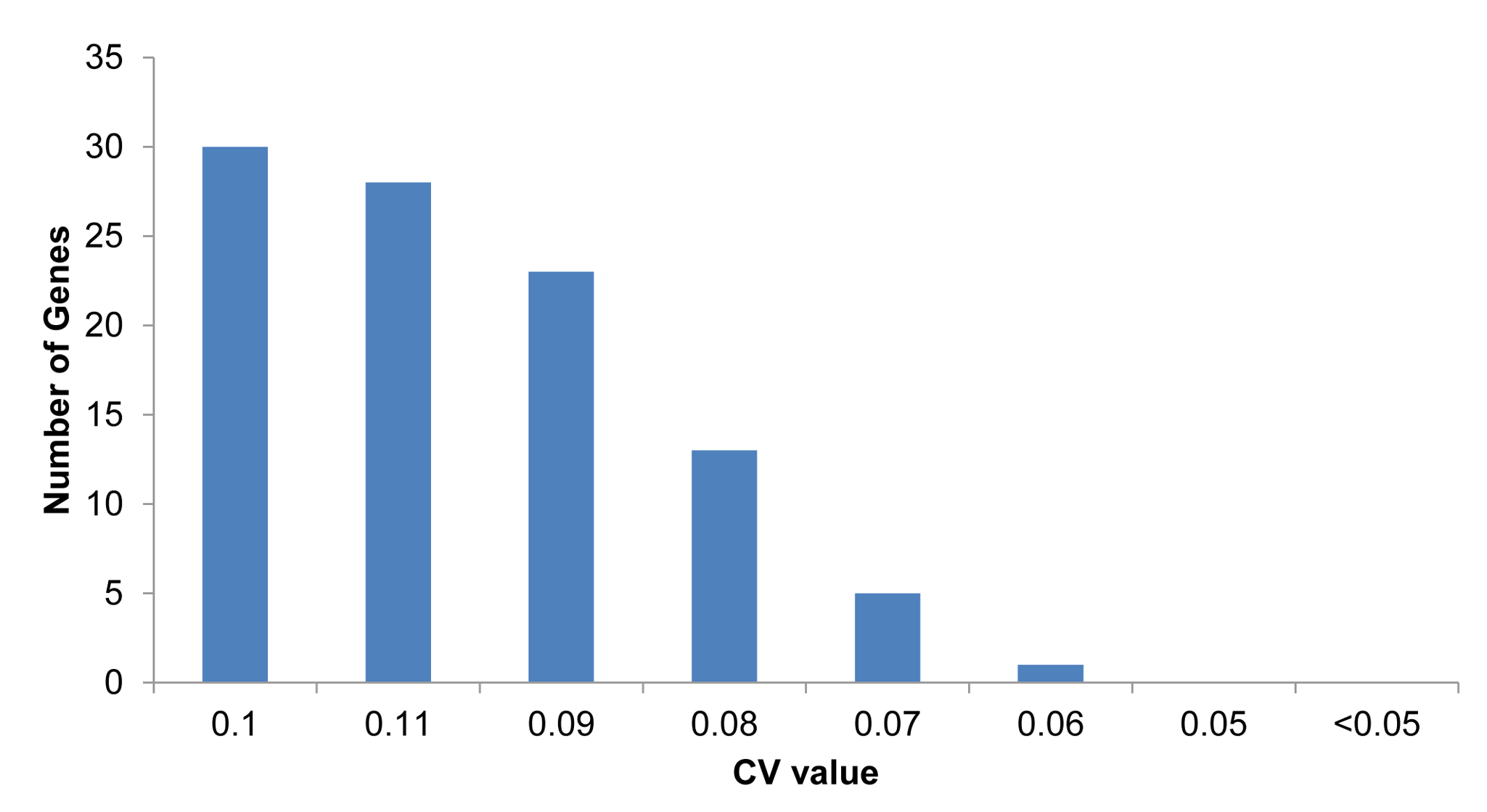

Supplement: Figure S3 — A non-cumulative histogram plot of the number of genes assigned to a specific CV value. The CV value of each transcript, which was calculated from the combination of all datasets, was used to obtain the overall stability profile. The CV values of the most stable 100 genes, which were determined based on this stability profile, were binned in a range such that the maximal and the minimal CV values were included. A non-cumulative histogram plot of this frequency distribution was presented in the figure. (TIF) [file pone.0038351.s003.tif]

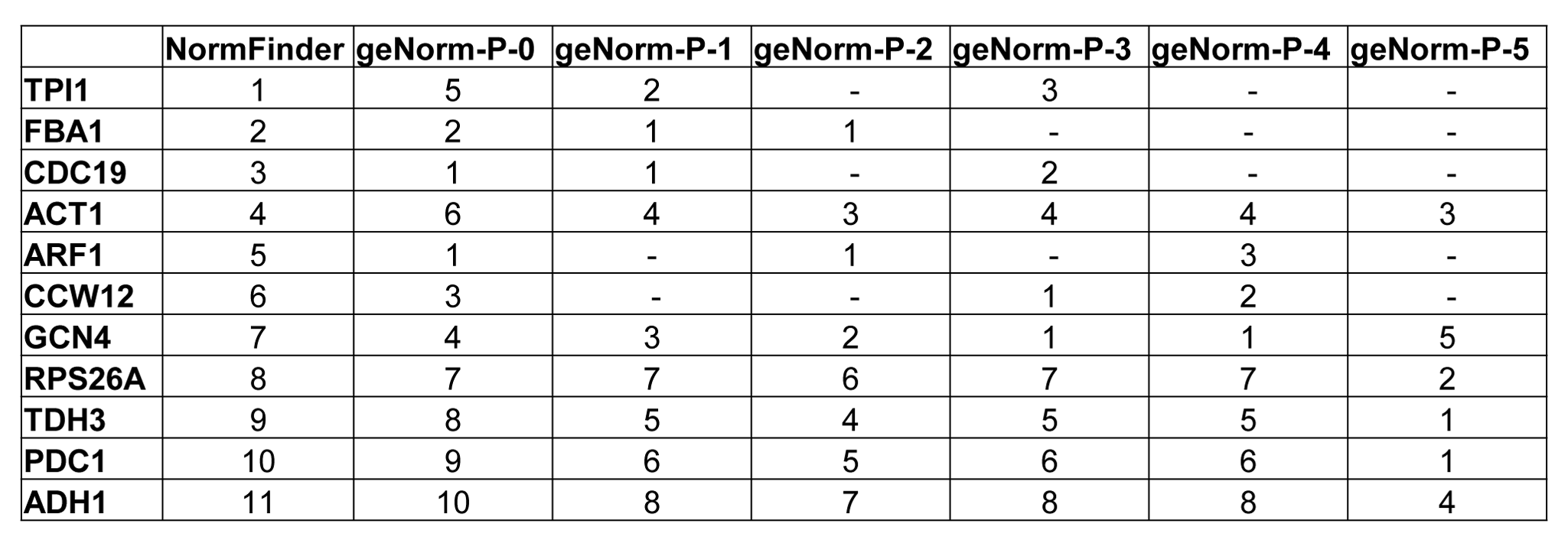

Supplement: Figure S4 — Stability analysis of the candidate genes in Case Study I. The cells representing the stability ranking of the genes in different pools were indicated in the corresponding areas in a quantitative manner; 1 indicating the most stable gene and 11 indicating the least stable one. A dash was used to indicate the genes that were omitted from analysis due to having a correlated expression profile with another gene in the candidate set. geNorm was used for the stability analysis of the six different pools of candidate genes and the results were provided in the last six columns. The analysis was carried out using all genes (P-0), and using the 5 pools, each excluding a different set of correlated genes (P-1 to P-5). The stability analysis conducted using NormFinder with all candidate genes was provided in the first column. (TIF) [file pone.0038351.s004.tif]

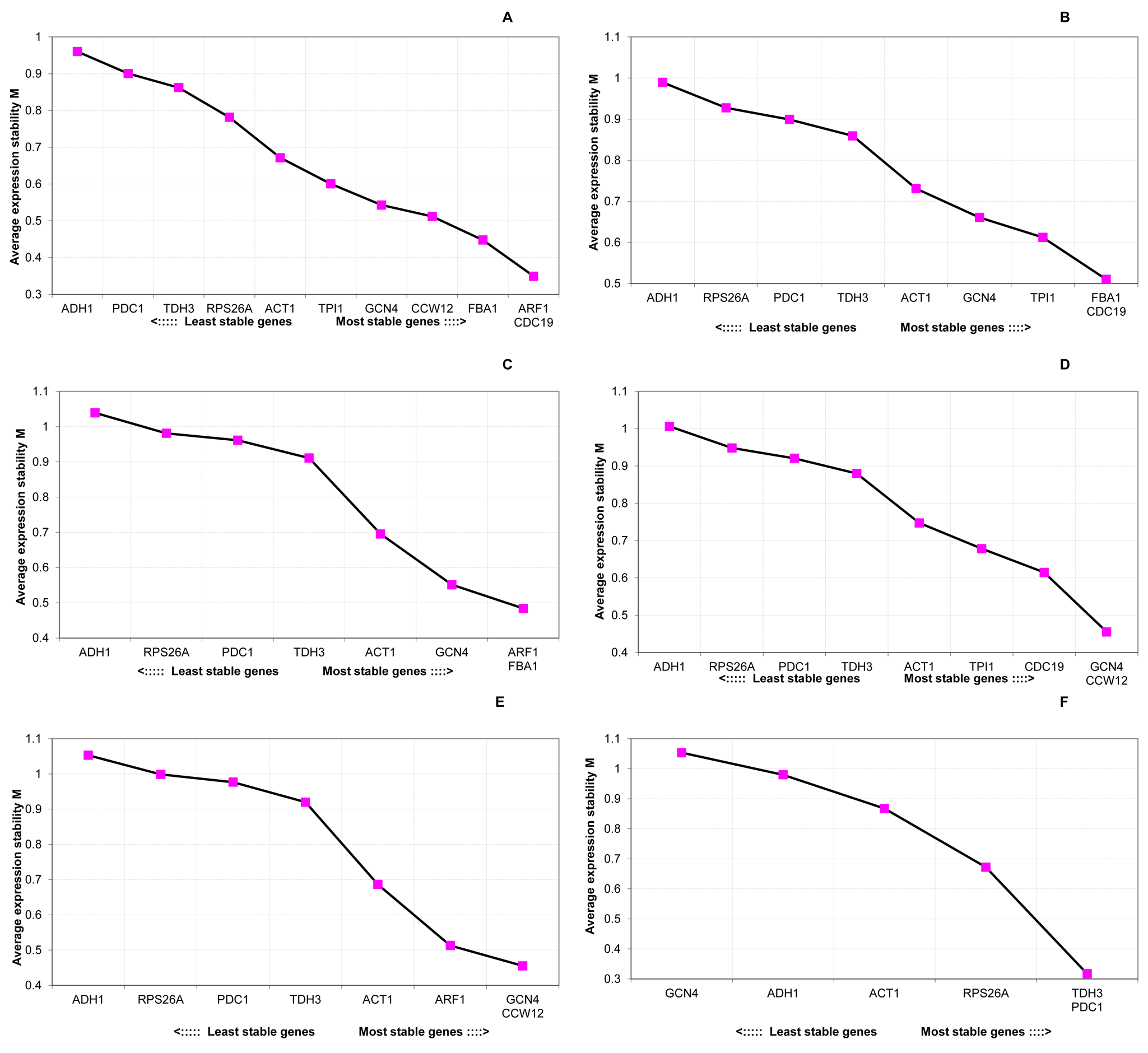

Supplement: Figure S5 — geNorm stability output charts for Case Study I. The stability analysis based on the expression levels of the candidate genes obtained in Case Study I was conducted for each pool; P0 to P5 using geNorm software. The M values indicating the average expression stability for the candidate genes were provided in the output format that the software provided. The plots for P0 to P5 were represented in the figure as designated by the letters A to F, respectively. (TIF) [file pone.0038351.s005.tif]

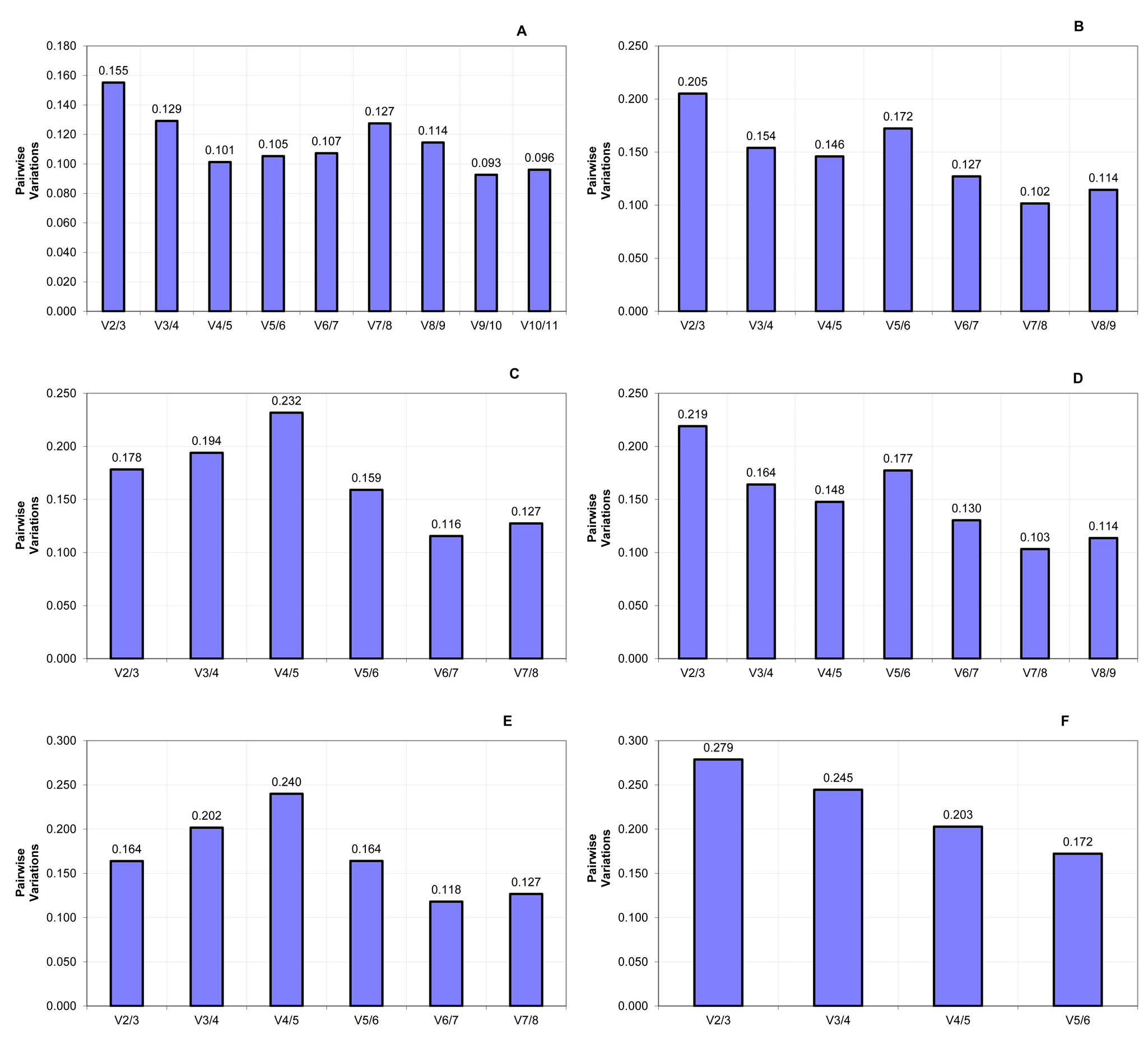

Supplement: Figure S6 — geNorm pairwise variation output charts for Case Study I. The stability analysis based on the expression levels of the candidate genes obtained in Case Study I was conducted for each pool; P0 to P5 using geNorm software. The V values indicating the pairwise variation among consecutive candidate gene pairs were provided in the output format that the software provided. A cut-off threshold of 0.15 was used to determine the number of reference genes that was considered to be suitable by the geNorm algorithm. The plots for P0 to P5 were represented in the figure as designated by the letters A to F, respectively. (TIF) [file pone.0038351.s006.tif]

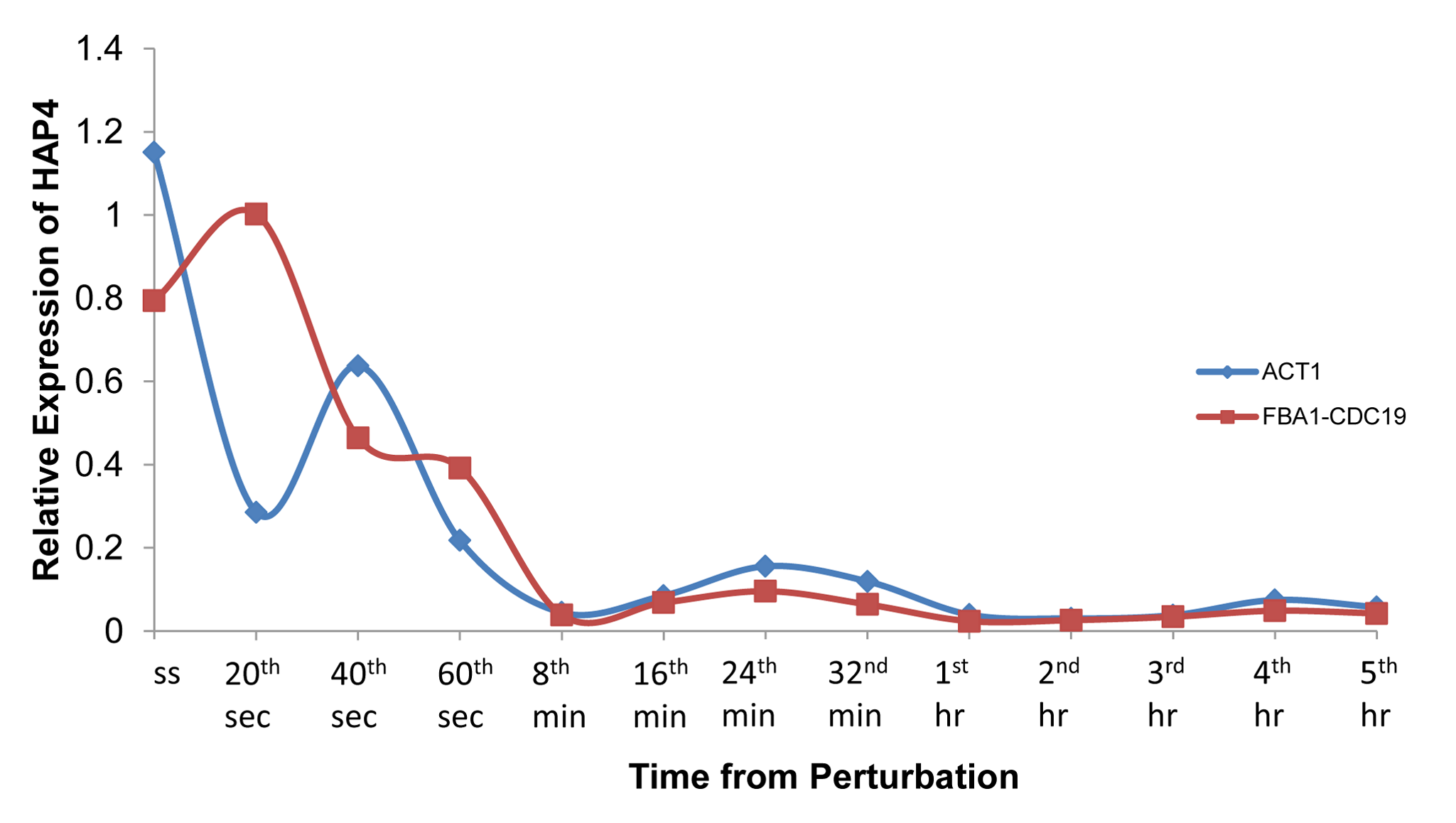

Supplement: Figure S7 — Expression profile of HAP4 using ACT1 or FBA1 - CDC19 in response to the glucose impulse. This figure represents the expression profile for HAP4 using ACT1 alone or FBA1- CDC19 pair. The relative expression of HAP4 was plotted against the time from the perturbation of the amount of glucose available in the medium. The relative expression of HAP4 was determined using ACT1 as the sole reference gene or the geometric average of the Ct values for FBA1 and CDC19. (TIF) [file pone.0038351.s007.tif]

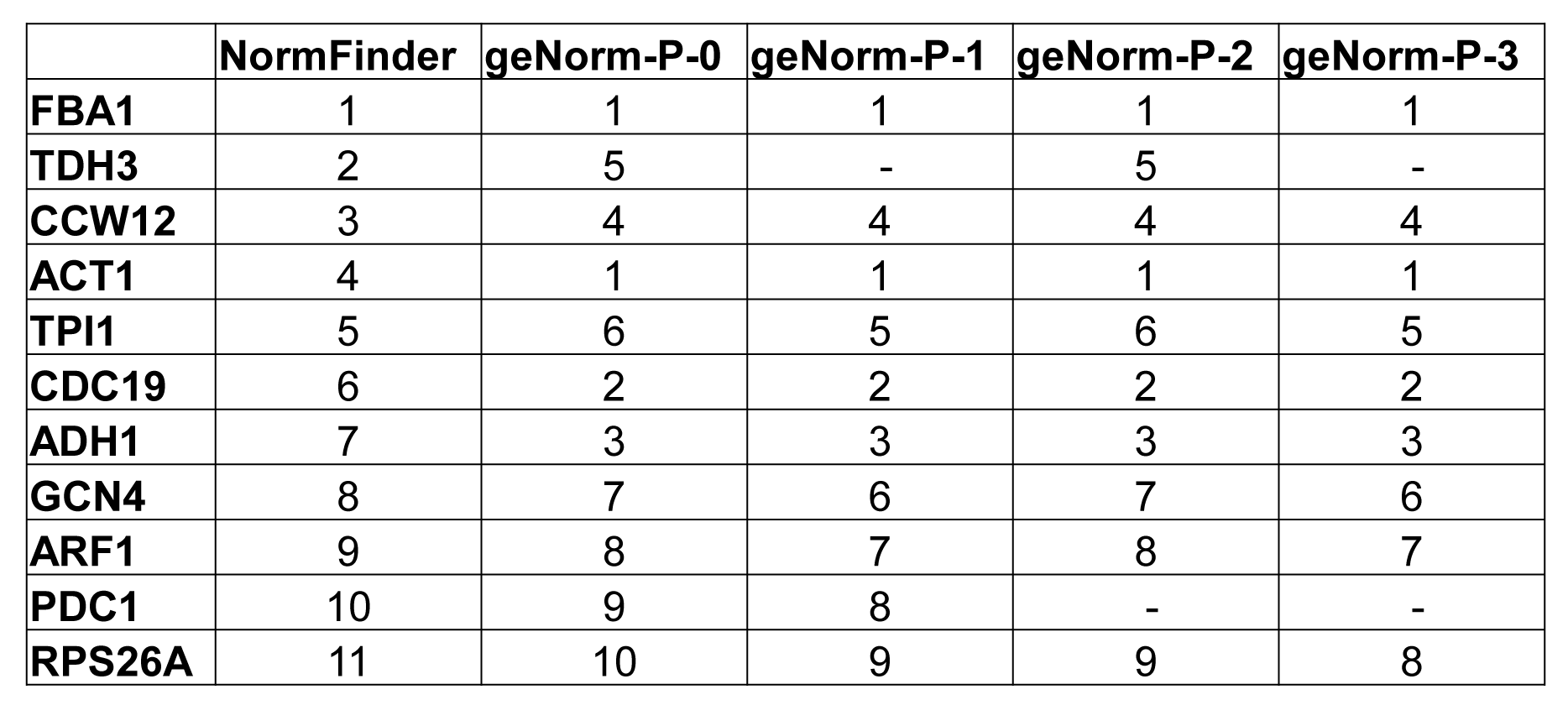

Supplement: Figure S8 — Stability analysis of the candidate genes in Case Study II. The cells representing the stability ranking of the genes in different pools were indicated in the corresponding areas in a quantitative manner; 1 indicating the most stable gene and 11 indicating the least stable one. A dash was used to indicate the genes that were omitted from analysis due to having a correlated expression profile with another gene in the candidate set. geNorm was used for the stability analysis of the four different pools of candidate genes and the results were provided in the last four columns. The analysis was carried out using all genes (P-0), and using the 3 pools, each excluding a different set of correlated genes (P-1 to P-3). The stability analysis conducted using NormFinder with all candidate genes was provided in the first column. (TIF) [file pone.0038351.s008.tif]

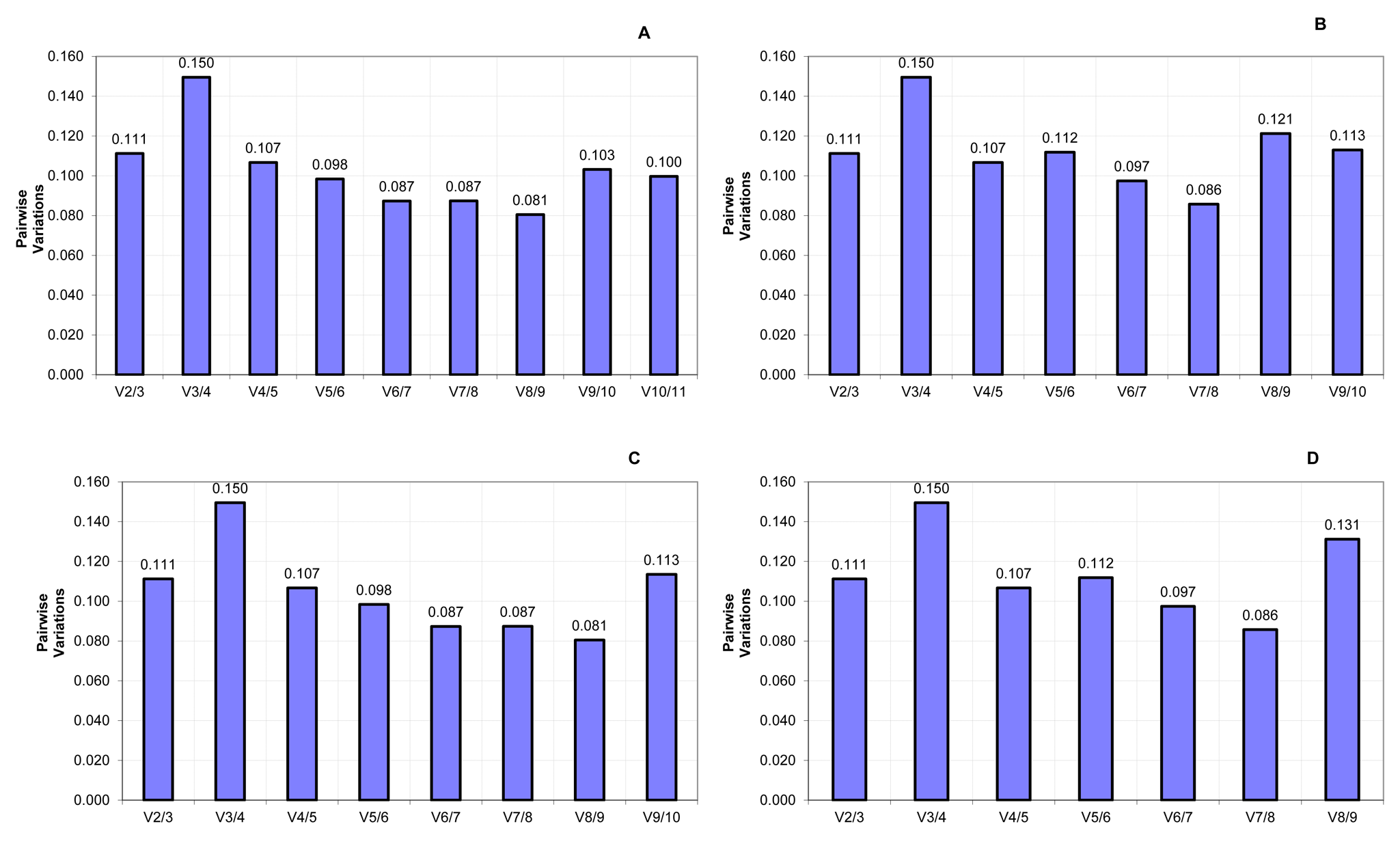

Supplement: Figure S9 — geNorm pairwise variation output charts for Case Study II. The stability analysis based on the expression levels of the candidate genes obtained in Case Study II was conducted for each pool; P0 to P5 using geNorm software. The V values indicating the pairwise variation among consecutive candidate gene pairs were provided in the output format that the software provided. A cut-off threshold of 0.15 was used to determine the number of reference genes that was considered to be suitable by the geNorm algorithm. The plots for P0 to P5 were represented in the figure as designated by the letters A to F, respectively. (TIF) [file pone.0038351.s009.tif]

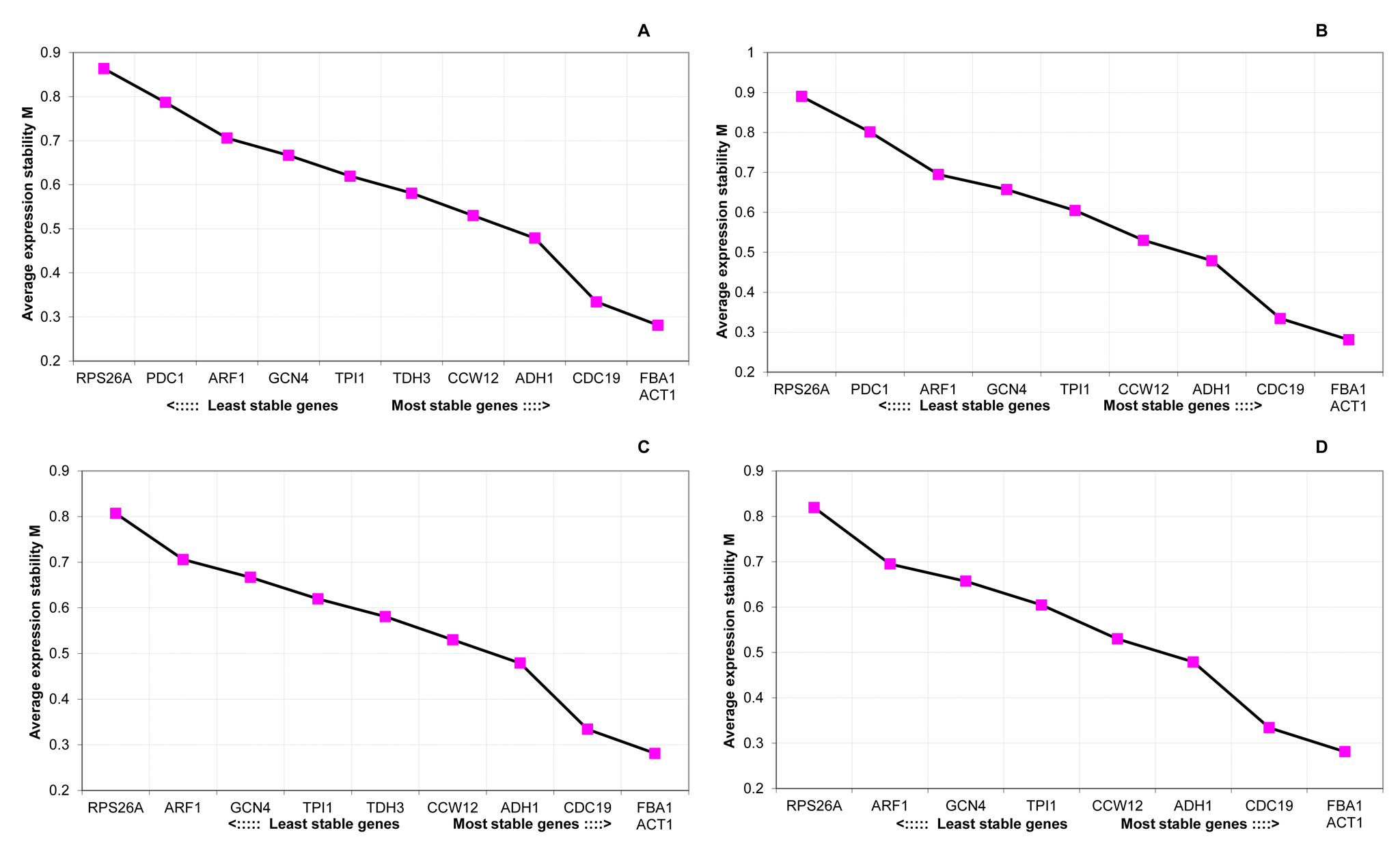

Supplement: Figure S10 — geNorm stability output charts for Case Study II. The stability analysis based on the expression levels of the candidate genes obtained in Case Study II was conducted for each pool; P0 to P5 using geNorm software. The M values indicating the average expression stability for the candidate genes were provided in the output format that the software provided. The plots for P0 to P5 were represented in the figure as designated by the letters A to F, respectively. (TIF) [file pone.0038351.s010.tif]

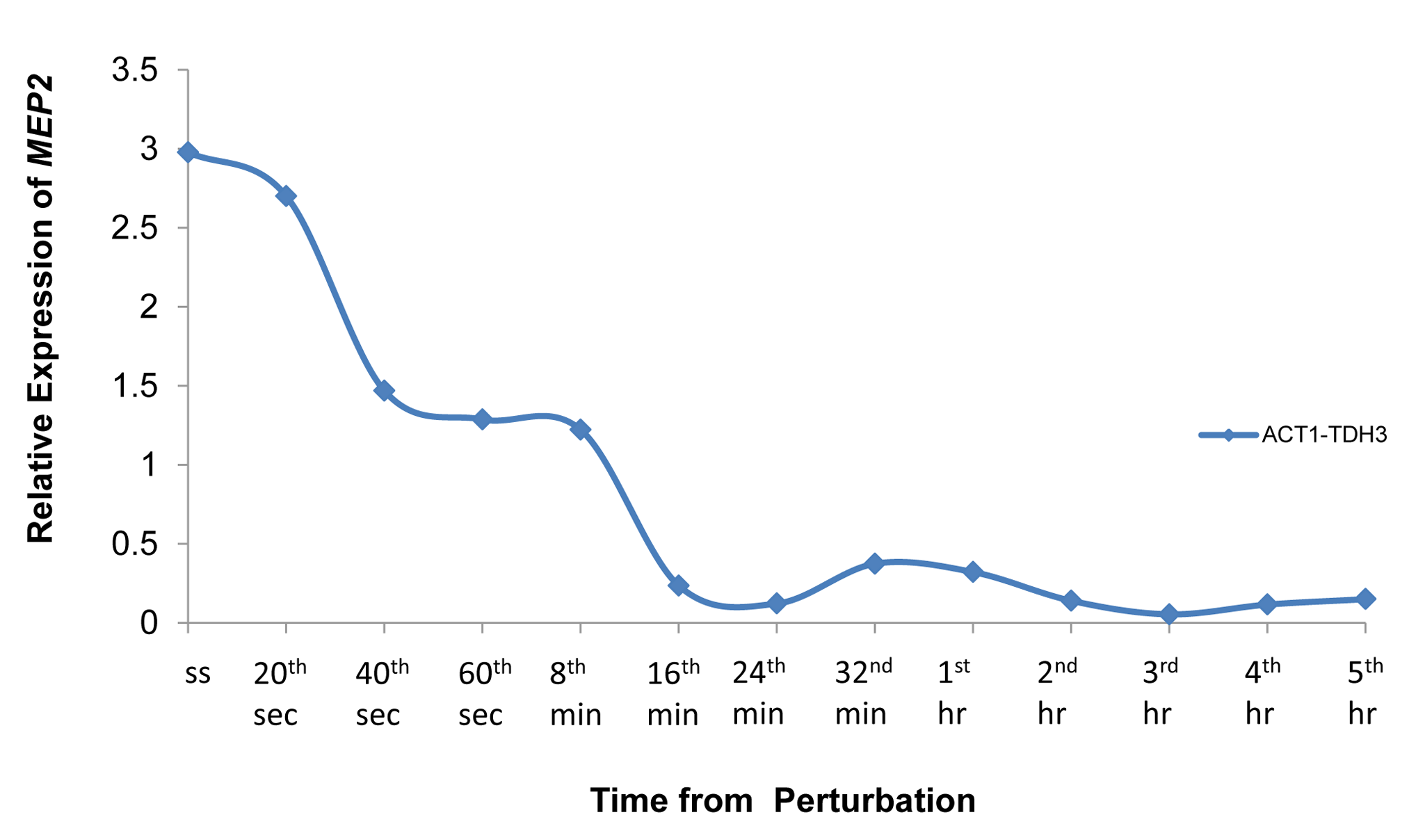

Supplement: Figure S11 — Expression profile of MEP2 using ACT1 - TDH3 in response to the ammonium impulse. This figure represents the expression profile for MEP2 using ACT1-TDH3 pair. The relative expression of MEP2 was plotted against the time from the perturbation of the amount of ammonium available in the medium. The relative expression of MEP2 was determined using the geometric average of the Ct values for ACT1 and TDH3. (TIF) [file pone.0038351.s011.tif]

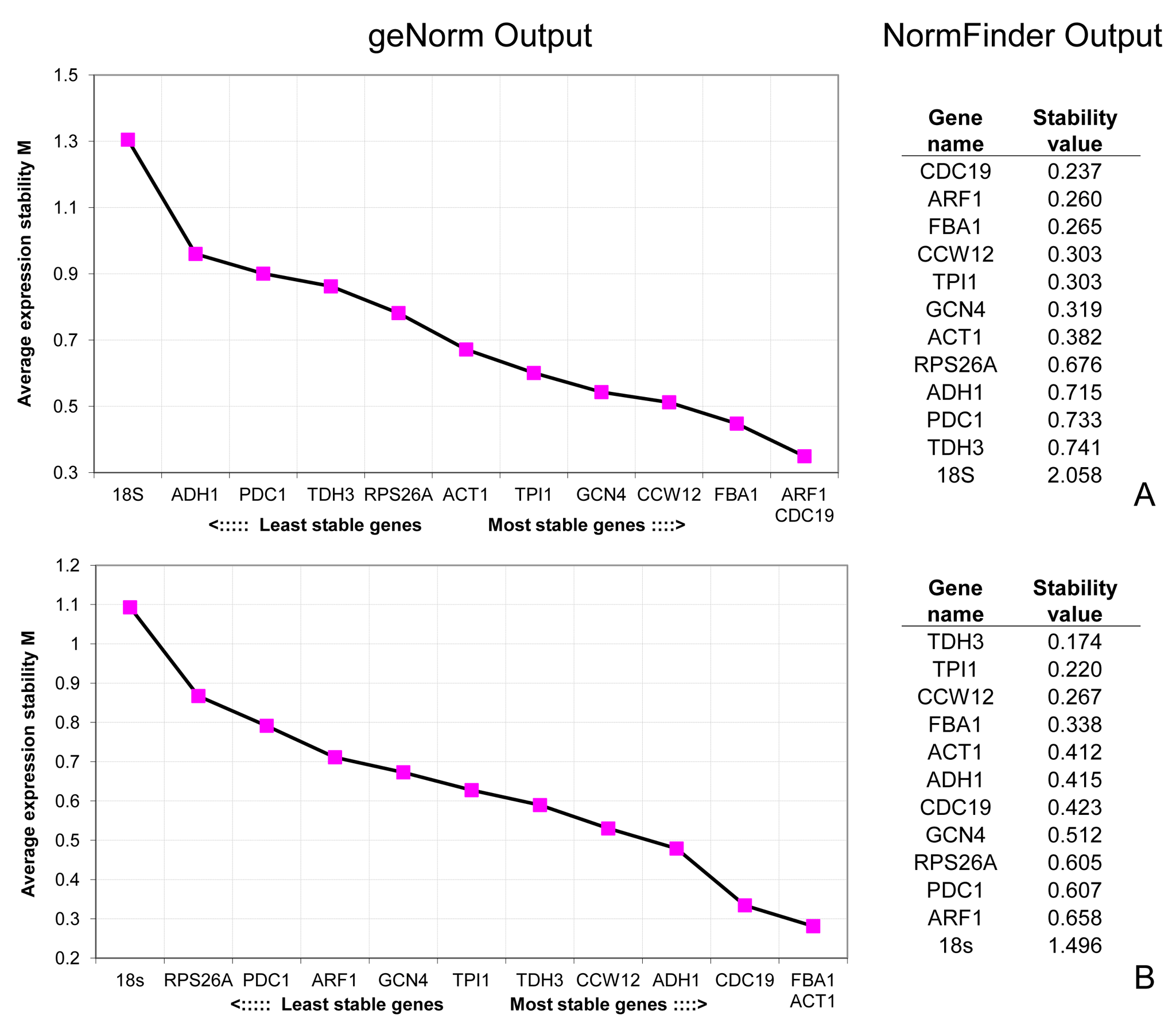

Supplement: Figure S12 — Stability analysis by geNorm and NormFinder including 18S rRNA. This figure represents the stability order of the candidate reference genes together with 18S rRNA using geNorm and NormFinder for Case Study I (A) and for Case Study II (B). For the geNorm analysis the M values indicating the average expression stability for the candidate genes were provided in the output format that the software provided. For the NormFinder analysis the stability values were provided in the tables listed from the most stable gene to the least stable gene. (TIF) [file pone.0038351.s012.tif]

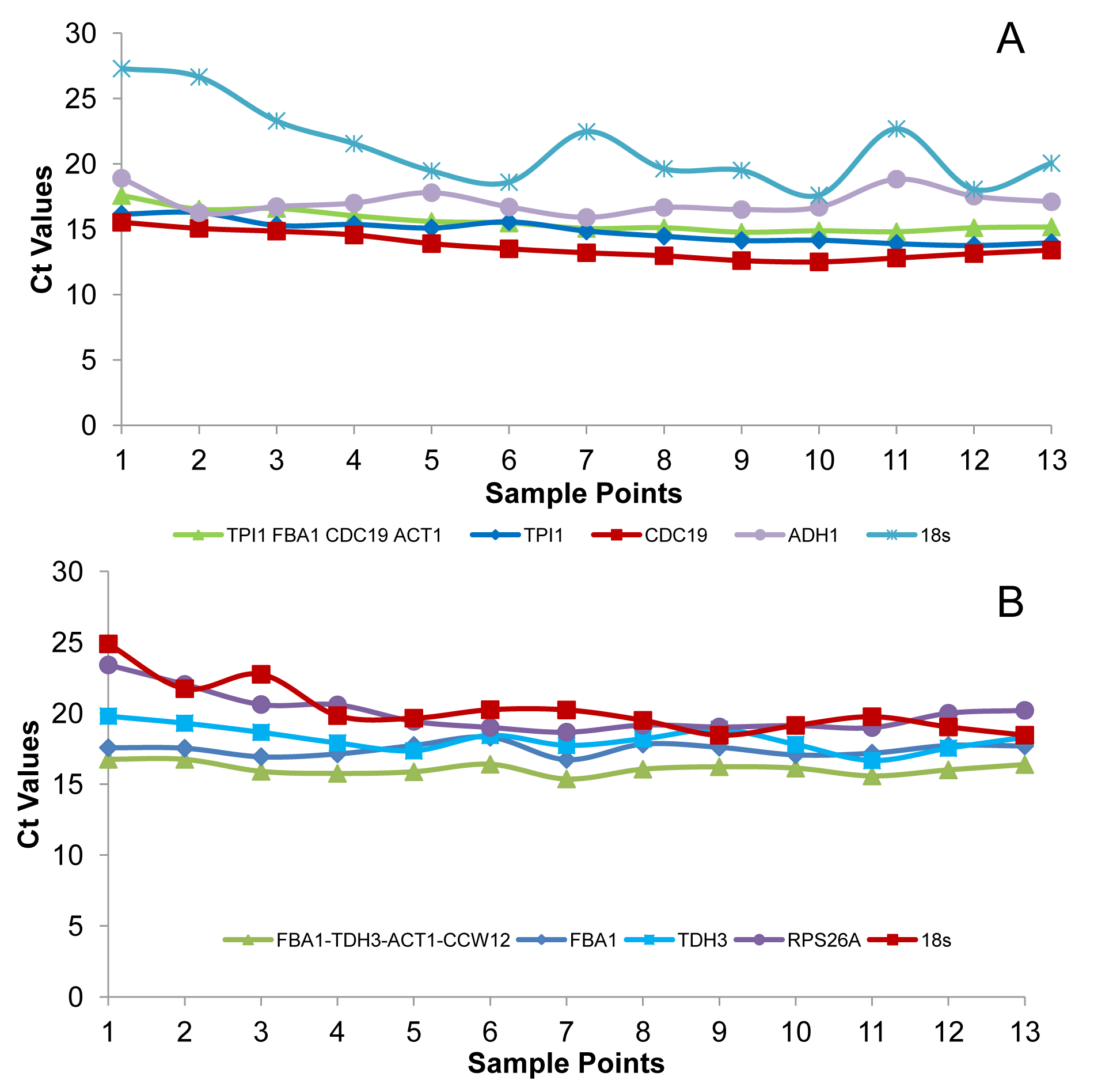

Supplement: Figure S13 — Raw Ct value profiles for the candidate reference genes. This figure represents the raw Ct value profiles for the most and the least stable genes, for the reference gene set and for 18S rRNA for Case Study I (A) and for Case Study II (B). The Ct values were plotted against the sample numbers representing the sampling times (A). The most stable genes in Case Study I were TPI1 and CDC19 and the least stable gene was ADH1. The reference gene set was comprised of TPI1, FBA1, CDC19 and ACT1 (B). The most stable genes in Case Study II were FBA1 and TDH3 and the least stable gene was RPS26A. The reference gene set was comprised of FBA1, TDH3, ACT1 and CCW12. (TIF) [file pone.0038351.s013.tif]

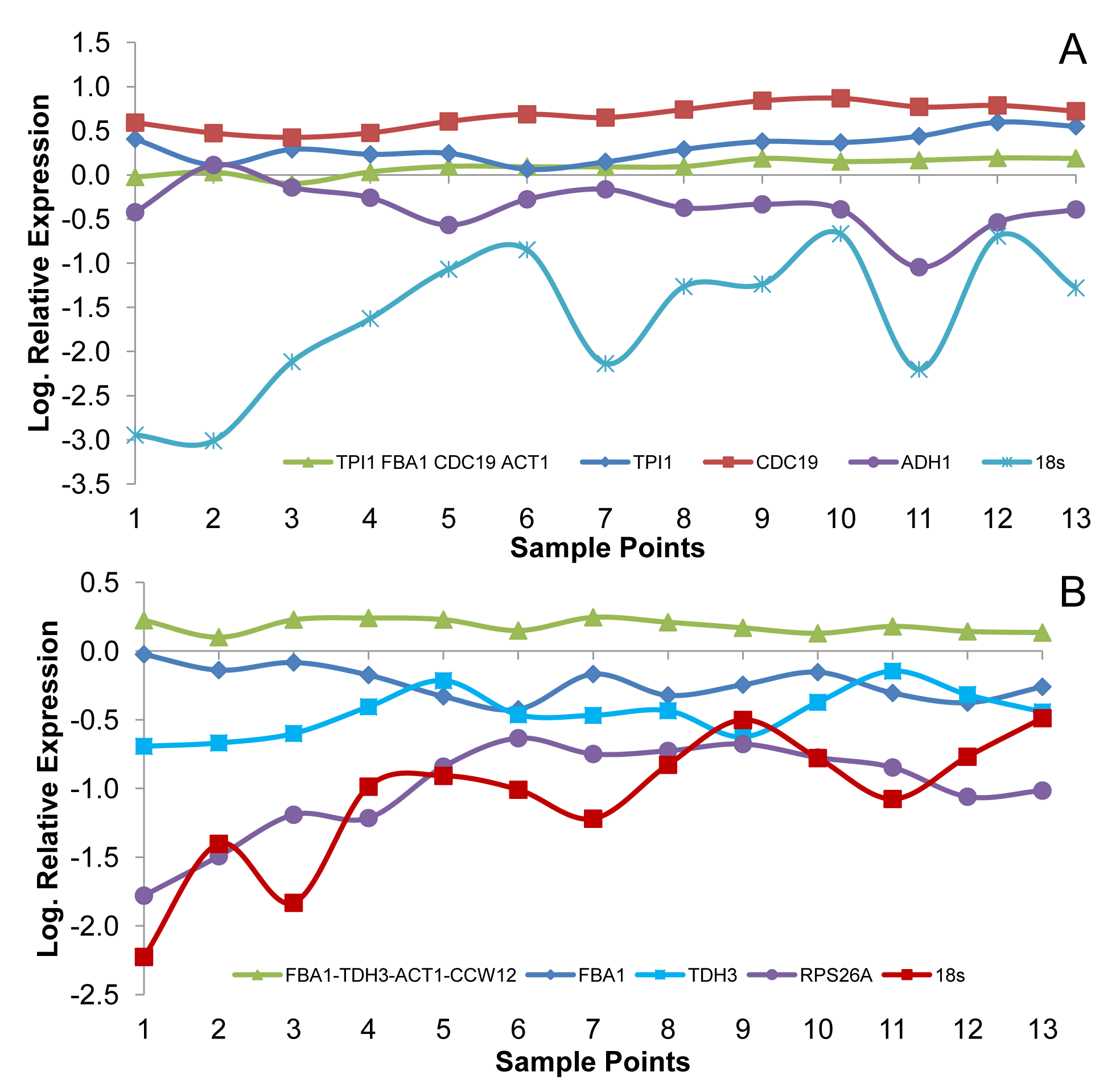

Supplement: Figure S14 — Log. expression profiles for the candidate reference genes. This figure represents the expression profiles, in which the expression values were converted into logarithmic scale for the ease of visualization, for the most and the least stable genes, for the reference gene set and for 18S rRNA for Case Study I (A) and for Case Study II (B). The log. converted expression profiles were plotted against the sample numbers representing the sampling times (A). The most stable genes in Case Study I were TPI1 and CDC19 and the least stable gene was ADH1. The reference gene set was comprised of TPI1, FBA1, CDC19 and ACT1 (B). The most stable genes in Case Study II were FBA1 and TDH3 and the least stable gene was RPS26A. The reference gene set was comprised of FBA1, TDH3, ACT1 and CCW12. (TIF) [file pone.0038351.s014.tif]
