# Supplementary material for: A Novel Strategy for Selection and Validation of Reference Genes in Dynamic Multidimensional Experimental Design in Yeast
Source: PLoS One. 2012 Jun 4;7(6):e38351. doi: 10.1371/journal.pone.0038351 (PMC3366934; doi:10.1371/journal.pone.0038351)
Supplement: Text S2 — Gene symbols, sequence accession numbers, amplicon location and lengths, in silico PCR results. (DOC) [file pone.0038351.s025.doc]

**1)YKL060C- FBA1 Chr 11**

**ACCESSION: NC_001143**

**GeneID: 853805**

ATGGGTGTTGAACAAATCTTAAAGAGAAAGACCGGTGTCATCGTTGGTGAAGATGTCCAC

AACTTATTCACTTACGCTAAGGAACACAAGTTCGCTATTCCAGCTATTAACGTCACCTCT

TCTTCTACTGCCGTCGCTGCTTTAGAAGCTGCTAGAGACAGCAAGTCCCCAATCATTTTG

CAAACCTCTAACGGTGGTGCTGCTTACTTCGCTGGTAAGGGTATCTCTAACGAAGGTCAA

AATGCTTCCATCAAGGGTGCTATTGCCGCTGCCCACTACATCAGATCCATTGCTCCAGCT

TACGGTATCCCAGTTGTCTTACACTCTGACCACTGTGCCAAGAAGTTGTTGCCATGGTTC

GATGGTATGTTGGAAGCTGATGAAGCTTACTTCAAGGAACACGGTGAACCATTATTCTCC

TCCCACATGTTGGATTTGTCTGAAGAAACCGATGAAGAAAACATCTCTACTTGTGTCAAG

TACTTCAAGAGAATGGCCGCTATGGACCAATGGTTAGAAATGGAAATCGGTATTACCGGT

GGTGAAGAAGATGGTGTTAACAACGAAAACGCTGACAAGGAAGACTTGTACACCAAGCCA

GAACAAGTTTACAACGTCTACAAGGCTTTGCACCCAATCTCTCCAAACTTCTCCATTGCT

GCTGCTTTCGGTAACTGTCACGGTTTGTACGCTGGTGACATCGCTTTGAGACCAGAAATC

TTGGCTGAACACCAAAAGTACACCAGAGAACAAGTTGGTTGCAAGGAAGAAAAGCCATTG

TTCTTGGTCTTCCACGGTGGTTCCGGTTCTACTGTCCAAGAATTCCACACTGGTATTGAC

AACGGTGTTGTCAAGGTCAACTTGGACACTGACTGTCAATACGCTTACTTGACTGGTATC

AGAGACTACGTCTTGAACAAGAAGGACTACATAATGTCCCCAGTCGGTAACCCAGAAGGT

CCAGAAAAGCCAAACAAGAAGTTCTTCGACCCAAGAGTCTGGGTTAGAGAAGGTGAAAAG

ACCATGGGTGCTAAGATCACCAAGTCTTTGGAAACTTTCCGTACCACTAACACTTTATAA

## Primer3 Output

No mispriming library specified

Using 1-based sequence positions

OLIGO [start](http://frodo.wi.mit.edu/primer3/primer3_www_results_help.html#PRIMER_START)  [len](http://frodo.wi.mit.edu/primer3/primer3_www_results_help.html#PRIMER_LEN)  [tm](http://frodo.wi.mit.edu/primer3/primer3_www_results_help.html#PRIMER_TM)  [gc%](http://frodo.wi.mit.edu/primer3/primer3_www_results_help.html#PRIMER_GC)  [any](http://frodo.wi.mit.edu/primer3/primer3_www_results_help.html#PRIMER_ANY)  [3'](http://frodo.wi.mit.edu/primer3/primer3_www_results_help.html#PRIMER_REPEAT) [seq](http://frodo.wi.mit.edu/primer3/primer3_www_results_help.html#PRIMER_OLIGO_SEQ)

LEFT PRIMER 583 20 56.28 50.00 6.00 0.00 GACTTGTACACCAAGCCAGA

RIGHT PRIMER 702 20 56.58 50.00 4.00 0.00 GATGTCACCAGCGTACAAAC

SEQUENCE SIZE: 1080

INCLUDED REGION SIZE: 1080

PRODUCT SIZE: 120, PAIR ANY COMPL: 6.00, PAIR 3' COMPL: 0.00

1 ATGGGTGTTGAACAAATCTTAAAGAGAAAGACCGGTGTCATCGTTGGTGAAGATGTCCAC

61 AACTTATTCACTTACGCTAAGGAACACAAGTTCGCTATTCCAGCTATTAACGTCACCTCT

121 TCTTCTACTGCCGTCGCTGCTTTAGAAGCTGCTAGAGACAGCAAGTCCCCAATCATTTTG

181 CAAACCTCTAACGGTGGTGCTGCTTACTTCGCTGGTAAGGGTATCTCTAACGAAGGTCAA

241 AATGCTTCCATCAAGGGTGCTATTGCCGCTGCCCACTACATCAGATCCATTGCTCCAGCT

301 TACGGTATCCCAGTTGTCTTACACTCTGACCACTGTGCCAAGAAGTTGTTGCCATGGTTC

361 GATGGTATGTTGGAAGCTGATGAAGCTTACTTCAAGGAACACGGTGAACCATTATTCTCC

421 TCCCACATGTTGGATTTGTCTGAAGAAACCGATGAAGAAAACATCTCTACTTGTGTCAAG

481 TACTTCAAGAGAATGGCCGCTATGGACCAATGGTTAGAAATGGAAATCGGTATTACCGGT

541 GGTGAAGAAGATGGTGTTAACAACGAAAACGCTGACAAGGAAGACTTGTACACCAAGCCA

>>>>>>>>>>>>>>>>>>

601 GAACAAGTTTACAACGTCTACAAGGCTTTGCACCCAATCTCTCCAAACTTCTCCATTGCT

>>

661 GCTGCTTTCGGTAACTGTCACGGTTTGTACGCTGGTGACATCGCTTTGAGACCAGAAATC

<<<<<<<<<<<<<<<<<<<<

721 TTGGCTGAACACCAAAAGTACACCAGAGAACAAGTTGGTTGCAAGGAAGAAAAGCCATTG

781 TTCTTGGTCTTCCACGGTGGTTCCGGTTCTACTGTCCAAGAATTCCACACTGGTATTGAC

841 AACGGTGTTGTCAAGGTCAACTTGGACACTGACTGTCAATACGCTTACTTGACTGGTATC

901 AGAGACTACGTCTTGAACAAGAAGGACTACATAATGTCCCCAGTCGGTAACCCAGAAGGT

961 CCAGAAAAGCCAAACAAGAAGTTCTTCGACCCAAGAGTCTGGGTTAGAGAAGGTGAAAAG

1021 ACCATGGGTGCTAAGATCACCAAGTCTTTGGAAACTTTCCGTACCACTAACACTTTATAA

UCSC In-Silico PCR

|  |  |
| --- | --- |
|  | >[chrXI:326786-326905](http://genome.ucsc.edu/cgi-bin/hgTracks?hgsid=219399449&db=sacCer3&position=chrXI:326786-326905&hgPcrResult=pack) 120bp GACTTGTACACCAAGCCAGA GATGTCACCAGCGTACAAAC  GACTTGTACACCAAGCCAGAacaagtttacaacgtctacaaggctttgca  cccaatctctccaaacttctccattgctgctgctttcggtaactgtcacg  GTTTGTACGCTGGTGACATC |

**2)YLR044C- PDC1-Chr 12**

**ACCESSION: NC_001144**

**GeneID:850733**

ATGTCTGAAATTACTTTGGGTAAATATTTGTTCGAAAGATTAAAGCAAGTCAACGTTAAC

ACCGTTTTCGGTTTGCCAGGTGACTTCAACTTGTCCTTGTTGGACAAGATCTACGAAGTT

GAAGGTATGAGATGGGCTGGTAACGCCAACGAATTGAACGCTGCTTACGCCGCTGATGGT

TACGCTCGTATCAAGGGTATGTCTTGTATCATCACCACCTTCGGTGTCGGTGAATTGTCT

GCTTTGAACGGTATTGCCGGTTCTTACGCTGAACACGTCGGTGTTTTGCACGTTGTTGGT

GTCCCATCCATCTCTGCTCAAGCTAAGCAATTGTTGTTGCACCACACCTTGGGTAACGGT

GACTTCACTGTTTTCCACAGAATGTCTGCCAACATTTCTGAAACCACTGCTATGATCACT

GACATTGCTACCGCCCCAGCTGAAATTGACAGATGTATCAGAACCACTTACGTCACCCAA

AGACCAGTCTACTTAGGTTTGCCAGCTAACTTGGTCGACTTGAACGTCCCAGCTAAGTTG

TTGCAAACTCCAATTGACATGTCTTTGAAGCCAAACGATGCTGAATCCGAAAAGGAAGTC

ATTGACACCATCTTGGCTTTGGTCAAGGATGCTAAGAACCCAGTTATCTTGGCTGATGCT

TGTTGTTCCAGACACGACGTCAAGGCTGAAACTAAGAAGTTGATTGACTTGACTCAATTC

CCAGCTTTCGTCACCCCAATGGGTAAGGGTTCCATTGACGAACAACACCCAAGATACGGT

GGTGTTTACGTCGGTACCTTGTCCAAGCCAGAAGTTAAGGAAGCCGTTGAATCTGCTGAC

TTGATTTTGTCTGTCGGTGCTTTGTTGTCTGATTTCAACACCGGTTCTTTCTCTTACTCT

TACAAGACCAAGAACATTGTCGAATTCCACTCCGACCACATGAAGATCAGAAACGCCACT

TTCCCAGGTGTCCAAATGAAATTCGTTTTGCAAAAGTTGTTGACCACTATTGCTGACGCC

GCTAAGGGTTACAAGCCAGTTGCTGTCCCAGCTAGAACTCCAGCTAACGCTGCTGTCCCA

GCTTCTACCCCATTGAAGCAAGAATGGATGTGGAACCAATTGGGTAACTTCTTGCAAGAA

GGTGATGTTGTCATTGCTGAAACCGGTACCTCCGCTTTCGGTATCAACCAAACCACTTTC

CCAAACAACACCTACGGTATCTCTCAAGTCTTATGGGGTTCCATTGGTTTCACCACTGGT

GCTACCTTGGGTGCTGCTTTCGCTGCTGAAGAAATTGATCCAAAGAAGAGAGTTATCTTA

TTCATTGGTGACGGTTCTTTGCAATTGACTGTTCAAGAAATCTCCACCATGATCAGATGG

GGCTTGAAGCCATACTTGTTCGTCTTGAACAACGATGGTTACACCATTGAAAAGTTGATT

CACGGTCCAAAGGCTCAATACAACGAAATTCAAGGTTGGGACCACCTATCCTTGTTGCCA

ACTTTCGGTGCTAAGGACTATGAAACCCACAGAGTCGCTACCACCGGTGAATGGGACAAG

TTGACCCAAGACAAGTCTTTCAACGACAACTCTAAGATCAGAATGATTGAAATCATGTTG

CCAGTCTTCGATGCTCCACAAAACTTGGTTGAACAAGCTAAGTTGACTGCTGCTACCAAC

GCTAAGCAATAA

## Primer3 Output

No mispriming library specified

Using 1-based sequence positions

OLIGO [start](http://frodo.wi.mit.edu/primer3/primer3_www_results_help.html#PRIMER_START)  [len](http://frodo.wi.mit.edu/primer3/primer3_www_results_help.html#PRIMER_LEN)  [tm](http://frodo.wi.mit.edu/primer3/primer3_www_results_help.html#PRIMER_TM)  [gc%](http://frodo.wi.mit.edu/primer3/primer3_www_results_help.html#PRIMER_GC)  [any](http://frodo.wi.mit.edu/primer3/primer3_www_results_help.html#PRIMER_ANY)  [3'](http://frodo.wi.mit.edu/primer3/primer3_www_results_help.html#PRIMER_REPEAT) [seq](http://frodo.wi.mit.edu/primer3/primer3_www_results_help.html#PRIMER_OLIGO_SEQ)

LEFT PRIMER 183 20 57.73 50.00 2.00 0.00 CGCTCGTATCAAGGGTATGT

RIGHT PRIMER 327 20 55.45 50.00 4.00 0.00 CTTAGCTTGAGCAGAGATGG

SEQUENCE SIZE: 1692

INCLUDED REGION SIZE: 1692

PRODUCT SIZE: 145, PAIR ANY COMPL: 5.00, PAIR 3' COMPL: 0.00

1 ATGTCTGAAATTACTTTGGGTAAATATTTGTTCGAAAGATTAAAGCAAGTCAACGTTAAC

61 ACCGTTTTCGGTTTGCCAGGTGACTTCAACTTGTCCTTGTTGGACAAGATCTACGAAGTT

121 GAAGGTATGAGATGGGCTGGTAACGCCAACGAATTGAACGCTGCTTACGCCGCTGATGGT

181 TACGCTCGTATCAAGGGTATGTCTTGTATCATCACCACCTTCGGTGTCGGTGAATTGTCT

>>>>>>>>>>>>>>>>>>>>

241 GCTTTGAACGGTATTGCCGGTTCTTACGCTGAACACGTCGGTGTTTTGCACGTTGTTGGT

301 GTCCCATCCATCTCTGCTCAAGCTAAGCAATTGTTGTTGCACCACACCTTGGGTAACGGT

<<<<<<<<<<<<<<<<<<<<

361 GACTTCACTGTTTTCCACAGAATGTCTGCCAACATTTCTGAAACCACTGCTATGATCACT

421 GACATTGCTACCGCCCCAGCTGAAATTGACAGATGTATCAGAACCACTTACGTCACCCAA

481 AGACCAGTCTACTTAGGTTTGCCAGCTAACTTGGTCGACTTGAACGTCCCAGCTAAGTTG

541 TTGCAAACTCCAATTGACATGTCTTTGAAGCCAAACGATGCTGAATCCGAAAAGGAAGTC

601 ATTGACACCATCTTGGCTTTGGTCAAGGATGCTAAGAACCCAGTTATCTTGGCTGATGCT

661 TGTTGTTCCAGACACGACGTCAAGGCTGAAACTAAGAAGTTGATTGACTTGACTCAATTC

721 CCAGCTTTCGTCACCCCAATGGGTAAGGGTTCCATTGACGAACAACACCCAAGATACGGT

781 GGTGTTTACGTCGGTACCTTGTCCAAGCCAGAAGTTAAGGAAGCCGTTGAATCTGCTGAC

841 TTGATTTTGTCTGTCGGTGCTTTGTTGTCTGATTTCAACACCGGTTCTTTCTCTTACTCT

901 TACAAGACCAAGAACATTGTCGAATTCCACTCCGACCACATGAAGATCAGAAACGCCACT

961 TTCCCAGGTGTCCAAATGAAATTCGTTTTGCAAAAGTTGTTGACCACTATTGCTGACGCC

1021 GCTAAGGGTTACAAGCCAGTTGCTGTCCCAGCTAGAACTCCAGCTAACGCTGCTGTCCCA

1081 GCTTCTACCCCATTGAAGCAAGAATGGATGTGGAACCAATTGGGTAACTTCTTGCAAGAA

1141 GGTGATGTTGTCATTGCTGAAACCGGTACCTCCGCTTTCGGTATCAACCAAACCACTTTC

1201 CCAAACAACACCTACGGTATCTCTCAAGTCTTATGGGGTTCCATTGGTTTCACCACTGGT

1261 GCTACCTTGGGTGCTGCTTTCGCTGCTGAAGAAATTGATCCAAAGAAGAGAGTTATCTTA

1321 TTCATTGGTGACGGTTCTTTGCAATTGACTGTTCAAGAAATCTCCACCATGATCAGATGG

1381 GGCTTGAAGCCATACTTGTTCGTCTTGAACAACGATGGTTACACCATTGAAAAGTTGATT

1441 CACGGTCCAAAGGCTCAATACAACGAAATTCAAGGTTGGGACCACCTATCCTTGTTGCCA

1501 ACTTTCGGTGCTAAGGACTATGAAACCCACAGAGTCGCTACCACCGGTGAATGGGACAAG

1561 TTGACCCAAGACAAGTCTTTCAACGACAACTCTAAGATCAGAATGATTGAAATCATGTTG

1621 CCAGTCTTCGATGCTCCACAAAACTTGGTTGAACAAGCTAAGTTGACTGCTGCTACCAAC

1681 GCTAAGCAATAA

| | UCSC In-Silico PCR   |  |  | | | --- | --- | --- | |  | >[chrXII:233755-233899](http://genome.ucsc.edu/cgi-bin/hgTracks?hgsid=219399449&db=sacCer3&position=chrXII:233755-233899&hgPcrResult=pack) 145bp CGCTCGTATCAAGGGTATGT CTTAGCTTGAGCAGAGATGG  CGCTCGTATCAAGGGTATGTcttgtatcatcaccaccttcggtgtcggtg  aattgtctgctttgaacggtattgccggttcttacgctgaacacgtcggt  gttttgcacgttgttggtgtcccatCCATCTCTGCTCAAGCTAAG |  | | | --- | --- | --- | --- | --- | --- | --- | |
| --- | --- | --- | --- | --- | --- | --- | --- |

**3) YEL009C-GCN4-Chr 5**

**ACCESSION: NC_001137**

**GeneID:856709**

ATGTCCGAATATCAGCCAAGTTTATTTGCTTTAAATCCAATGGGTTTCTCACCATTGGAT

GGTTCTAAATCAACCAACGAAAATGTATCTGCTTCCACTTCTACTGCCAAACCAATGGTT

GGCCAATTGATTTTTGATAAATTCATCAAGACTGAAGAGGATCCAATTATCAAACAGGAT

ACCCCTTCGAACCTTGATTTTGATTTTGCTCTTCCACAAACGGCAACTGCACCTGATGCC

AAGACCGTTTTGCCAATTCCGGAGCTAGATGACGCTGTAGTGGAATCTTTCTTTTCGTCA

AGCACTGATTCAACTCCAATGTTTGAGTATGAAAACCTAGAAGACAACTCTAAAGAATGG

ACATCCTTGTTTGACAATGACATTCCAGTTACCACTGACGATGTTTCATTGGCTGATAAG

GCAATTGAATCCACTGAAGAAGTTTCTCTGGTACCATCCAATCTGGAAGTCTCGACAACT

TCATTCTTACCCACTCCTGTTCTAGAAGATGCTAAACTGACTCAAACAAGAAAGGTTAAG

AAACCAAATTCAGTCGTTAAGAAGTCACATCATGTTGGAAAGGATGACGAATCGAGACTG

GATCATCTAGGTGTTGTTGCTTACAACCGCAAACAGCGTTCGATTCCACTTTCTCCAATT

GTGCCCGAATCCAGTGATCCTGCTGCTCTAAAACGTGCTAGAAACACTGAAGCCGCCAGG

CGTTCTCGTGCGAGAAAGTTGCAAAGAATGAAACAACTTGAAGACAAGGTTGAAGAATTG

CTTTCGAAAAATTATCACTTGGAAAATGAGGTTGCCAGATTAAAGAAATTAGTTGGCGAA

CGCTGA

## Primer3 Output

No mispriming library specified

Using 1-based sequence positions

WARNING: Left primer is unacceptable: Unacceptable GC content

OLIGO [start](http://frodo.wi.mit.edu/primer3/primer3_www_results_help.html#PRIMER_START)  [len](http://frodo.wi.mit.edu/primer3/primer3_www_results_help.html#PRIMER_LEN)  [tm](http://frodo.wi.mit.edu/primer3/primer3_www_results_help.html#PRIMER_TM)  [gc%](http://frodo.wi.mit.edu/primer3/primer3_www_results_help.html#PRIMER_GC)  [any](http://frodo.wi.mit.edu/primer3/primer3_www_results_help.html#PRIMER_ANY)  [3'](http://frodo.wi.mit.edu/primer3/primer3_www_results_help.html#PRIMER_REPEAT) [seq](http://frodo.wi.mit.edu/primer3/primer3_www_results_help.html#PRIMER_OLIGO_SEQ)

LEFT PRIMER 35 20 60.17 45.00 5.00 0.00 ATCCAATGGGTTTCTCACCA

RIGHT PRIMER 189 20 59.93 50.00 4.00 1.00 CGAAGGGGTATCCTGTTTGA

SEQUENCE SIZE: 846

INCLUDED REGION SIZE: 846

PRODUCT SIZE: 155, PAIR ANY COMPL: 3.00, PAIR 3' COMPL: 2.00

1 ATGTCCGAATATCAGCCAAGTTTATTTGCTTTAAATCCAATGGGTTTCTCACCATTGGAT

>>>>>>>>>>>>>>>>>>>>

61 GGTTCTAAATCAACCAACGAAAATGTATCTGCTTCCACTTCTACTGCCAAACCAATGGTT

121 GGCCAATTGATTTTTGATAAATTCATCAAGACTGAAGAGGATCCAATTATCAAACAGGAT

<<<<<<<<<<<

181 ACCCCTTCGAACCTTGATTTTGATTTTGCTCTTCCACAAACGGCAACTGCACCTGATGCC

<<<<<<<<<

241 AAGACCGTTTTGCCAATTCCGGAGCTAGATGACGCTGTAGTGGAATCTTTCTTTTCGTCA

301 AGCACTGATTCAACTCCAATGTTTGAGTATGAAAACCTAGAAGACAACTCTAAAGAATGG

361 ACATCCTTGTTTGACAATGACATTCCAGTTACCACTGACGATGTTTCATTGGCTGATAAG

421 GCAATTGAATCCACTGAAGAAGTTTCTCTGGTACCATCCAATCTGGAAGTCTCGACAACT

481 TCATTCTTACCCACTCCTGTTCTAGAAGATGCTAAACTGACTCAAACAAGAAAGGTTAAG

541 AAACCAAATTCAGTCGTTAAGAAGTCACATCATGTTGGAAAGGATGACGAATCGAGACTG

601 GATCATCTAGGTGTTGTTGCTTACAACCGCAAACAGCGTTCGATTCCACTTTCTCCAATT

661 GTGCCCGAATCCAGTGATCCTGCTGCTCTAAAACGTGCTAGAAACACTGAAGCCGCCAGG

721 CGTTCTCGTGCGAGAAAGTTGCAAAGAATGAAACAACTTGAAGACAAGGTTGAAGAATTG

781 CTTTCGAAAAATTATCACTTGGAAAATGAGGTTGCCAGATTAAAGAAATTAGTTGGCGAA

841 CGCTGA

UCSC In-Silico PCR

|  |  |
| --- | --- |
|  | >[chrV:139575-139729](http://genome.ucsc.edu/cgi-bin/hgTracks?hgsid=239079565&db=sacCer3&position=chrV:139575-139729&hgPcrResult=pack) 155bp ATCCAATGGGTTTCTCACCA CGAAGGGGTATCCTGTTTGA  ATCCAATGGGTTTCTCACCAttggatggttctaaatcaaccaacgaaaat  gtatctgcttccacttctactgccaaaccaatggttggccaattgatttt  tgataaattcatcaagactgaagaggatccaattaTCAAACAGGATACCC  CTTCG |

**4)YGL189C-RPS26A Chr 7**

**ACCESSION: NC_001139**

**GeneID:852686**

ATGCCAAAGAAGAGAGCTTCCAACGGTAGAAACAAGAAAGGTAGAGGTCACGTCAAACCA

GTCAGATGTGTCAACTGTTCCAAGTCTATTCCAAAGGATAAGGCTATCAAGAGAATGGCT

ATCAGAAACATTGTTGAAGCCGCTGCCGTCAGAGATTTGTCCGAAGCTTCTGTCTACCCT

GAATACGCTTTGCCAAAGACTTACAACAAGTTACACTACTGTGTTTCTTGTGCTATTCAC

GCCAGAATTGTCAGAGTCAGATCCAGAGAAGACAGAAAGAACAGAGCTCCACCTCAAAGA

CCAAGATTCAACAGAGAAAACAAGGTTTCCCCTGCTGATGCCGCCAAGAAGGCTTTATAA

## Primer3 Output

No mispriming library specified

Using 1-based sequence positions

OLIGO [start](http://frodo.wi.mit.edu/primer3/primer3_www_results_help.html#PRIMER_START)  [len](http://frodo.wi.mit.edu/primer3/primer3_www_results_help.html#PRIMER_LEN)  [tm](http://frodo.wi.mit.edu/primer3/primer3_www_results_help.html#PRIMER_TM)  [gc%](http://frodo.wi.mit.edu/primer3/primer3_www_results_help.html#PRIMER_GC)  [any](http://frodo.wi.mit.edu/primer3/primer3_www_results_help.html#PRIMER_ANY)  [3'](http://frodo.wi.mit.edu/primer3/primer3_www_results_help.html#PRIMER_REPEAT) [seq](http://frodo.wi.mit.edu/primer3/primer3_www_results_help.html#PRIMER_OLIGO_SEQ)

LEFT PRIMER 41 20 56.11 50.00 5.00 0.00 GTAGAGGTCACGTCAAACCA

RIGHT PRIMER 244 18 56.81 50.00 3.00 0.00 TGGCGTGAATAGCACAAG

SEQUENCE SIZE: 360

INCLUDED REGION SIZE: 360

PRODUCT SIZE: 204, PAIR ANY COMPL: 6.00, PAIR 3' COMPL: 0.00

1 ATGCCAAAGAAGAGAGCTTCCAACGGTAGAAACAAGAAAGGTAGAGGTCACGTCAAACCA

>>>>>>>>>>>>>>>>>>>>

61 GTCAGATGTGTCAACTGTTCCAAGTCTATTCCAAAGGATAAGGCTATCAAGAGAATGGCT

121 ATCAGAAACATTGTTGAAGCCGCTGCCGTCAGAGATTTGTCCGAAGCTTCTGTCTACCCT

181 GAATACGCTTTGCCAAAGACTTACAACAAGTTACACTACTGTGTTTCTTGTGCTATTCAC

<<<<<<<<<<<<<<

241 GCCAGAATTGTCAGAGTCAGATCCAGAGAAGACAGAAAGAACAGAGCTCCACCTCAAAGA

<<<<

301 CCAAGATTCAACAGAGAAAACAAGGTTTCCCCTGCTGATGCCGCCAAGAAGGCTTTATAA

UCSC In-Silico PCR

|  |  |
| --- | --- |
|  | >[chrVII:148345-148548](http://genome.ucsc.edu/cgi-bin/hgTracks?hgsid=219399449&db=sacCer3&position=chrVII:148345-148548&hgPcrResult=pack) 204bp GTAGAGGTCACGTCAAACCA TGGCGTGAATAGCACAAG  GTAGAGGTCACGTCAAACCAgtcagatgtgtcaactgttccaagtctatt  ccaaaggataaggctatcaagagaatggctatcagaaacattgttgaagc  cgctgccgtcagagatttgtccgaagcttctgtctaccctgaatacgctt  tgccaaagacttacaacaagttacactactgtgtttCTTGTGCTATTCAC  GCCA |

**5) YGR192C- TDH3 Chr 7**

**ACCESSION: NC_001139**

**GeneID:853106**

ATGGTTAGAGTTGCTATTAACGGTTTCGGTAGAATCGGTAGATTGGTCATGAGAATTGCT

TTGTCTAGACCAAACGTCGAAGTTGTTGCTTTGAACGACCCATTCATCACCAACGACTAC

GCTGCTTACATGTTCAAGTACGACTCCACTCACGGTAGATACGCTGGTGAAGTTTCCCAC

GATGACAAGCACATCATTGTCGATGGTAAGAAGATTGCTACTTACCAAGAAAGAGACCCA

GCTAACTTGCCATGGGGTTCTTCCAACGTTGACATCGCCATTGACTCCACTGGTGTTTTC

AAGGAATTAGACACTGCTCAAAAGCACATTGACGCTGGTGCCAAGAAGGTTGTTATCACT

GCTCCATCTTCCACCGCCCCAATGTTCGTCATGGGTGTTAACGAAGAAAAATACACTTCT

GACTTGAAGATTGTTTCCAACGCTTCTTGTACCACCAACTGTTTGGCTCCATTGGCCAAG

GTTATCAACGATGCTTTCGGTATTGAAGAAGGTTTGATGACCACTGTCCACTCTTTGACT

GCTACTCAAAAGACTGTTGACGGTCCATCCCACAAGGACTGGAGAGGTGGTAGAACCGCT

TCCGGTAACATCATCCCATCCTCCACCGGTGCTGCTAAGGCTGTCGGTAAGGTCTTGCCA

GAATTGCAAGGTAAGTTGACCGGTATGGCTTTCAGAGTCCCAACCGTCGATGTCTCCGTT

GTTGACTTGACTGTCAAGTTGAACAAGGAAACCACCTACGATGAAATCAAGAAGGTTGTT

AAGGCTGCCGCTGAAGGTAAGTTGAAGGGTGTTTTGGGTTACACCGAAGACGCTGTTGTC

TCCTCTGACTTCTTGGGTGACTCTCACTCTTCCATCTTCGATGCTTCCGCTGGTATCCAA

TTGTCTCCAAAGTTCGTCAAGTTGGTCTCCTGGTACGACAACGAATACGGTTACTCTACC

AGAGTTGTCGACTTGGTTGAACACGTTGCCAAGGCTTAA

## Primer3 Output

No mispriming library specified

Using 1-based sequence positions

OLIGO [start](http://frodo.wi.mit.edu/primer3/primer3_www_results_help.html#PRIMER_START)  [len](http://frodo.wi.mit.edu/primer3/primer3_www_results_help.html#PRIMER_LEN)  [tm](http://frodo.wi.mit.edu/primer3/primer3_www_results_help.html#PRIMER_TM)  [gc%](http://frodo.wi.mit.edu/primer3/primer3_www_results_help.html#PRIMER_GC)  [any](http://frodo.wi.mit.edu/primer3/primer3_www_results_help.html#PRIMER_ANY)  [3'](http://frodo.wi.mit.edu/primer3/primer3_www_results_help.html#PRIMER_REPEAT) [seq](http://frodo.wi.mit.edu/primer3/primer3_www_results_help.html#PRIMER_OLIGO_SEQ)

LEFT PRIMER 744 18 54.44 50.00 3.00 0.00 CAAGGAAACCACCTACGA

RIGHT PRIMER 880 20 55.06 50.00 2.00 0.00 CGAAGATGGAAGAGTGAGAG

SEQUENCE SIZE: 999

INCLUDED REGION SIZE: 999

PRODUCT SIZE: 137, PAIR ANY COMPL: 5.00, PAIR 3' COMPL: 0.00

1 ATGGTTAGAGTTGCTATTAACGGTTTCGGTAGAATCGGTAGATTGGTCATGAGAATTGCT

61 TTGTCTAGACCAAACGTCGAAGTTGTTGCTTTGAACGACCCATTCATCACCAACGACTAC

121 GCTGCTTACATGTTCAAGTACGACTCCACTCACGGTAGATACGCTGGTGAAGTTTCCCAC

181 GATGACAAGCACATCATTGTCGATGGTAAGAAGATTGCTACTTACCAAGAAAGAGACCCA

241 GCTAACTTGCCATGGGGTTCTTCCAACGTTGACATCGCCATTGACTCCACTGGTGTTTTC

301 AAGGAATTAGACACTGCTCAAAAGCACATTGACGCTGGTGCCAAGAAGGTTGTTATCACT

361 GCTCCATCTTCCACCGCCCCAATGTTCGTCATGGGTGTTAACGAAGAAAAATACACTTCT

421 GACTTGAAGATTGTTTCCAACGCTTCTTGTACCACCAACTGTTTGGCTCCATTGGCCAAG

481 GTTATCAACGATGCTTTCGGTATTGAAGAAGGTTTGATGACCACTGTCCACTCTTTGACT

541 GCTACTCAAAAGACTGTTGACGGTCCATCCCACAAGGACTGGAGAGGTGGTAGAACCGCT

601 TCCGGTAACATCATCCCATCCTCCACCGGTGCTGCTAAGGCTGTCGGTAAGGTCTTGCCA

661 GAATTGCAAGGTAAGTTGACCGGTATGGCTTTCAGAGTCCCAACCGTCGATGTCTCCGTT

721 GTTGACTTGACTGTCAAGTTGAACAAGGAAACCACCTACGATGAAATCAAGAAGGTTGTT

>>>>>>>>>>>>>>>>>>

781 AAGGCTGCCGCTGAAGGTAAGTTGAAGGGTGTTTTGGGTTACACCGAAGACGCTGTTGTC

841 TCCTCTGACTTCTTGGGTGACTCTCACTCTTCCATCTTCGATGCTTCCGCTGGTATCCAA

<<<<<<<<<<<<<<<<<<<<

901 TTGTCTCCAAAGTTCGTCAAGTTGGTCTCCTGGTACGACAACGAATACGGTTACTCTACC

961 AGAGTTGTCGACTTGGTTGAACACGTTGCCAAGGCTTAA

UCSC In-Silico PCR

|  |  |
| --- | --- |
|  | >[chrVII:882931-883067](http://genome.ucsc.edu/cgi-bin/hgTracks?hgsid=219399449&db=sacCer3&position=chrVII:882931-883067&hgPcrResult=pack) 137bp CAAGGAAACCACCTACGA CGAAGATGGAAGAGTGAGAG  CAAGGAAACCACCTACGAtgaaatcaagaaggttgttaaggctgccgctg  aaggtaagttgaagggtgttttgggttacaccgaagacgctgttgtctcc  tctgacttcttgggtgaCTCTCACTCTTCCATCTTCG |

**6) YDL192W- ARF1 Chr 4**

**ACCESSION: NC_001136**

**GeneID:851335**

ATGGGTTTGTTTGCCTCTAAGTTGTTCAGTAACCTTTTTGGTAACAAAGAAATGCGTATT

CTTATGGTTGGTCTTGATGGTGCTGGTAAGACCACCGTTTTGTACAAGTTGAAATTGGGT

GAAGTTATCACTACCATTCCAACAATTGGTTTCAACGTTGAAACTGTCCAATATAAGAAC

ATTTCATTCACTGTCTGGGATGTCGGTGGACAAGACAGAATTAGATCTCTATGGAGACAC

TACTACAGAAACACTGAAGGTGTTATCTTTGTTGTCGATTCTAACGATAGATCGCGTATT

GGTGAAGCTAGAGAAGTTATGCAAAGAATGTTGAACGAAGATGAATTGAGAAACGCCGCT

TGGTTGGTGTTCGCTAACAAGCAAGATTTGCCAGAAGCCATGTCTGCTGCTGAAATCACT

GAAAAACTAGGTTTACATTCTATTAGAAACCGTCCATGGTTTATCCAAGCCACGTGTGCT

ACCTCCGGTGAAGGTTTGTATGAAGGTTTGGAATGGTTAAGTAACAGTTTGAAAAACTCA

ACTTAA

## Primer3 Output

No mispriming library specified

Using 1-based sequence positions

WARNING: Left primer is unacceptable: Unacceptable GC content; Right primer is unacceptable: Unacceptable GC content

OLIGO [start](http://frodo.wi.mit.edu/primer3/primer3_www_results_help.html#PRIMER_START)  [len](http://frodo.wi.mit.edu/primer3/primer3_www_results_help.html#PRIMER_LEN)  [tm](http://frodo.wi.mit.edu/primer3/primer3_www_results_help.html#PRIMER_TM)  [gc%](http://frodo.wi.mit.edu/primer3/primer3_www_results_help.html#PRIMER_GC)  [any](http://frodo.wi.mit.edu/primer3/primer3_www_results_help.html#PRIMER_ANY)  [3'](http://frodo.wi.mit.edu/primer3/primer3_www_results_help.html#PRIMER_REPEAT) [seq](http://frodo.wi.mit.edu/primer3/primer3_www_results_help.html#PRIMER_OLIGO_SEQ)

LEFT PRIMER 281 22 58.81 45.45 6.00 0.00 CTAACGATAGATCGCGTATTGG

RIGHT PRIMER 395 22 59.54 40.91 6.00 0.00 TCTGGCAAATCTTGCTTGTTAG

SEQUENCE SIZE: 546

INCLUDED REGION SIZE: 546

PRODUCT SIZE: 115, PAIR ANY COMPL: 5.00, PAIR 3' COMPL: 0.00

1 ATGGGTTTGTTTGCCTCTAAGTTGTTCAGTAACCTTTTTGGTAACAAAGAAATGCGTATT

61 CTTATGGTTGGTCTTGATGGTGCTGGTAAGACCACCGTTTTGTACAAGTTGAAATTGGGT

121 GAAGTTATCACTACCATTCCAACAATTGGTTTCAACGTTGAAACTGTCCAATATAAGAAC

181 ATTTCATTCACTGTCTGGGATGTCGGTGGACAAGACAGAATTAGATCTCTATGGAGACAC

241 TACTACAGAAACACTGAAGGTGTTATCTTTGTTGTCGATTCTAACGATAGATCGCGTATT

>>>>>>>>>>>>>>>>>>>>

301 GGTGAAGCTAGAGAAGTTATGCAAAGAATGTTGAACGAAGATGAATTGAGAAACGCCGCT

>>

361 TGGTTGGTGTTCGCTAACAAGCAAGATTTGCCAGAAGCCATGTCTGCTGCTGAAATCACT

<<<<<<<<<<<<<<<<<<<<<<

421 GAAAAACTAGGTTTACATTCTATTAGAAACCGTCCATGGTTTATCCAAGCCACGTGTGCT

481 ACCTCCGGTGAAGGTTTGTATGAAGGTTTGGAATGGTTAAGTAACAGTTTGAAAAACTCA

541 ACTTAA

UCSC In-Silico PCR

|  |  |
| --- | --- |
|  | >[chrIV:116601+116715](http://genome.ucsc.edu/cgi-bin/hgTracks?hgsid=239079565&db=sacCer3&position=chrIV:116601-116715&hgPcrResult=pack) 115bp CTAACGATAGATCGCGTATTGG TCTGGCAAATCTTGCTTGTTAG  CTAACGATAGATCGCGTATTGGtgaagctagagaagttatgcaaagaatg  ttgaacgaagatgaattgagaaacgccgcttggttggtgttcgCTAACAA  GCAAGATTTGCCAGA |

**7) YDR050C- TPI1 Chr 4**

**ACCESSION: NC_001136**

**GeneID:851620**

ATGGCTAGAACTTTCTTTGTCGGTGGTAACTTTAAATTAAACGGTTCCAAACAATCCATT

AAGGAAATTGTTGAAAGATTGAACACTGCTTCTATCCCAGAAAATGTCGAAGTTGTTATC

TGTCCTCCAGCTACCTACTTAGACTACTCTGTCTCTTTGGTTAAGAAGCCACAAGTCACT

GTCGGTGCTCAAAACGCCTACTTGAAGGCTTCTGGTGCTTTCACCGGTGAAAACTCCGTT

GACCAAATCAAGGATGTTGGTGCTAAGTGGGTTATTTTGGGTCACTCCGAAAGAAGATCT

TACTTCCACGAAGATGACAAGTTCATTGCTGACAAGACCAAGTTCGCTTTAGGTCAAGGT

GTCGGTGTCATCTTGTGTATCGGTGAAACTTTGGAAGAAAAGAAGGCCGGTAAGACTTTG

GATGTTGTTGAAAGACAATTGAACGCTGTCTTGGAAGAAGTTAAGGACTGGACTAACGTC

GTTGTCGCTTACGAACCAGTCTGGGCCATTGGTACCGGTTTGGCTGCTACTCCAGAAGAT

GCTCAAGATATTCACGCTTCCATCAGAAAGTTCTTGGCTTCCAAGTTGGGTGACAAGGCT

GCCAGCGAATTGAGAATCTTATACGGTGGTTCCGCTAACGGTAGCAACGCCGTTACCTTC

AAGGACAAGGCTGATGTCGATGGTTTCTTGGTCGGTGGTGCTTCTTTGAAGCCAGAATTT

GTTGATATCATCAACTCTAGAAACTAA

## Primer3 Output

No mispriming library specified

Using 1-based sequence positions

OLIGO [start](http://frodo.wi.mit.edu/primer3/primer3_www_results_help.html#PRIMER_START)  [len](http://frodo.wi.mit.edu/primer3/primer3_www_results_help.html#PRIMER_LEN)  [tm](http://frodo.wi.mit.edu/primer3/primer3_www_results_help.html#PRIMER_TM)  [gc%](http://frodo.wi.mit.edu/primer3/primer3_www_results_help.html#PRIMER_GC)  [any](http://frodo.wi.mit.edu/primer3/primer3_www_results_help.html#PRIMER_ANY)  [3'](http://frodo.wi.mit.edu/primer3/primer3_www_results_help.html#PRIMER_REPEAT) [seq](http://frodo.wi.mit.edu/primer3/primer3_www_results_help.html#PRIMER_OLIGO_SEQ)

LEFT PRIMER 359 20 56.84 50.00 3.00 0.00 GTGTCGGTGTCATCTTGTGT

RIGHT PRIMER 482 20 56.35 50.00 5.00 0.00 ACGACGTTAGTCCAGTCCTT

SEQUENCE SIZE: 747

INCLUDED REGION SIZE: 747

PRODUCT SIZE: 124, PAIR ANY COMPL: 4.00, PAIR 3' COMPL: 0.00

1 ATGGCTAGAACTTTCTTTGTCGGTGGTAACTTTAAATTAAACGGTTCCAAACAATCCATT

61 AAGGAAATTGTTGAAAGATTGAACACTGCTTCTATCCCAGAAAATGTCGAAGTTGTTATC

121 TGTCCTCCAGCTACCTACTTAGACTACTCTGTCTCTTTGGTTAAGAAGCCACAAGTCACT

181 GTCGGTGCTCAAAACGCCTACTTGAAGGCTTCTGGTGCTTTCACCGGTGAAAACTCCGTT

241 GACCAAATCAAGGATGTTGGTGCTAAGTGGGTTATTTTGGGTCACTCCGAAAGAAGATCT

301 TACTTCCACGAAGATGACAAGTTCATTGCTGACAAGACCAAGTTCGCTTTAGGTCAAGGT

>>

361 GTCGGTGTCATCTTGTGTATCGGTGAAACTTTGGAAGAAAAGAAGGCCGGTAAGACTTTG

>>>>>>>>>>>>>>>>>>

421 GATGTTGTTGAAAGACAATTGAACGCTGTCTTGGAAGAAGTTAAGGACTGGACTAACGTC

<<<<<<<<<<<<<<<<<<

481 GTTGTCGCTTACGAACCAGTCTGGGCCATTGGTACCGGTTTGGCTGCTACTCCAGAAGAT

<<

541 GCTCAAGATATTCACGCTTCCATCAGAAAGTTCTTGGCTTCCAAGTTGGGTGACAAGGCT

601 GCCAGCGAATTGAGAATCTTATACGGTGGTTCCGCTAACGGTAGCAACGCCGTTACCTTC

661 AAGGACAAGGCTGATGTCGATGGTTTCTTGGTCGGTGGTGCTTCTTTGAAGCCAGAATTT

721 GTTGATATCATCAACTCTAGAAACTAA

UCSC In-Silico PCR

|  |  |
| --- | --- |
|  | >[chrIV:555991-556114](http://genome.ucsc.edu/cgi-bin/hgTracks?hgsid=239079565&db=sacCer3&position=chrIV:555991-556114&hgPcrResult=pack) 124bp GTGTCGGTGTCATCTTGTGT ACGACGTTAGTCCAGTCCTT  GTGTCGGTGTCATCTTGTGTatcggtgaaactttggaagaaaagaaggcc  ggtaagactttggatgttgttgaaagacaattgaacgctgtcttggaaga  agttAAGGACTGGACTAACGTCGT |

**8) YAL038W- CDC19 Chr 1**

**ACCESSION: NC_001133**

**GeneID:851193**

ATGTCTAGATTAGAAAGATTGACCTCATTAAACGTTGTTGCTGGTTCTGACTTGAGAAGA

ACCTCCATCATTGGTACCATCGGTCCAAAGACCAACAACCCAGAAACCTTGGTTGCTTTG

AGAAAGGCTGGTTTGAACATTGTCCGTATGAACTTCTCTCACGGTTCTTACGAATACCAC

AAGTCTGTCATTGACAACGCCAGAAAGTCCGAAGAATTGTACCCAGGTAGACCATTGGCC

ATTGCTTTGGACACCAAGGGTCCAGAAATCAGAACTGGTACCACCACCAACGATGTTGAC

TACCCAATCCCACCAAACCACGAAATGATCTTCACCACCGATGACAAGTACGCTAAGGCT

TGTGACGACAAGATCATGTACGTTGACTACAAGAACATCACCAAGGTCATCTCCGCTGGT

AGAATCATCTACGTTGATGATGGTGTTTTGTCTTTCCAAGTTTTGGAAGTCGTTGACGAC

AAGACTTTGAAGGTCAAGGCTTTGAACGCCGGTAAGATCTGTTCCCACAAGGGTGTCAAC

TTACCAGGTACCGATGTCGATTTGCCAGCTTTGTCTGAAAAGGACAAGGAAGATTTGAGA

TTCGGTGTCAAGAACGGTGTCCACATGGTCTTCGCTTCTTTCATCAGAACCGCCAACGAT

GTTTTGACCATCAGAGAAGTCTTGGGTGAACAAGGTAAGGACGTCAAGATCATTGTCAAG

ATTGAAAACCAACAAGGTGTTAACAACTTCGACGAAATCTTGAAGGTCACTGACGGTGTT

ATGGTTGCCAGAGGTGACTTGGGTATTGAAATCCCAGCCCCAGAAGTCTTGGCTGTCCAA

AAGAAATTGATTGCTAAGTCTAACTTGGCTGGTAAGCCAGTTATCTGTGCTACCCAAATG

TTGGAATCCATGACTTACAACCCAAGACCAACCAGAGCTGAAGTTTCCGATGTCGGTAAC

GCTATCTTGGATGGTGCTGACTGTGTTATGTTGTCTGGTGAAACCGCCAAGGGTAACTAC

CCAATCAACGCCGTTACCACTATGGCTGAAACCGCTGTCATTGCTGAACAAGCTATCGCT

TACTTGCCAAACTACGATGACATGAGAAACTGTACTCCAAAGCCAACCTCCACCACCGAA

ACCGTCGCTGCCTCCGCTGTCGCTGCTGTTTTCGAACAAAAGGCCAAGGCTATCATTGTC

TTGTCCACTTCCGGTACCACCCCAAGATTGGTTTCCAAGTACAGACCAAACTGTCCAATC

ATCTTGGTTACCAGATGCCCAAGAGCTGCTAGATTCTCTCACTTGTACAGAGGTGTCTTC

CCATTCGTTTTCGAAAAGGAACCTGTCTCTGACTGGACTGATGATGTTGAAGCCCGTATC

AACTTCGGTATTGAAAAGGCTAAGGAATTCGGTATCTTGAAGAAGGGTGACACTTACGTT

TCCATCCAAGGTTTCAAGGCCGGTGCTGGTCACTCCAACACTTTGCAAGTCTCTACCGTT

TAA

## Primer3 Output

No mispriming library specified

Using 1-based sequence positions

OLIGO [start](http://frodo.wi.mit.edu/primer3/primer3_www_results_help.html#PRIMER_START)  [len](http://frodo.wi.mit.edu/primer3/primer3_www_results_help.html#PRIMER_LEN)  [tm](http://frodo.wi.mit.edu/primer3/primer3_www_results_help.html#PRIMER_TM)  [gc%](http://frodo.wi.mit.edu/primer3/primer3_www_results_help.html#PRIMER_GC)  [any](http://frodo.wi.mit.edu/primer3/primer3_www_results_help.html#PRIMER_ANY)  [3'](http://frodo.wi.mit.edu/primer3/primer3_www_results_help.html#PRIMER_REPEAT) [seq](http://frodo.wi.mit.edu/primer3/primer3_www_results_help.html#PRIMER_OLIGO_SEQ)

LEFT PRIMER 789 20 55.59 50.00 3.00 0.00 CAGAGGTGACTTGGGTATTG

RIGHT PRIMER 933 20 57.02 50.00 3.00 0.00 GGTTGGTCTTGGGTTGTAAG

SEQUENCE SIZE: 1503

INCLUDED REGION SIZE: 1503

PRODUCT SIZE: 145, PAIR ANY COMPL: 3.00, PAIR 3' COMPL: 0.00

1 ATGTCTAGATTAGAAAGATTGACCTCATTAAACGTTGTTGCTGGTTCTGACTTGAGAAGA

61 ACCTCCATCATTGGTACCATCGGTCCAAAGACCAACAACCCAGAAACCTTGGTTGCTTTG

121 AGAAAGGCTGGTTTGAACATTGTCCGTATGAACTTCTCTCACGGTTCTTACGAATACCAC

181 AAGTCTGTCATTGACAACGCCAGAAAGTCCGAAGAATTGTACCCAGGTAGACCATTGGCC

241 ATTGCTTTGGACACCAAGGGTCCAGAAATCAGAACTGGTACCACCACCAACGATGTTGAC

301 TACCCAATCCCACCAAACCACGAAATGATCTTCACCACCGATGACAAGTACGCTAAGGCT

361 TGTGACGACAAGATCATGTACGTTGACTACAAGAACATCACCAAGGTCATCTCCGCTGGT

421 AGAATCATCTACGTTGATGATGGTGTTTTGTCTTTCCAAGTTTTGGAAGTCGTTGACGAC

481 AAGACTTTGAAGGTCAAGGCTTTGAACGCCGGTAAGATCTGTTCCCACAAGGGTGTCAAC

541 TTACCAGGTACCGATGTCGATTTGCCAGCTTTGTCTGAAAAGGACAAGGAAGATTTGAGA

601 TTCGGTGTCAAGAACGGTGTCCACATGGTCTTCGCTTCTTTCATCAGAACCGCCAACGAT

661 GTTTTGACCATCAGAGAAGTCTTGGGTGAACAAGGTAAGGACGTCAAGATCATTGTCAAG

721 ATTGAAAACCAACAAGGTGTTAACAACTTCGACGAAATCTTGAAGGTCACTGACGGTGTT

781 ATGGTTGCCAGAGGTGACTTGGGTATTGAAATCCCAGCCCCAGAAGTCTTGGCTGTCCAA

>>>>>>>>>>>>>>>>>>>>

841 AAGAAATTGATTGCTAAGTCTAACTTGGCTGGTAAGCCAGTTATCTGTGCTACCCAAATG

901 TTGGAATCCATGACTTACAACCCAAGACCAACCAGAGCTGAAGTTTCCGATGTCGGTAAC

<<<<<<<<<<<<<<<<<<<<

961 GCTATCTTGGATGGTGCTGACTGTGTTATGTTGTCTGGTGAAACCGCCAAGGGTAACTAC

1021 CCAATCAACGCCGTTACCACTATGGCTGAAACCGCTGTCATTGCTGAACAAGCTATCGCT

1081 TACTTGCCAAACTACGATGACATGAGAAACTGTACTCCAAAGCCAACCTCCACCACCGAA

1141 ACCGTCGCTGCCTCCGCTGTCGCTGCTGTTTTCGAACAAAAGGCCAAGGCTATCATTGTC

1201 TTGTCCACTTCCGGTACCACCCCAAGATTGGTTTCCAAGTACAGACCAAACTGTCCAATC

1261 ATCTTGGTTACCAGATGCCCAAGAGCTGCTAGATTCTCTCACTTGTACAGAGGTGTCTTC

1321 CCATTCGTTTTCGAAAAGGAACCTGTCTCTGACTGGACTGATGATGTTGAAGCCCGTATC

1381 AACTTCGGTATTGAAAAGGCTAAGGAATTCGGTATCTTGAAGAAGGGTGACACTTACGTT

1441 TCCATCCAAGGTTTCAAGGCCGGTGCTGGTCACTCCAACACTTTGCAAGTCTCTACCGTT

1501 TAA

UCSC In-Silico PCR

|  |  |
| --- | --- |
|  | >[chrI:72574+72718](http://genome.ucsc.edu/cgi-bin/hgTracks?hgsid=219399449&db=sacCer3&position=chrI:72574-72718&hgPcrResult=pack) 145bp CAGAGGTGACTTGGGTATTG GGTTGGTCTTGGGTTGTAAG  CAGAGGTGACTTGGGTATTGaaatcccagccccagaagtcttggctgtcc  aaaagaaattgattgctaagtctaacttggctggtaagccagttatctgt  gctacccaaatgttggaatccatgaCTTACAACCCAAGACCAACC |

**9) YCR012W- PGK1 Chr 3**

**ACCESSION: NC_001135**

**GeneID:850370**

ATGTCTTTATCTTCAAAGTTGTCTGTCCAAGATTTGGACTTGAAGGACAAGCGTGTCTTC

ATCAGAGTTGACTTCAACGTCCCATTGGACGGTAAGAAGATCACTTCTAACCAAAGAATT

GTTGCTGCTTTGCCAACCATCAAGTACGTTTTGGAACACCACCCAAGATACGTTGTCTTG

GCTTCTCACTTGGGTAGACCAAACGGTGAAAGAAACGAAAAATACTCTTTGGCTCCAGTT

GCTAAGGAATTGCAATCATTGTTGGGTAAGGATGTCACCTTCTTGAACGACTGTGTCGGT

CCAGAAGTTGAAGCCGCTGTCAAGGCTTCTGCCCCAGGTTCCGTTATTTTGTTGGAAAAC

TTGCGTTACCACATCGAAGAAGAAGGTTCCAGAAAGGTCGATGGTCAAAAGGTCAAGGCT

TCCAAGGAAGATGTTCAAAAGTTCAGACACGAATTGAGCTCTTTGGCTGATGTTTACATC

AACGATGCCTTCGGTACCGCTCACAGAGCTCACTCTTCTATGGTCGGTTTCGACTTGCCA

CAACGTGCTGCCGGTTTCTTGTTGGAAAAGGAATTGAAGTACTTCGGTAAGGCTTTGGAG

AACCCAACCAGACCATTCTTGGCCATCTTAGGTGGTGCCAAGGTTGCTGACAAGATTCAA

TTGATTGACAACTTGTTGGACAAGGTCGACTCTATCATCATTGGTGGTGGTATGGCTTTC

ACCTTCAAGAAGGTTTTGGAAAACACTGAAATCGGTGACTCCATCTTCGACAAGGCTGGT

GCTGAAATCGTTCCAAAGTTGATGGAAAAGGCCAAGGCCAAGGGTGTCGAAGTCGTCTTG

CCAGTCGACTTCATCATTGCTGATGCTTTCTCTGCTGATGCCAACACCAAGACTGTCACT

GACAAGGAAGGTATTCCAGCTGGCTGGCAAGGGTTGGACAATGGTCCAGAATCTAGAAAG

TTGTTTGCTGCTACTGTTGCAAAGGCTAAGACCATTGTCTGGAACGGTCCACCAGGTGTT

TTCGAATTCGAAAAGTTCGCTGCTGGTACTAAGGCTTTGTTAGACGAAGTTGTCAAGAGC

TCTGCTGCTGGTAACACCGTCATCATTGGTGGTGGTGACACTGCCACTGTCGCTAAGAAG

TACGGTGTCACTGACAAGATCTCCCATGTCTCTACTGGTGGTGGTGCTTCTTTGGAATTA

TTGGAAGGTAAGGAATTGCCAGGTGTTGCTTTCTTATCCGAAAAGAAATAA

## Primer3 Output

No mispriming library specified

Using 1-based sequence positions

OLIGO [start](http://frodo.wi.mit.edu/primer3/primer3_www_results_help.html#PRIMER_START)  [len](http://frodo.wi.mit.edu/primer3/primer3_www_results_help.html#PRIMER_LEN)  [tm](http://frodo.wi.mit.edu/primer3/primer3_www_results_help.html#PRIMER_TM)  [gc%](http://frodo.wi.mit.edu/primer3/primer3_www_results_help.html#PRIMER_GC)  [any](http://frodo.wi.mit.edu/primer3/primer3_www_results_help.html#PRIMER_ANY)  [3'](http://frodo.wi.mit.edu/primer3/primer3_www_results_help.html#PRIMER_REPEAT) [seq](http://frodo.wi.mit.edu/primer3/primer3_www_results_help.html#PRIMER_OLIGO_SEQ)

LEFT PRIMER 153 19 55.13 52.63 2.00 0.00 GGAACACCACCCAAGATAC

RIGHT PRIMER 279 19 55.13 52.63 3.00 0.00 GGTGACATCCTTACCCAAC

SEQUENCE SIZE: 1251

INCLUDED REGION SIZE: 1251

PRODUCT SIZE: 127, PAIR ANY COMPL: 4.00, PAIR 3' COMPL: 0.00

1 ATGTCTTTATCTTCAAAGTTGTCTGTCCAAGATTTGGACTTGAAGGACAAGCGTGTCTTC

61 ATCAGAGTTGACTTCAACGTCCCATTGGACGGTAAGAAGATCACTTCTAACCAAAGAATT

121 GTTGCTGCTTTGCCAACCATCAAGTACGTTTTGGAACACCACCCAAGATACGTTGTCTTG

>>>>>>>>>>>>>>>>>>>

181 GCTTCTCACTTGGGTAGACCAAACGGTGAAAGAAACGAAAAATACTCTTTGGCTCCAGTT

241 GCTAAGGAATTGCAATCATTGTTGGGTAAGGATGTCACCTTCTTGAACGACTGTGTCGGT

<<<<<<<<<<<<<<<<<<<

301 CCAGAAGTTGAAGCCGCTGTCAAGGCTTCTGCCCCAGGTTCCGTTATTTTGTTGGAAAAC

361 TTGCGTTACCACATCGAAGAAGAAGGTTCCAGAAAGGTCGATGGTCAAAAGGTCAAGGCT

421 TCCAAGGAAGATGTTCAAAAGTTCAGACACGAATTGAGCTCTTTGGCTGATGTTTACATC

481 AACGATGCCTTCGGTACCGCTCACAGAGCTCACTCTTCTATGGTCGGTTTCGACTTGCCA

541 CAACGTGCTGCCGGTTTCTTGTTGGAAAAGGAATTGAAGTACTTCGGTAAGGCTTTGGAG

601 AACCCAACCAGACCATTCTTGGCCATCTTAGGTGGTGCCAAGGTTGCTGACAAGATTCAA

661 TTGATTGACAACTTGTTGGACAAGGTCGACTCTATCATCATTGGTGGTGGTATGGCTTTC

721 ACCTTCAAGAAGGTTTTGGAAAACACTGAAATCGGTGACTCCATCTTCGACAAGGCTGGT

781 GCTGAAATCGTTCCAAAGTTGATGGAAAAGGCCAAGGCCAAGGGTGTCGAAGTCGTCTTG

841 CCAGTCGACTTCATCATTGCTGATGCTTTCTCTGCTGATGCCAACACCAAGACTGTCACT

901 GACAAGGAAGGTATTCCAGCTGGCTGGCAAGGGTTGGACAATGGTCCAGAATCTAGAAAG

961 TTGTTTGCTGCTACTGTTGCAAAGGCTAAGACCATTGTCTGGAACGGTCCACCAGGTGTT

1021 TTCGAATTCGAAAAGTTCGCTGCTGGTACTAAGGCTTTGTTAGACGAAGTTGTCAAGAGC

1081 TCTGCTGCTGGTAACACCGTCATCATTGGTGGTGGTGACACTGCCACTGTCGCTAAGAAG

1141 TACGGTGTCACTGACAAGATCTCCCATGTCTCTACTGGTGGTGGTGCTTCTTTGGAATTA

1201 TTGGAAGGTAAGGAATTGCCAGGTGTTGCTTTCTTATCCGAAAAGAAATAA

UCSC In-Silico PCR

|  |  |
| --- | --- |
|  | >[chrIII:137898+138024](http://genome.ucsc.edu/cgi-bin/hgTracks?hgsid=219399449&db=sacCer3&position=chrIII:137898-138024&hgPcrResult=pack) 127bp GGAACACCACCCAAGATAC GGTGACATCCTTACCCAAC  GGAACACCACCCAAGATACgttgtcttggcttctcacttgggtagaccaa  acggtgaaagaaacgaaaaatactctttggctccagttgctaaggaattg  caatcattGTTGGGTAAGGATGTCACC |

**10) YOL086C- ADH1 Chr 15**

**ACCESSION NC_001147**

**GeneID:854068**

ATGTCTATCCCAGAAACTCAAAAAGGTGTTATCTTCTACGAATCCCACGGTAAGTTGGAA

TACAAAGATATTCCAGTTCCAAAGCCAAAGGCCAACGAATTGTTGATCAACGTTAAATAC

TCTGGTGTCTGTCACACTGACTTGCACGCTTGGCACGGTGACTGGCCATTGCCAGTTAAG

CTACCATTAGTCGGTGGTCACGAAGGTGCCGGTGTCGTTGTCGGCATGGGTGAAAACGTT

AAGGGCTGGAAGATCGGTGACTACGCCGGTATCAAATGGTTGAACGGTTCTTGTATGGCC

TGTGAATACTGTGAATTGGGTAACGAATCCAACTGTCCTCACGCTGACTTGTCTGGTTAC

ACCCACGACGGTTCTTTCCAACAATACGCTACCGCTGACGCTGTTCAAGCCGCTCACATT

CCTCAAGGTACCGACTTGGCCCAAGTCGCCCCCATCTTGTGTGCTGGTATCACCGTCTAC

AAGGCTTTGAAGTCTGCTAACTTGATGGCCGGTCACTGGGTTGCTATCTCCGGTGCTGCT

GGTGGTCTAGGTTCTTTGGCTGTTCAATACGCCAAGGCTATGGGTTACAGAGTCTTGGGT

ATTGACGGTGGTGAAGGTAAGGAAGAATTATTCAGATCCATCGGTGGTGAAGTCTTCATT

GACTTCACTAAGGAAAAGGACATTGTCGGTGCTGTTCTAAAGGCCACTGACGGTGGTGCT

CACGGTGTCATCAACGTTTCCGTTTCCGAAGCCGCTATTGAAGCTTCTACCAGATACGTT

AGAGCTAACGGTACCACCGTTTTGGTCGGTATGCCAGCTGGTGCCAAGTGTTGTTCTGAT

GTCTTCAACCAAGTCGTCAAGTCCATCTCTATTGTTGGTTCTTACGTCGGTAACAGAGCT

GACACCAGAGAAGCTTTGGACTTCTTCGCCAGAGGTTTGGTCAAGTCTCCAATCAAGGTT

GTCGGCTTGTCTACCTTGCCAGAAATTTACGAAAAGATGGAAAAGGGTCAAATCGTTGGT

AGATACGTTGTTGACACTTCTAAATAA

## Primer3 Output

No mispriming library specified

Using 1-based sequence positions

OLIGO [start](http://frodo.wi.mit.edu/primer3/primer3_www_results_help.html#PRIMER_START)  [len](http://frodo.wi.mit.edu/primer3/primer3_www_results_help.html#PRIMER_LEN)  [tm](http://frodo.wi.mit.edu/primer3/primer3_www_results_help.html#PRIMER_TM)  [gc%](http://frodo.wi.mit.edu/primer3/primer3_www_results_help.html#PRIMER_GC)  [any](http://frodo.wi.mit.edu/primer3/primer3_www_results_help.html#PRIMER_ANY)  [3'](http://frodo.wi.mit.edu/primer3/primer3_www_results_help.html#PRIMER_REPEAT) [seq](http://frodo.wi.mit.edu/primer3/primer3_www_results_help.html#PRIMER_OLIGO_SEQ)

LEFT PRIMER 850 20 55.72 50.00 2.00 0.00 CAAGTCGTCAAGTCCATCTC

RIGHT PRIMER 974 19 55.29 52.63 2.00 0.00 GTAGACAAGCCGACAACCT

SEQUENCE SIZE: 1047

INCLUDED REGION SIZE: 1047

PRODUCT SIZE: 125, PAIR ANY COMPL: 5.00, PAIR 3' COMPL: 0.00

1 ATGTCTATCCCAGAAACTCAAAAAGGTGTTATCTTCTACGAATCCCACGGTAAGTTGGAA

61 TACAAAGATATTCCAGTTCCAAAGCCAAAGGCCAACGAATTGTTGATCAACGTTAAATAC

121 TCTGGTGTCTGTCACACTGACTTGCACGCTTGGCACGGTGACTGGCCATTGCCAGTTAAG

181 CTACCATTAGTCGGTGGTCACGAAGGTGCCGGTGTCGTTGTCGGCATGGGTGAAAACGTT

241 AAGGGCTGGAAGATCGGTGACTACGCCGGTATCAAATGGTTGAACGGTTCTTGTATGGCC

301 TGTGAATACTGTGAATTGGGTAACGAATCCAACTGTCCTCACGCTGACTTGTCTGGTTAC

361 ACCCACGACGGTTCTTTCCAACAATACGCTACCGCTGACGCTGTTCAAGCCGCTCACATT

421 CCTCAAGGTACCGACTTGGCCCAAGTCGCCCCCATCTTGTGTGCTGGTATCACCGTCTAC

481 AAGGCTTTGAAGTCTGCTAACTTGATGGCCGGTCACTGGGTTGCTATCTCCGGTGCTGCT

541 GGTGGTCTAGGTTCTTTGGCTGTTCAATACGCCAAGGCTATGGGTTACAGAGTCTTGGGT

601 ATTGACGGTGGTGAAGGTAAGGAAGAATTATTCAGATCCATCGGTGGTGAAGTCTTCATT

661 GACTTCACTAAGGAAAAGGACATTGTCGGTGCTGTTCTAAAGGCCACTGACGGTGGTGCT

721 CACGGTGTCATCAACGTTTCCGTTTCCGAAGCCGCTATTGAAGCTTCTACCAGATACGTT

781 AGAGCTAACGGTACCACCGTTTTGGTCGGTATGCCAGCTGGTGCCAAGTGTTGTTCTGAT

841 GTCTTCAACCAAGTCGTCAAGTCCATCTCTATTGTTGGTTCTTACGTCGGTAACAGAGCT

>>>>>>>>>>>>>>>>>>>>

901 GACACCAGAGAAGCTTTGGACTTCTTCGCCAGAGGTTTGGTCAAGTCTCCAATCAAGGTT

<<<<<

961 GTCGGCTTGTCTACCTTGCCAGAAATTTACGAAAAGATGGAAAAGGGTCAAATCGTTGGT

<<<<<<<<<<<<<<

1021 AGATACGTTGTTGACACTTCTAAATAA

UCSC In-Silico PCR

|  |  |
| --- | --- |
|  | >[chrXV:159621-159745](http://genome.ucsc.edu/cgi-bin/hgTracks?hgsid=219399449&db=sacCer3&position=chrXV:159621-159745&hgPcrResult=pack) 125bp CAAGTCGTCAAGTCCATCTC GTAGACAAGCCGACAACCT  CAAGTCGTCAAGTCCATCTCtattgttggttcttacgtcggtaacagagc  tgacaccagagaagctttggacttcttcgccagaggtttggtcaagtctc  caatcaAGGTTGTCGGCTTGTCTAC |

**11-) YFL039C- ACT1 Chr 6**

**ACCESSION: NC_001138**

**GeneID:850504**

ATGGATTCTGGTATGTTCTAGCGCTTGCACCATCCCATTTAACTGTAAGAAGAATTGCAC

GGTCCCAATTGCTCGAGAGATTTCTCTTTTACCTTTTTTTACTATTTTTCACTCTCCCAT

AACCTCCTATATTGACTGATCTGTAATAACCACGATATTATTGGAATAAATAGGGGCTTG

AAATTTGGAAAAAAAAAAAAAACTGAAATATTTTCGTGATAAGTGATAGTGATATTCTTC

TTTTATTTGCTACTGTTACTAAGTCTCATGTACTAACATCGATTGCTTCATTCTTTTTGT

TGCTATATTATATGTTTAGAGGTTGCTGCTTTGGTTATTGATAACGGTTCTGGTATGTGT

AAAGCCGGTTTTGCCGGTGACGACGCTCCTCGTGCTGTCTTCCCATCTATCGTCGGTAGA

CCAAGACACCAAGGTATCATGGTCGGTATGGGTCAAAAAGACTCCTACGTTGGTGATGAA

GCTCAATCCAAGAGAGGTATCTTGACTTTACGTTACCCAATTGAACACGGTATTGTCACC

AACTGGGACGATATGGAAAAGATCTGGCATCATACCTTCTACAACGAATTGAGAGTTGCC

CCAGAAGAACACCCTGTTCTTTTGACTGAAGCTCCAATGAACCCTAAATCAAACAGAGAA

AAGATGACTCAAATTATGTTTGAAACTTTCAACGTTCCAGCCTTCTACGTTTCCATCCAA

GCCGTTTTGTCCTTGTACTCTTCCGGTAGAACTACTGGTATTGTTTTGGATTCCGGTGAT

GGTGTTACTCACGTCGTTCCAATTTACGCTGGTTTCTCTCTACCTCACGCCATTTTGAGA

ATCGATTTGGCCGGTAGAGATTTGACTGACTACTTGATGAAGATCTTGAGTGAACGTGGT

TACTCTTTCTCCACCACTGCTGAAAGAGAAATTGTCCGTGACATCAAGGAAAAACTATGT

TACGTCGCCTTGGACTTCGAACAAGAAATGCAAACCGCTGCTCAATCTTCTTCAATTGAA

AAATCCTACGAACTTCCAGATGGTCAAGTCATCACTATTGGTAACGAAAGATTCAGAGCC

CCAGAAGCTTTGTTCCATCCTTCTGTTTTGGGTTTGGAATCTGCCGGTATTGACCAAACT

ACTTACAACTCCATCATGAAGTGTGATGTCGATGTCCGTAAGGAATTATACGGTAACATC

GTTATGTCCGGTGGTACCACCATGTTCCCAGGTATTGCCGAAAGAATGCAAAAGGAAATC

ACCGCTTTGGCTCCATCTTCCATGAAGGTCAAGATCATTGCTCCTCCAGAAAGAAAGTAC

TCCGTCTGGATTGGTGGTTCTATCTTGGCTTCTTTGACTACCTTCCAACAAATGTGGATC

TCAAAACAAGAATACGACGAAAGTGGTCCATCTATCGTTCACCACAAGTGTTTCTAA

## Primer3 Output

No mispriming library specified

Using 1-based sequence positions

OLIGO [start](http://frodo.wi.mit.edu/primer3/primer3_www_results_help.html#PRIMER_START)  [len](http://frodo.wi.mit.edu/primer3/primer3_www_results_help.html#PRIMER_LEN)  [tm](http://frodo.wi.mit.edu/primer3/primer3_www_results_help.html#PRIMER_TM)  [gc%](http://frodo.wi.mit.edu/primer3/primer3_www_results_help.html#PRIMER_GC)  [any](http://frodo.wi.mit.edu/primer3/primer3_www_results_help.html#PRIMER_ANY)  [3'](http://frodo.wi.mit.edu/primer3/primer3_www_results_help.html#PRIMER_REPEAT) [seq](http://frodo.wi.mit.edu/primer3/primer3_www_results_help.html#PRIMER_OLIGO_SEQ)

LEFT PRIMER 592 20 59.84 50.00 3.00 0.00 AGAGTTGCCCCAGAAGAACA

RIGHT PRIMER 723 20 60.07 50.00 4.00 0.00 GGCTTGGATGGAAACGTAGA

SEQUENCE SIZE: 1437

INCLUDED REGION SIZE: 1437

PRODUCT SIZE: 132, PAIR ANY COMPL: 4.00, PAIR 3' COMPL: 0.00

1 ATGGATTCTGGTATGTTCTAGCGCTTGCACCATCCCATTTAACTGTAAGAAGAATTGCAC

61 GGTCCCAATTGCTCGAGAGATTTCTCTTTTACCTTTTTTTACTATTTTTCACTCTCCCAT

121 AACCTCCTATATTGACTGATCTGTAATAACCACGATATTATTGGAATAAATAGGGGCTTG

181 AAATTTGGAAAAAAAAAAAAAACTGAAATATTTTCGTGATAAGTGATAGTGATATTCTTC

241 TTTTATTTGCTACTGTTACTAAGTCTCATGTACTAACATCGATTGCTTCATTCTTTTTGT

301 TGCTATATTATATGTTTAGAGGTTGCTGCTTTGGTTATTGATAACGGTTCTGGTATGTGT

361 AAAGCCGGTTTTGCCGGTGACGACGCTCCTCGTGCTGTCTTCCCATCTATCGTCGGTAGA

421 CCAAGACACCAAGGTATCATGGTCGGTATGGGTCAAAAAGACTCCTACGTTGGTGATGAA

481 GCTCAATCCAAGAGAGGTATCTTGACTTTACGTTACCCAATTGAACACGGTATTGTCACC

541 AACTGGGACGATATGGAAAAGATCTGGCATCATACCTTCTACAACGAATTGAGAGTTGCC

>>>>>>>>>

601 CCAGAAGAACACCCTGTTCTTTTGACTGAAGCTCCAATGAACCCTAAATCAAACAGAGAA

>>>>>>>>>>>

661 AAGATGACTCAAATTATGTTTGAAACTTTCAACGTTCCAGCCTTCTACGTTTCCATCCAA

<<<<<<<<<<<<<<<<<

721 GCCGTTTTGTCCTTGTACTCTTCCGGTAGAACTACTGGTATTGTTTTGGATTCCGGTGAT

<<<

781 GGTGTTACTCACGTCGTTCCAATTTACGCTGGTTTCTCTCTACCTCACGCCATTTTGAGA

841 ATCGATTTGGCCGGTAGAGATTTGACTGACTACTTGATGAAGATCTTGAGTGAACGTGGT

901 TACTCTTTCTCCACCACTGCTGAAAGAGAAATTGTCCGTGACATCAAGGAAAAACTATGT

961 TACGTCGCCTTGGACTTCGAACAAGAAATGCAAACCGCTGCTCAATCTTCTTCAATTGAA

1021 AAATCCTACGAACTTCCAGATGGTCAAGTCATCACTATTGGTAACGAAAGATTCAGAGCC

1081 CCAGAAGCTTTGTTCCATCCTTCTGTTTTGGGTTTGGAATCTGCCGGTATTGACCAAACT

1141 ACTTACAACTCCATCATGAAGTGTGATGTCGATGTCCGTAAGGAATTATACGGTAACATC

1201 GTTATGTCCGGTGGTACCACCATGTTCCCAGGTATTGCCGAAAGAATGCAAAAGGAAATC

1261 ACCGCTTTGGCTCCATCTTCCATGAAGGTCAAGATCATTGCTCCTCCAGAAAGAAAGTAC

1321 TCCGTCTGGATTGGTGGTTCTATCTTGGCTTCTTTGACTACCTTCCAACAAATGTGGATC

1381 TCAAAACAAGAATACGACGAAAGTGGTCCATCTATCGTTCACCACAAGTGTTTCTAA

UCSC In-Silico PCR

|  |  |
| --- | --- |
|  | >[chrVI:53974-54105](http://genome.ucsc.edu/cgi-bin/hgTracks?hgsid=219399449&db=sacCer3&position=chrVI:53974-54105&hgPcrResult=pack) 132bp AGAGTTGCCCCAGAAGAACA GGCTTGGATGGAAACGTAGA  AGAGTTGCCCCAGAAGAACAccctgttcttttgactgaagctccaatgaa  ccctaaatcaaacagagaaaagatgactcaaattatgtttgaaactttca  acgttccagcctTCTACGTTTCCATCCAAGCC |

**12) YLR110C- CCW12 Chr 12**

**ACCESSION: NC_001144**

**GeneID:850800**

ATGCAATTTTCTACTGTCGCTTCTATCGCCGCTGTCGCCGCTGTCGCTTCTGCCGCTGCT

AACGTTACCACTGCTACTGTCAGCCAAGAATCTACCACTTTGGTCACCATCACTTCTTGT

GAAGACCACGTCTGTTCTGAAACTGTCTCCCCAGCTTTGGTTTCCACCGCTACCGTCACC

GTCGATGACGTTATCACTCAATACACCACCTGGTGCCCATTGACCACTGAAGCCCCAAAG

AACGGTACTTCTACTGCTGCTCCAGTTACCTCTACTGAAGCTCCAAAGAACACCACCTCT

GCTGCTCCAACTCACTCTGTCACCTCTTACACTGGTGCTGCTGCTAAGGCTTTGCCAGCT

GCTGGTGCTTTGTTGGCTGGTGCCGCTGCTTTGTTGTTGTAA

## Primer3 Output

No mispriming library specified

Using 1-based sequence positions

LEFT PRIMER 156 18 57.09 50.00 3.00 0.00 TTTGGTTTCCACCGCTAC

RIGHT PRIMER 304 20 57.93 50.00 3.00 0.00 CAGCAGAGGTGGTGTTCTTT

PRODUCT SIZE: 149, PAIR ANY COMPL: 6.00, PAIR 3' COMPL: 0.00

PRODUCT SIZE: 127, PAIR ANY COMPL: 4.00, PAIR 3' COMPL: 0.00

1 ATGCAATTTTCTACTGTCGCTTCTATCGCCGCTGTCGCCGCTGTCGCTTCTGCCGCTGCT

61 AACGTTACCACTGCTACTGTCAGCCAAGAATCTACCACTTTGGTCACCATCACTTCTTGT

121 GAAGACCACGTCTGTTCTGAAACTGTCTCCCCAGCTTTGGTTTCCACCGCTACCGTCACC

>>>>>>>>>>>>>>>>>>

181 GTCGATGACGTTATCACTCAATACACCACCTGGTGCCCATTGACCACTGAAGCCCCAAAG

241 AACGGTACTTCTACTGCTGCTCCAGTTACCTCTACTGAAGCTCCAAAGAACACCACCTCT

<<<<<<<<<<<<<<<<

301 GCTGCTCCAACTCACTCTGTCACCTCTTACACTGGTGCTGCTGCTAAGGCTTTGCCAGCT

<<<<

361 GCTGGTGCTTTGTTGGCTGGTGCCGCTGCTTTGTTGTTGTAA

UCSC In-Silico PCR

|  |  |
| --- | --- |
|  | >[chrXII:369795-369943](http://genome.ucsc.edu/cgi-bin/hgTracks?hgsid=219399449&db=sacCer3&position=chrXII:369795-369943&hgPcrResult=pack) 149bp TTTGGTTTCCACCGCTAC CAGCAGAGGTGGTGTTCTTT  TTTGGTTTCCACCGCTACcgtcaccgtcgatgacgttatcactcaataca  ccacctggtgcccattgaccactgaagccccaaagaacggtacttctact  gctgctccagttacctctactgaagctccAAAGAACACCACCTCTGCTG |

**13) YNL142W- MEP2 Chr 14**

**ACCESSION: NC_001146**

**GeneID:855580**

ATGTCTTACAATTTTACAGGTACGCCTACAGGCGAAGGAACGGGTGGTAACTCGTTGACA

ACAGATTTGAATACACAATTTGACTTGGCCAACATGGGATGGATCGGTGTGGCTTCAGCA

GGTGTGTGGATTATGGTCCCAGGTATCGGTTTATTATATTCTGGTTTATCCAGGAAAAAG

CATGCTTTATCCTTGCTTTGGGCCTCGATGATGGCTTCCGCCGTGTGTATTTTCCAATGG

TTTTTCTGGGGATACTCATTAGCTTTCTCACACAACACTAGAGGTAACGGTTTTATTGGT

ACCTTGGAATTCTTTGGGTTTCGTAACGTTTTAGGAGCCCCATCTAGTGTCAGTTCTCTT

CCCGATATACTGTTTGCCGTTTACCAAGGTATGTTTGCCGCAGTCACCGGTGCCCTAATG

CTAGGTGGTGCCTGCGAGAGGGCAAGGTTGTTTCCTATGATGGTGTTCTTGTTTTTATGG

ATGACTATTGTTTATTGTCCTATTGCATGCTGGGTCTGGAATGCCGAGGGTTGGTTGGTC

AAATTGGGTAGCTTGGACTATGCAGGTGGTTTATGTGTCCATTTAACATCTGGACATGGT

GGTCTAGTTTACGCTTTGATACTGGGTAAGCGTAATGACCCTGTTACACGTAAAGGGATG

CCCAAGTACAAACCACATTCCGTCACCTCGGTGGTTTTAGGCACAGTGTTCTTATGGTTT

GGTTGGATGTTCTTTAACGGAGGCTCTGCAGGTAATGCAACTATACGAGCATGGTACTCT

ATTATGTCCACAAACTTAGCTGCTGCTTGCGGTGGCTTGACATGGATGGTTATCGATTAT

TTCAGATGCGGAAGAAAGTGGACTACAGTTGGTTTGTGTTCAGGTATCATCGCTGGCCTA

GTGGGTATCACCCCAGCCGCCGGGTTCGTGCCAATCTGGTCAGCCGTTGTCATTGGTGTG

GTTACTGGTGCAGGATGTAACCTTGCTGTTGACTTAAAGAGTCTATTGCGCATCGATGAT

GGTCTAGATTGTTACTCTATCCATGGTGTGGGTGGTTGTATTGGTTCTGTATTAACTGGT

ATCTTTGCTGCAGACTATGTAAATGCCACTGCAGGCTCTTACATTAGTCCAATTGATGGT

GGCTGGATCAATCATCACTATAAACAAGTTGGTTATCAATTAGCAGGTATATGCGCTGCA

CTAGCCTGGACTGTTACTGTCACATCTATCTTGCTTCTAACTATGAATGCCATTCCATTT

TTAAAACTAAGATTAAGTGCTGATGAGGAAGAATTAGGTACCGACGCTGCTCAAATTGGT

GAATTTACATACGAGGAATCCACTGCTTACATCCCAGAACCAATCAGATCTAAAACATCG

GCACAAATGCCACCTCCTCATGAAAACATTGATGATAAGATTGTGGGTAACACAGACGCA

GAAAAGAATTCTACGCCTTCCGACGCTTCTTCTACTAAGAACACTGACCATATAGTATAA

## Primer3 Output

No mispriming library specified

Using 1-based sequence positions

OLIGO [start](http://frodo.wi.mit.edu/primer3/primer3_www_results_help.html#PRIMER_START)  [len](http://frodo.wi.mit.edu/primer3/primer3_www_results_help.html#PRIMER_LEN)  [tm](http://frodo.wi.mit.edu/primer3/primer3_www_results_help.html#PRIMER_TM)  [gc%](http://frodo.wi.mit.edu/primer3/primer3_www_results_help.html#PRIMER_GC)  [any](http://frodo.wi.mit.edu/primer3/primer3_www_results_help.html#PRIMER_ANY)  [3'](http://frodo.wi.mit.edu/primer3/primer3_www_results_help.html#PRIMER_REPEAT) [seq](http://frodo.wi.mit.edu/primer3/primer3_www_results_help.html#PRIMER_OLIGO_SEQ)

LEFT PRIMER 590 20 54.97 50.00 4.00 0.00 CTGGACATGGTGGTCTAGTT

RIGHT PRIMER 689 18 56.65 55.56 3.00 0.00 GAGGTGACGGAATGTGGT

SEQUENCE SIZE: 1500

INCLUDED REGION SIZE: 1500

PRODUCT SIZE: 100, PAIR ANY COMPL: 4.00, PAIR 3' COMPL: 0.00

1 ATGTCTTACAATTTTACAGGTACGCCTACAGGCGAAGGAACGGGTGGTAACTCGTTGACA

61 ACAGATTTGAATACACAATTTGACTTGGCCAACATGGGATGGATCGGTGTGGCTTCAGCA

121 GGTGTGTGGATTATGGTCCCAGGTATCGGTTTATTATATTCTGGTTTATCCAGGAAAAAG

181 CATGCTTTATCCTTGCTTTGGGCCTCGATGATGGCTTCCGCCGTGTGTATTTTCCAATGG

241 TTTTTCTGGGGATACTCATTAGCTTTCTCACACAACACTAGAGGTAACGGTTTTATTGGT

301 ACCTTGGAATTCTTTGGGTTTCGTAACGTTTTAGGAGCCCCATCTAGTGTCAGTTCTCTT

361 CCCGATATACTGTTTGCCGTTTACCAAGGTATGTTTGCCGCAGTCACCGGTGCCCTAATG

421 CTAGGTGGTGCCTGCGAGAGGGCAAGGTTGTTTCCTATGATGGTGTTCTTGTTTTTATGG

481 ATGACTATTGTTTATTGTCCTATTGCATGCTGGGTCTGGAATGCCGAGGGTTGGTTGGTC

541 AAATTGGGTAGCTTGGACTATGCAGGTGGTTTATGTGTCCATTTAACATCTGGACATGGT

>>>>>>>>>>>

601 GGTCTAGTTTACGCTTTGATACTGGGTAAGCGTAATGACCCTGTTACACGTAAAGGGATG

>>>>>>>>>

661 CCCAAGTACAAACCACATTCCGTCACCTCGGTGGTTTTAGGCACAGTGTTCTTATGGTTT

<<<<<<<<<<<<<<<<<<

721 GGTTGGATGTTCTTTAACGGAGGCTCTGCAGGTAATGCAACTATACGAGCATGGTACTCT

781 ATTATGTCCACAAACTTAGCTGCTGCTTGCGGTGGCTTGACATGGATGGTTATCGATTAT

841 TTCAGATGCGGAAGAAAGTGGACTACAGTTGGTTTGTGTTCAGGTATCATCGCTGGCCTA

901 GTGGGTATCACCCCAGCCGCCGGGTTCGTGCCAATCTGGTCAGCCGTTGTCATTGGTGTG

961 GTTACTGGTGCAGGATGTAACCTTGCTGTTGACTTAAAGAGTCTATTGCGCATCGATGAT

1021 GGTCTAGATTGTTACTCTATCCATGGTGTGGGTGGTTGTATTGGTTCTGTATTAACTGGT

1081 ATCTTTGCTGCAGACTATGTAAATGCCACTGCAGGCTCTTACATTAGTCCAATTGATGGT

1141 GGCTGGATCAATCATCACTATAAACAAGTTGGTTATCAATTAGCAGGTATATGCGCTGCA

1201 CTAGCCTGGACTGTTACTGTCACATCTATCTTGCTTCTAACTATGAATGCCATTCCATTT

1261 TTAAAACTAAGATTAAGTGCTGATGAGGAAGAATTAGGTACCGACGCTGCTCAAATTGGT

1321 GAATTTACATACGAGGAATCCACTGCTTACATCCCAGAACCAATCAGATCTAAAACATCG

1381 GCACAAATGCCACCTCCTCATGAAAACATTGATGATAAGATTGTGGGTAACACAGACGCA

1441 GAAAAGAATTCTACGCCTTCCGACGCTTCTTCTACTAAGAACACTGACCATATAGTATAA

UCSC In-Silico PCR

|  |  |
| --- | --- |
|  | >[chrXIV:358042+358141](http://genome.ucsc.edu/cgi-bin/hgTracks?hgsid=219399449&db=sacCer3&position=chrXIV:358042-358141&hgPcrResult=pack) 100bp CTGGACATGGTGGTCTAGTT GAGGTGACGGAATGTGGT  CTGGACATGGTGGTCTAGTTtacgctttgatactgggtaagcgtaatgac  cctgttacacgtaaagggatgcccaagtacaaACCACATTCCGTCACCTC |

**14) YKL109W- HAP4 Chr 11**

**ACCESSION: NC_001143**

**GeneID:853751**

ATGACCGCAAAGACTTTTCTACTACAGGCCTCCGCTAGTCGCCCTCGTAGTAACCATTTT

AAAAATGAGCATAATAATATTCCATTGGCGCCTGTACCGATCGCCCCAAATACCAACCAT

CATAACAATAGTTCGCTGGAATTCGAAAACGATGGCAGTAAAAAGAAGAAGAAGTCTAGC

TTGGTGGTTAGAACTTCAAAACATTGGGTTTTGCCCCCAAGACCAAGACCTGGTAGAAGA

TCATCTTCTCACAACACTCTACCTGCCAACAACACCAATAATATTTTAAATGTTGGCCCT

AACAGCAGGAACAGTAGTAATAATAATAATAATAATAACATCATTTCGAATAGGAAACAA

GCTTCCAAAGAAAAGAGGAAAATACCAAGACATATCCAGACAATCGATGAAAAGCTAATA

AACGACTCGAATTACCTCGCATTTTTGAAGTTCGATGACTTGGAAAATGAAAAGTTTCAT

TCTTCTGCCTCCTCCATTTCATCTCCATCTTATTCATCTCCATCTTTTTCAAGTTATAGA

AATAGAAAAAAATCAGAATTCATGGACGATGAAAGCTGCACCGATGTGGAAACCATTGCT

GCTCACAACAGTCTGCTAACAAAAAACCATCATATAGATTCTTCTTCAAATGTTCACGCA

CCACCCACGAAAAAATCAAAGTTGAACGACTTTGATTTATTGTCCTTATCTTCCACATCT

TCATCGGCCACTCCGGTCCCACAGTTGACAAAAGATTTGAACATGAACCTAAATTTTCAT

AAGATCCCTCATAAGGCTTCATTCCCTGATTCTCCAGCAGATTTCTCTCCAGCAGATTCA

GTCTCGTTGATTAGAAACCACTCCTTGCCTACTAATTTGCAAGTTAAGGACAAAATTGAG

GATTTGAACGAGATTAAATTCTTTAACGATTTCGAGAAACTTGAGTTTTTCAATAAGTAT

GCCAAAGTCAACACGAATAACGACGTTAACGAAAATAATGATCTCTGGAATTCTTACTTA

CAGTCTATGGACGATACAACAGGTAAGAACAGTGGCAATTACCAACAAGTGGACAATGAC

GATAATATGTCTTTATTGAATCTGCCAATTTTGGAGGAAACCGTATCTTCAGGGCAAGAT

GATAAGGTTGAGCCAGATGAAGAAGACATTTGGAATTATTTACCAAGTTCAAGTTCACAA

CAAGAAGATTCATCACGTGCTTTGAAAAAAAATACTAATTCTGAGAAGGCGAACATCCAA

GCAAAGAACGATGAAACCTATCTGTTTCTTCAGGATCAGGATGAAAGCGCTGATTCGCAT

CACCATGACGAGTTAGGTTCAGAAATCACTTTGGCTGACAATAAGTTTTCTTATTTGCCC

CCAACTCTAGAAGAGTTGATGGAAGAGCAGGACTGTAACAATGGCAGATCTTTTAAAAAT

TTCATGTTTTCCAACGATACCGGTATTGACGGTAGTGCCGGTACTGATGACGACTACACC

AAAGTTCTGAAATCCAAAAAAATTTCTACGTCGAAGTCGAACGCTAACCTTTATGACTTA

AACGATAACAACAATGATGCAACTGCCACCAATGAACTTGATCAAAGCAGTTTCATCGAC

GACCTTGACGAAGATGTCGATTTTTTAAAGGTACAAGTATTTTGA

## Primer3 Output

No mispriming library specified

Using 1-based sequence positions

OLIGO [start](http://frodo.wi.mit.edu/primer3/primer3_www_results_help.html#PRIMER_START)  [len](http://frodo.wi.mit.edu/primer3/primer3_www_results_help.html#PRIMER_LEN)  [tm](http://frodo.wi.mit.edu/primer3/primer3_www_results_help.html#PRIMER_TM)  [gc%](http://frodo.wi.mit.edu/primer3/primer3_www_results_help.html#PRIMER_GC)  [any](http://frodo.wi.mit.edu/primer3/primer3_www_results_help.html#PRIMER_ANY)  [3'](http://frodo.wi.mit.edu/primer3/primer3_www_results_help.html#PRIMER_REPEAT) [seq](http://frodo.wi.mit.edu/primer3/primer3_www_results_help.html#PRIMER_OLIGO_SEQ)

LEFT PRIMER 488 20 60.00 50.00 2.00 0.00 CCTCCTCCATTTCATCTCCA

RIGHT PRIMER 602 20 61.46 50.00 3.00 0.00 GCAGCAATGGTTTCCACATC

SEQUENCE SIZE: 1665

INCLUDED REGION SIZE: 1665

PRODUCT SIZE: 115, PAIR ANY COMPL: 5.00, PAIR 3' COMPL: 0.00

1 ATGACCGCAAAGACTTTTCTACTACAGGCCTCCGCTAGTCGCCCTCGTAGTAACCATTTT

61 AAAAATGAGCATAATAATATTCCATTGGCGCCTGTACCGATCGCCCCAAATACCAACCAT

121 CATAACAATAGTTCGCTGGAATTCGAAAACGATGGCAGTAAAAAGAAGAAGAAGTCTAGC

181 TTGGTGGTTAGAACTTCAAAACATTGGGTTTTGCCCCCAAGACCAAGACCTGGTAGAAGA

241 TCATCTTCTCACAACACTCTACCTGCCAACAACACCAATAATATTTTAAATGTTGGCCCT

301 AACAGCAGGAACAGTAGTAATAATAATAATAATAATAACATCATTTCGAATAGGAAACAA

361 GCTTCCAAAGAAAAGAGGAAAATACCAAGACATATCCAGACAATCGATGAAAAGCTAATA

421 AACGACTCGAATTACCTCGCATTTTTGAAGTTCGATGACTTGGAAAATGAAAAGTTTCAT

481 TCTTCTGCCTCCTCCATTTCATCTCCATCTTATTCATCTCCATCTTTTTCAAGTTATAGA

>>>>>>>>>>>>>>>>>>>>

541 AATAGAAAAAAATCAGAATTCATGGACGATGAAAGCTGCACCGATGTGGAAACCATTGCT

<<<<<<<<<<<<<<<<<<

601 GCTCACAACAGTCTGCTAACAAAAAACCATCATATAGATTCTTCTTCAAATGTTCACGCA

<<

661 CCACCCACGAAAAAATCAAAGTTGAACGACTTTGATTTATTGTCCTTATCTTCCACATCT

721 TCATCGGCCACTCCGGTCCCACAGTTGACAAAAGATTTGAACATGAACCTAAATTTTCAT

781 AAGATCCCTCATAAGGCTTCATTCCCTGATTCTCCAGCAGATTTCTCTCCAGCAGATTCA

841 GTCTCGTTGATTAGAAACCACTCCTTGCCTACTAATTTGCAAGTTAAGGACAAAATTGAG

901 GATTTGAACGAGATTAAATTCTTTAACGATTTCGAGAAACTTGAGTTTTTCAATAAGTAT

961 GCCAAAGTCAACACGAATAACGACGTTAACGAAAATAATGATCTCTGGAATTCTTACTTA

1021 CAGTCTATGGACGATACAACAGGTAAGAACAGTGGCAATTACCAACAAGTGGACAATGAC

1081 GATAATATGTCTTTATTGAATCTGCCAATTTTGGAGGAAACCGTATCTTCAGGGCAAGAT

1141 GATAAGGTTGAGCCAGATGAAGAAGACATTTGGAATTATTTACCAAGTTCAAGTTCACAA

1201 CAAGAAGATTCATCACGTGCTTTGAAAAAAAATACTAATTCTGAGAAGGCGAACATCCAA

1261 GCAAAGAACGATGAAACCTATCTGTTTCTTCAGGATCAGGATGAAAGCGCTGATTCGCAT

1321 CACCATGACGAGTTAGGTTCAGAAATCACTTTGGCTGACAATAAGTTTTCTTATTTGCCC

1381 CCAACTCTAGAAGAGTTGATGGAAGAGCAGGACTGTAACAATGGCAGATCTTTTAAAAAT

1441 TTCATGTTTTCCAACGATACCGGTATTGACGGTAGTGCCGGTACTGATGACGACTACACC

1501 AAAGTTCTGAAATCCAAAAAAATTTCTACGTCGAAGTCGAACGCTAACCTTTATGACTTA

1561 AACGATAACAACAATGATGCAACTGCCACCAATGAACTTGATCAAAGCAGTTTCATCGAC

1621 GACCTTGACGAAGATGTCGATTTTTTAAAGGTACAAGTATTTTGA

UCSC In-Silico PCR

|  |  |
| --- | --- |
|  | >[chrXI:232714+232828](http://genome.ucsc.edu/cgi-bin/hgTracks?hgsid=219399449&db=sacCer3&position=chrXI:232714-232828&hgPcrResult=pack) 115bp CCTCCTCCATTTCATCTCCA GCAGCAATGGTTTCCACATC  CCTCCTCCATTTCATCTCCAtcttattcatctccatctttttcaagttat  agaaatagaaaaaaatcagaattcatggacgatgaaagctgcaccGATGT  GGAAACCATTGCTGC |

**15) RDN18-1; RDN18-2**

**Accession:NC_001144.5**

**Gene ID: 9164923; 9164932**

TAATGATCCTTCCGCAGGTTCACCTACGGAAACCTTGTTACGACTTTTAGTTCCTCTAAATGACCAAGTTTGTCCAAATTCTCCGCTCTGAGATGGAGTTGCCCCCTTCTCTAAGCAGATCCTGAGGCCTCACTAAGCCATTCAATCGGTACTAGCGACGGGCGGTGTGTACAAAGGGCAGGGACGTAATCAACGCAAGCTGATGACTTGCGCTTACTAGGAATTCCTCGTTGAAGAGCAATAATTACAATGCTCTATCCCCAGCACGACGGAGTTTCACAAGATTACCAAGACCTCTCGGCCAAGGTTAGACTCGCTGGCTCCGTCAGTGTAGCGCGCGTGCGGCCCAGAACGTCTAAGGGCATCACAGACCTGTTATTGCCTCAAACTTCCATCGGCTTGAAACCGATAGTCCCTCTAAGAAGTGGATAACCAGCAAATGCTAGCACCACTATTTAGTAGGTTAAGGTCTCGTTCGTTATCGCAATTAAGCAGACAAATCACTCCACCAACTAAGAACGGCCATGCACCACCACCCACAAAATCAAGAAAGAGCTCTCAATCTGTCAATCCTTATTGTGTCTGGACCTGGTGAGTTTCCCCGTGTTGAGTCAAATTAAGCCGCAGGCTCCACTCCTGGTGGTGCCCTTCCGTCAATTCCTTTAAGTTTCAGCCTTGCGACCATACTCCCCCCAGAACCCAAAGACTTTGATTTCTCGTAAGGTGCCGAGTGGGTCATTAAAAAAACACCACCCGATCCCTAGTCGGCATAGTTTATGGTTAAGACTACGACGGTATCTGATCATCTTCGATCCCCTAACTTTCGTTCTTGATTAATGAAAACGTCCTTGGCAAATGCTTTCGCAGTAGTTAGTCTTCAATAAATCCAAGAATTTCACCTCTGACAATTGAATACTGATGCCCCCGACCGTCCCTATTAATCATTACGATGGTCCTAGAAACCAACAAAATAGAACCAAACGTCCTATTCTATTATTCCATGCTAATATATTCGAGCAATACGCCTGCTTTGAACACTCTAATTTTTTCAAAGTAAAAGTCCTGGTTCGCCAAGAGCCACAAGGACTCAAGGTTAGCCAGAAGGAAAGGCCCCGTTGGAAATCCAGTACACGAAAAAATCGGACCGGCCAACCGGGCCCAAAGTTCAACTACGAGCTTTTTAACTGCAACAACTTTAATATACGCTATTGGAGCTGGAATTACCGCGGCTGCTGGCACCAGACTTGCCCTCCAATTGTTCCTCGTTAAGGTATTTACATTGTACTCATTCCAATTACAAGACCCGAATGGGCCCTGTATCGTTATTTATTGTCACTACCTCCCTGAATTAGGATTGGGTAATTTGCGCGCCTGCTGCCTTCCTTGGATGTGGTAGCCGTTTCTCAGGCTCCCTCTCCGGAATCGAACCCTTATTCCCCGTTACCCGTTGAAACCATGGTAGGCCACTATCCTACCATCGAAAGTTGATAGGGCAGAAATTTGAATGAACCATCGCCAGCACAAGGCCATGCGATTCGAAAAGTTATTATGAATCATCAAAGAGTCCGAAGACATTGATTTTTTATCTAATAAATACATCTCTTCCAAAGGGTCGAGATTTTAAGCATGTATTAGCTCTAGAATTACCACAGTTATACCATGTAGTAAAGGAACTATCAAATAAACGATAACTGATTTAATGAGCCATTCGCAGTTTCACTGTATAAATTGCTTATACTTAGACATGCATGGCTTAATCTTTGAGACAAGCATATGACTACTGGCAGGATCAACCAGATA
