# Supplementary material for: A Novel Strategy for Selection and Validation of Reference Genes in Dynamic Multidimensional Experimental Design in Yeast
Source: PLoS One. 2012 Jun 4;7(6):e38351. doi: 10.1371/journal.pone.0038351 (PMC3366934; doi:10.1371/journal.pone.0038351)

**CASE STUDY I**

PGK1-TPI1


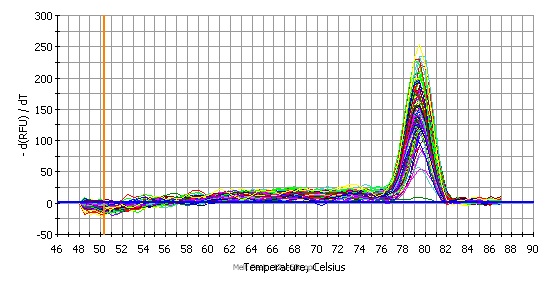


TDH3


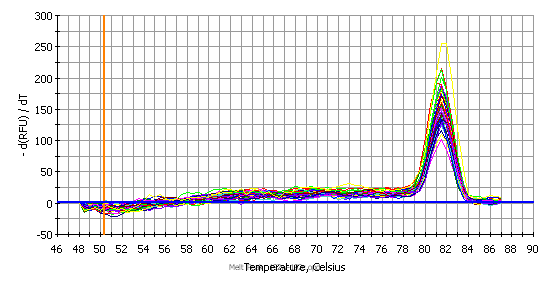


FBA1-GCN4


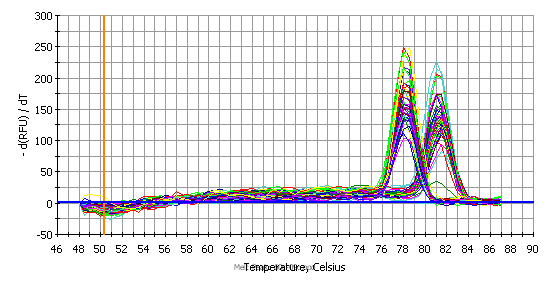


ACT1-ADH1


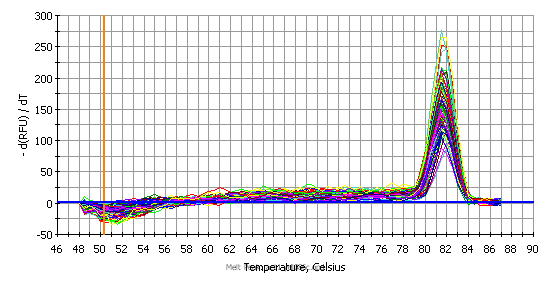


CDC19-PDC1


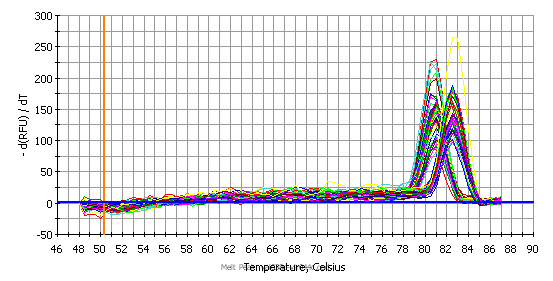


ARF1-RPS26A


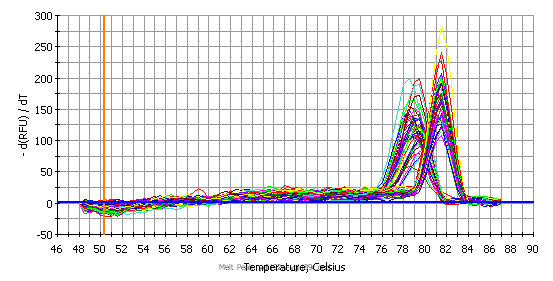


CCW12


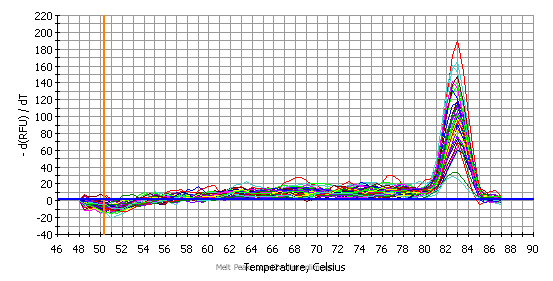


HAP4


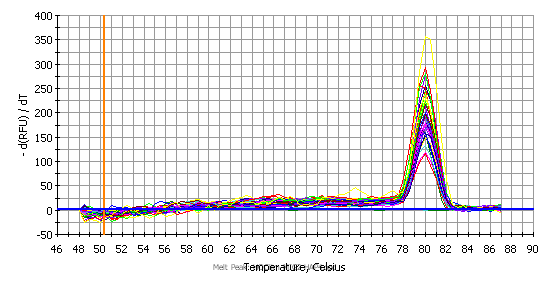


**CASE STUDY II**

GCN4-ACT1


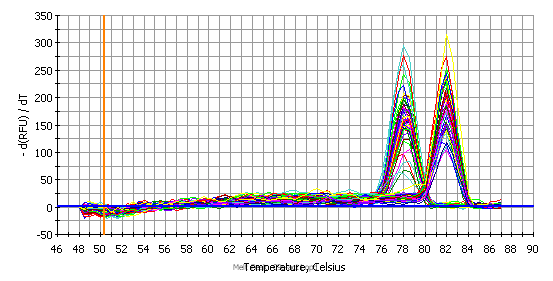


PGK1


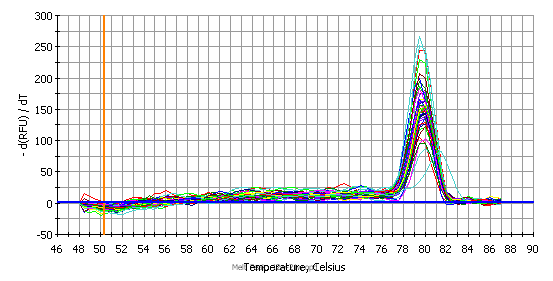


RPS26A-FBA1


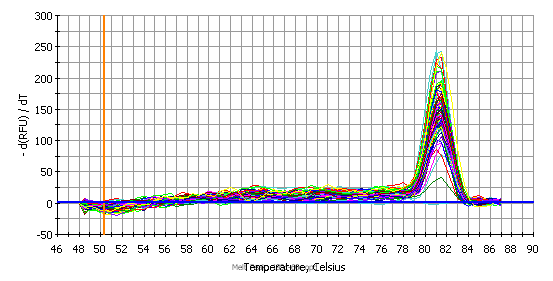


ARF1-TDH3


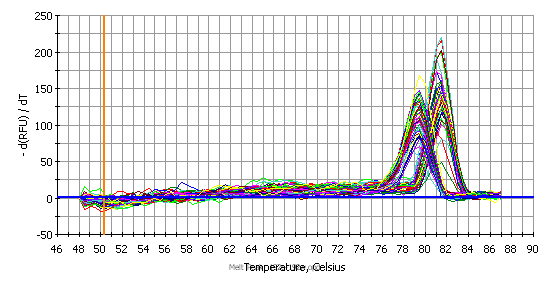


CDC19-TPI1


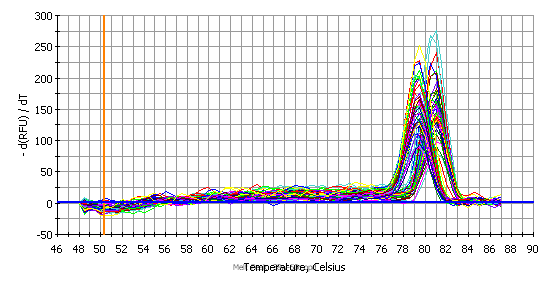


ADH1-PDC1


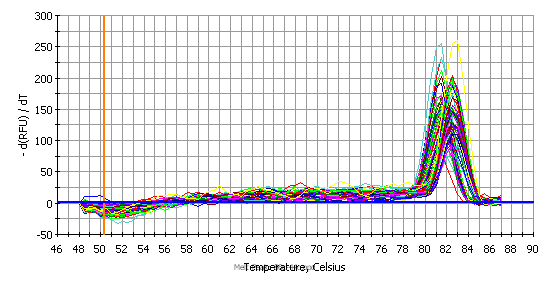


CCW12


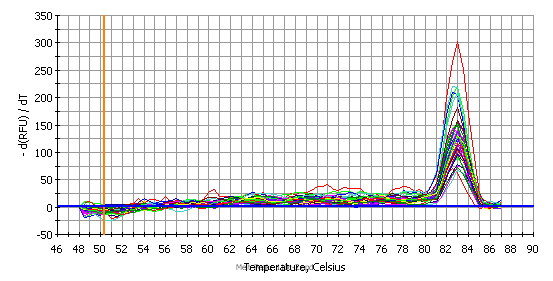


MEP2


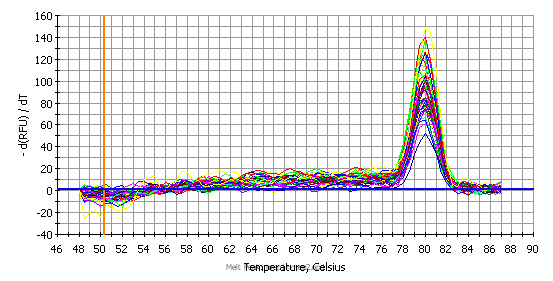

Supplement: Text S3 — Results of the melt curve analysis. (DOC) [file pone.0038351.s026.doc]
