# Supplementary material for: A Novel Strategy for Selection and Validation of Reference Genes in Dynamic Multidimensional Experimental Design in Yeast
Source: PLoS One. 2012 Jun 4;7(6):e38351. doi: 10.1371/journal.pone.0038351 (PMC3366934; doi:10.1371/journal.pone.0038351)
Supplement: Supplementary file 13 [file pone.0038351.s024.doc]

Assay Class: Plant RNA Nano
Data Path: C:\...00 expert_Plant RNA Nano_DE72902267_2010-09-28_15-59-41.xad

Created: 9/28/2010 3:59:41 PM
Modified: 9/28/2010 4:23:47 PM

Electrophoresis File Run Summary

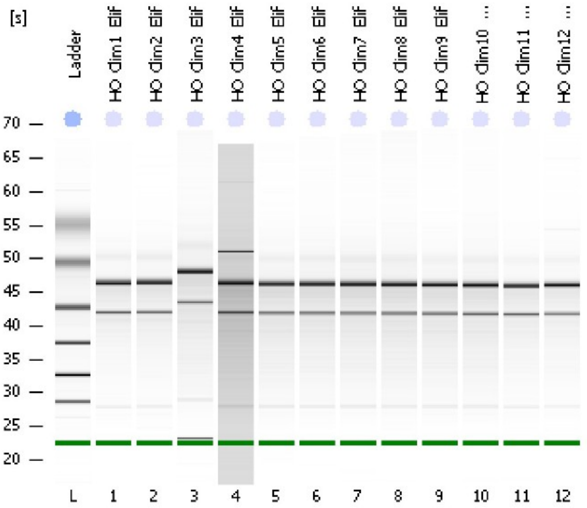

<u>Instrument Information:</u>

Instrument Name: BUCHE441
Serial#: DE72902267
Firmware: C.01.069
Type: G2939A

<u>Assay Information:</u>

Assay Origin Path: C:\Program Files\Agilent\2100 bioanalyzer\2100 expert\assays\RNA\Plant RNA Nano.xsy

Assay Class: Plant RNA Nano

Version: 1.2

Assay Comments: Total RNA Analysis ng sensitivity (Plant)

© Copyright 2003 - 2009 Agilent Technologies, Inc.

<u>Chip Information:</u>

Chip Lot #:

Reagent Kit Lot #:

Chip Comments:

HO_Clim1_Elif

RIN: 8.20

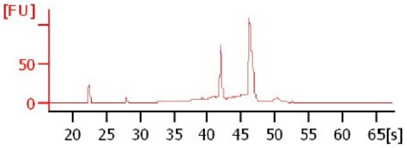

HO_Clim2_Elif

RIN: 8.10

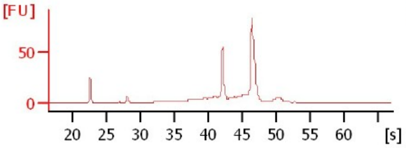

HO_Clim3_Elif

RIN: 7.60

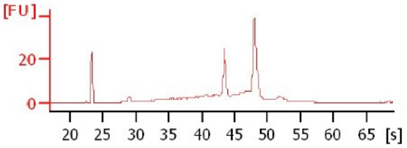

HO_Clim4_Elif

RIN: 7.30

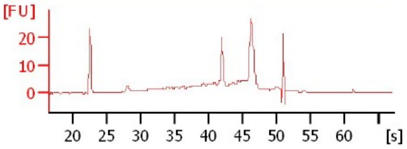

HO_Clim5_Elif

RIN: 7.60

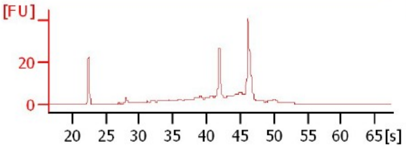

HO_Clim6_Elif

RIN: 7.70

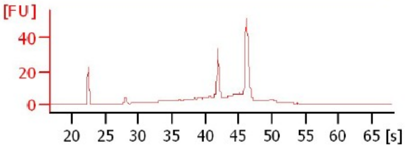

HO_Clim7_Elif

RIN: 7.90

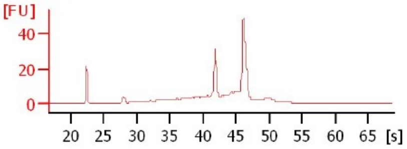

HO_Clim8_Elif

RIN: 7.60

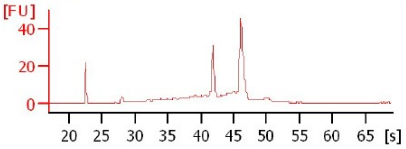

HO_Clim9_Elif

RIN: 7.50

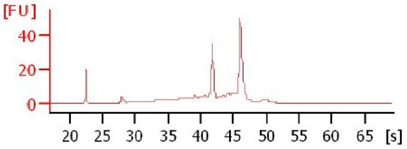

HO_Clim10_Elif

RIN: 7.60

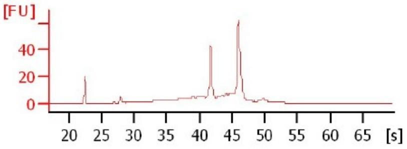

HO_Clim11_Elif

RIN: 7.90

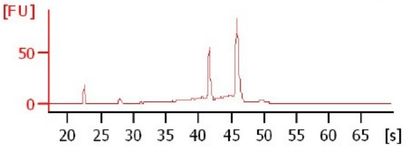

HO_Clim12_Elif

RIN: 8.20

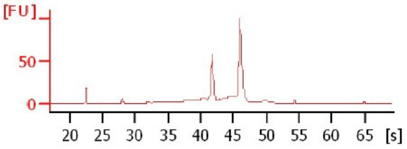

 © Copyright 2003 - 2009 Agilent Technologies, Inc.

Assay Class: Plant RNA Nano
Data Path: C:\...00 expert_Plant RNA Nano_DE72902267_2010-09-28_15-59-41.xad

Created: 9/28/2010 3:59:41 PM
Modified: 9/28/2010 4:23:47 PM

Electrophoresis File Run Summary (Chip Summary)

Sample Name	Sample Comment	Status	Result Label	Result Color
HO_Clim1_Elif		✔	RIN: 8.20	
HO_Clim2_Elif		✔	RIN: 8.10	
HO_Clim3_Elif		✔	RIN: 7.60	
HO_Clim4_Elif		✔	RIN: 7.30	
HO_Clim5_Elif		✔	RIN: 7.60	
HO_Clim6_Elif		✔	RIN: 7.70	
HO_Clim7_Elif		✔	RIN: 7.90	
HO_Clim8_Elif		✔	RIN: 7.60	
HO_Clim9_Elif		✔	RIN: 7.50	
HO_Clim10_Elif		✔	RIN: 7.60	
HO_Clim11_Elif		✔	RIN: 7.90	
HO_Clim12_Elif		✔	RIN: 8.20	

Chip Lot # **Reagent Kit Lot #**

Chip Comments :

 © Copyright 2003 - 2009 Agilent Technologies, Inc.

Assay Class: Plant RNA Nano
Data Path: C:\...00 expert_Plant RNA Nano_DE72902267_2010-09-28_15-59-41.xad
Created: 9/28/2010 3:59:41 PM
Modified: 9/28/2010 4:23:47 PM

Electrophoresis Assay Details

General Analysis Settings

Number of Available Sample and Ladder Wells (Max.) : 13
Minimum Visible Range [s] : 17
Maximum Visible Range [s] : 70
Start Analysis Time Range [s] : 19
End Analysis Time Range [s] : 69
Ladder Concentration [ng/µl] : 150
Lower Marker Concentration [ng/µl] : 0
Upper Marker Concentration [ng/µl] : 0
Used Lower Marker for Quantitation
Standard Curve Fit is Logarithmic
Show Data Aligned to Lower Marker

Integrator Settings

Integration Start Time [s] : 19
Integration End Time [s] : 69
Slope Threshold : 0.6
Height Threshold [FU] : 0.2
Area Threshold : 0.1
Width Threshold [s] : 0.2
Baseline Plateau [s] : 6

Filter Settings

Filter Width [s] : 0.5
Polynomial Order : 4

Ladder

Ladder Peak	Size
1	25
2	200
3	500
4	1000
5	2000
6	4000

 © Copyright 2003 - 2009 Agilent Technologies, Inc.

Assay Class: Plant RNA Nano
Data Path: C:\...00 expert_Plant RNA Nano_DE72902267_2010-09-29_18-15-59.xad

Created: 9/29/2010 6:15:59
Modified: 9/29/2010 6:39:59

Electrophoresis File Run Summary

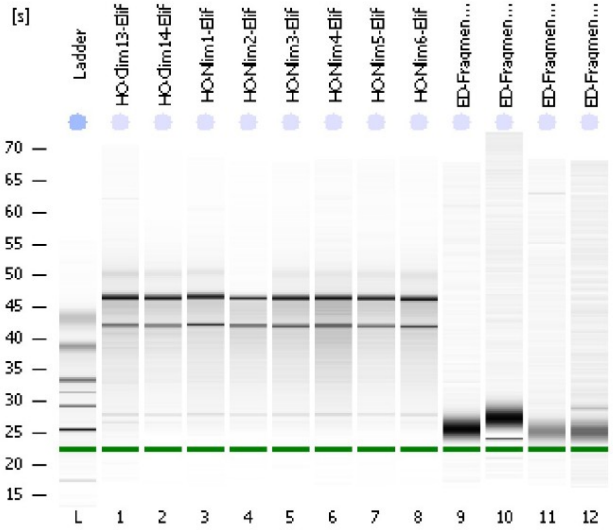

<u>Instrument Information:</u>

Instrument Name: BUCHE441 Firmware: C.01.069

Serial#: DE72902267 Type: G2939A

<u>Assay Information:</u>

Assay Origin Path: C:\Program Files\Agilent\2100 bioanalyzer\2100 expert\assays\RNA\Plant RNA Nano.xsy

Assay Class: Plant RNA Nano

Version: 1.2

Assay Comments: Total RNA Analysis ng sensitivity (Plant)

© Copyright 2003 - 2009 Agilent Technologies, Inc.

<u>Chip Information:</u>

Chip Lot #:

Reagent Kit Lot #:

Chip Comments:

HO-Clim13-Elif

RIN: 7.10

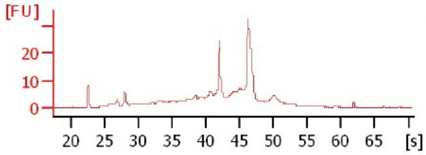

HO-Clim14-Elif

RIN: 7.20

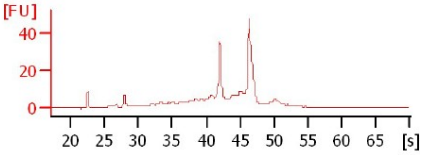

HO-Nlim1-Elif

RIN: 7.30

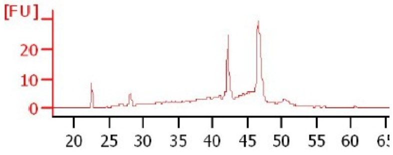

HO-Nlim2-Elif

RIN: 6.40

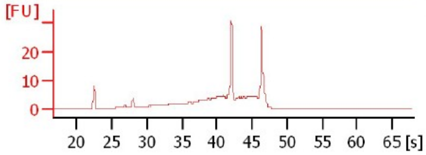

HO-Nlim3-Elif

RIN:7

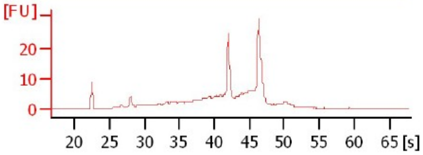

HO-Nlim4-Elif

RIN: 6.70

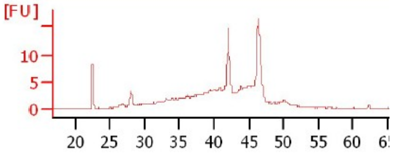

HO-Nlim5-Elif

RIN: 7.60

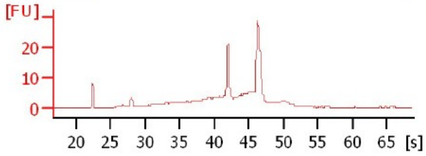

HO-Nlim6-Elif

RIN: 7.50

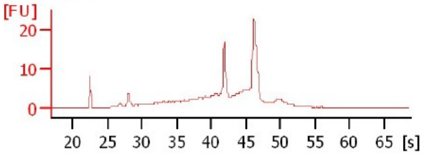

ED-Fragment31_2-2

RIN: 2.60

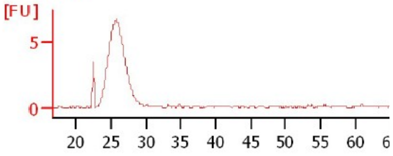

ED-Fragment33_2-2

RIN: 2.40

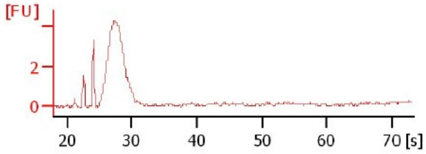

ED-Fragment31_2-repeat

RIN: 2.50

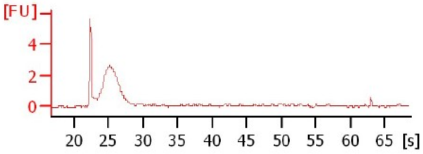

ED-Fragment33_2-repeat

RIN: 2.50

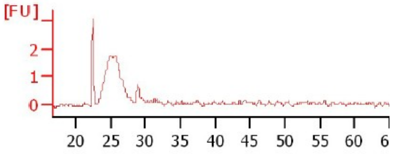

 © Copyright 2003 - 2009 Agilent Technologies, Inc.

Assay Class:	Plant RNA Nano	Created:	9/29/2010 6:1
Data Path:	C:\...00 expert_Plant RNA Nano_DE72902267_2010-09-29_18-15-59.xad	Modified:	9/29/2010 6:3

Electrophoresis File Run Summary (Chip Summary)

Sample Name	Sample Comment	Status	Result Label	Result Color
HO-Clim13-Elif		✓	RIN: 7.10	
HO-Clim14-Elif		✓	RIN: 7.20	
HO-Nlim1-Elif		✓	RIN: 7.30	
HO-Nlim2-Elif		✓	RIN: 6.40	
HO-Nlim3-Elif		✓	RIN:7	
HO-Nlim4-Elif		✓	RIN: 6.70	
HO-Nlim5-Elif		✓	RIN: 7.60	
HO-Nlim6-Elif		✓	RIN: 7.50	
ED-Fragment31_2-2		✓	RIN: 2.60	
ED-Fragment33_2-2		✓	RIN: 2.40	
ED-Fragment31_2-repeat		✓	RIN: 2.50	
ED-Fragment33_2-repeat		✓	RIN: 2.50	

Chip Lot #

Reagent Kit Lot #

Chip Comments :

Electrophoresis Assay Details

General Analysis Settings

Number of Available Sample and Ladder Wells (Max.) : 13
Minimum Visible Range [s] : 17
Maximum Visible Range [s] : 70
Start Analysis Time Range [s] : 19
End Analysis Time Range [s] : 69
Ladder Concentration [ng/µl] : 150
Lower Marker Concentration [ng/µl] : 0
Upper Marker Concentration [ng/µl] : 0
Used Lower Marker for Quantitation
Standard Curve Fit is Logarithmic
Show Data Aligned to Lower Marker

Integrator Settings

Integration Start Time [s] : 19
Integration End Time [s] : 69
Slope Threshold : 0.6
Height Threshold [FU] : 0.2
Area Threshold : 0.1
Width Threshold [s] : 0.2
Baseline Plateau [s] : 6

Filter Settings

Filter Width [s] : 0.5
Polynomial Order : 4

Ladder

Ladder Peak	Size
1	25
2	200
3	500
4	1000
5	2000
6	4000

Assay Class: Plant RNA Nano
Data Path: C:\...00 expert_Plant RNA Nano_DE72902267_2010-10-01_15-01-18.xad

Created: 10/1/2010 3:01:18 PM
Modified: 10/1/2010 3:25:21 PM

Electrophoresis File Run Summary

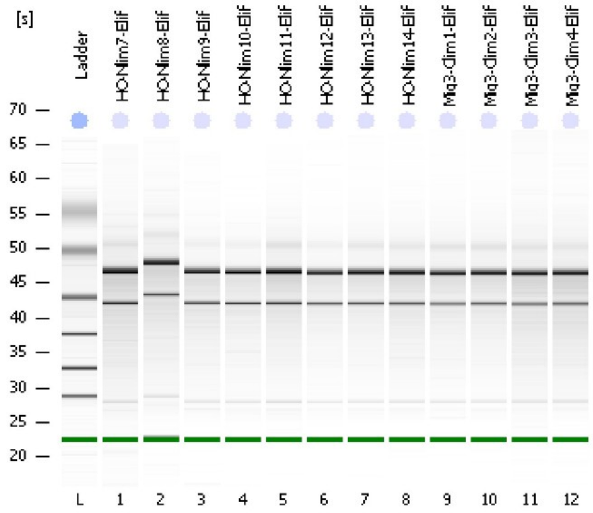

__Instrument Information:__

Instrument Name: BUCHE441
Serial#: DE72902267
Firmware: C.01.069
Type: G2939A

__Assay Information:__

Assay Origin Path: C:\Program Files\Agilent\2100 bioanalyzer\2100 expert\assays\RNA\Plant RNA Nano.xsy

Assay Class: Plant RNA Nano

Version: 1.2

Assay Comments: Total RNA Analysis ng sensitivity (Plant)

© Copyright 2003 - 2009 Agilent Technologies, Inc.

__Chip Information:__

Chip Lot #:

Reagent Kit Lot #:

Chip Comments:

HO-Nlim7-Elif

RIN: 7.20

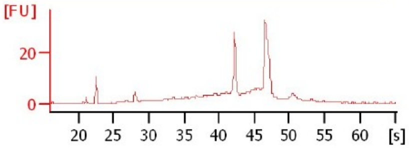

HO-Nlim8-Elif

RIN: 7.10

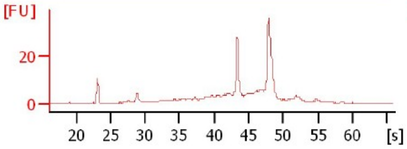

HO-Nlim9-Elif

RIN: 7.40

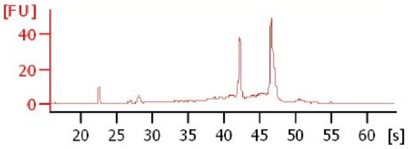

HO-Nlim10-Elif

RIN: 7.70

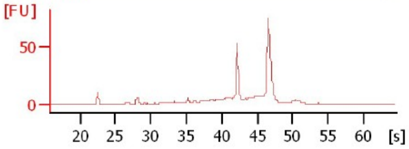

HO-Nlim11-Elif

RIN: 7.90

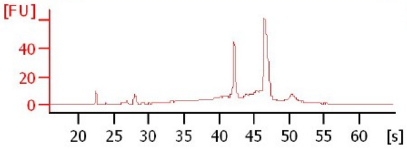

HO-Nlim12-Elif

RIN:8

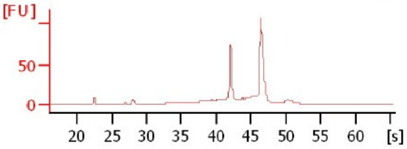

HO-Nlim13-Elif

RIN: 8.10

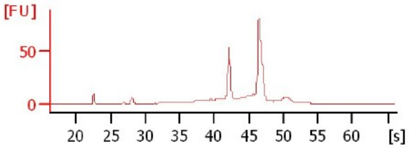

HO-Nlim14-Elif

RIN: 7.90

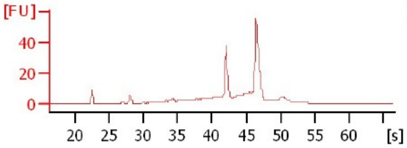

Mig3-Clim1-Elif

RIN: 8.10

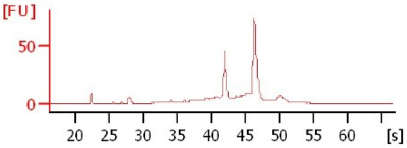

Assay Class: Plant RNA Nano
Data Path: C:\...00 expert_Plant RNA Nano_DE72902267_2010-10-01_15-01-18.xad
Created: 10/1/2010 3:01:18 PM
Modified: 10/1/2010 3:25:21 PM

Electrophoresis File Run Summary (Chip Summary)

Sample Name	Sample Comment	Status	Result Label	Result Color
HO-Nlim7-Elif		✔	RIN: 7.20	
HO-Nlim8-Elif		✔	RIN: 7.10	
HO-Nlim9-Elif		✔	RIN: 7.40	
HO-Nlim10-Elif		✔	RIN: 7.70	
HO-Nlim11-Elif		✔	RIN: 7.90	
HO-Nlim12-Elif		✔	RIN:8	
HO-Nlim13-Elif		✔	RIN: 8.10	
HO-Nlim14-Elif		✔	RIN: 7.90	

Assay Class: Plant RNA Nano
Data Path: C:\...00 expert_Plant RNA Nano_DE72902267_2010-10-01_15-01-18.xad
Created: 10/1/2010 3:01:
Modified: 10/1/2010 3:25:

Electrophoresis Assay Details

General Analysis Settings

Number of Available Sample and Ladder Wells (Max.) : 13
Minimum Visible Range [s] : 17
Maximum Visible Range [s] : 70
Start Analysis Time Range [s] : 19
End Analysis Time Range [s] : 69
Ladder Concentration [ng/µl] : 150
Lower Marker Concentration [ng/µl] : 0
Upper Marker Concentration [ng/µl] : 0
Used Lower Marker for Quantitation
Standard Curve Fit is Logarithmic
Show Data Aligned to Lower Marker

Integrator Settings

Integration Start Time [s] : 19
Integration End Time [s] : 69
Slope Threshold : 0.6
Height Threshold [FU] : 0.2
Area Threshold : 0.1
Width Threshold [s] : 0.2
Baseline Plateau [s] : 6

Filter Settings

Filter Width [s] : 0.5
Polynomial Order : 4

Ladder

Ladder Peak	Size
1	25
2	200
3	500
4	1000
5	2000
6	4000

 © Copyright 2003 - 2009 Agilent Technologies, Inc.
